# RNA G-quadruplex structure-based PROTACs for targeted DHX36 protein degradation and gene activity modulation in mammalian cells

**DOI:** 10.1093/nar/gkaf039

**Published:** 2025-01-30

**Authors:** Kun Zhang, Qichang Nie, Maolin Li, Xiaona Chen, Liting Zhong, Tianle Dai, Xiaofan Guo, Haizhou Zhao, Terrence Chi-Kong Lau, Huating Wang, Shuo-Bin Chen, Chun Kit Kwok

**Affiliations:** Department of Chemistry and State Key Laboratory of Marine Pollution, City University of Hong Kong, Hong Kong SAR 999077, China; Department of Biomedical Sciences, College of Veterinary Medicine and Life Sciences, City University of Hong Kong, Hong Kong SAR 999077, China; Guangdong Provincial Key Laboratory of New Drug Design and Evaluation, School of Pharmaceutical Sciences, Sun Yat-sen University, Guangzhou 510006, China; Department of Orthopaedics and Traumatology, Li Ka Shing Institute of Health Sciences, Chinese University of Hong Kong, Hong Kong SAR 999077, China; Guangdong Provincial Key Laboratory of New Drug Design and Evaluation, School of Pharmaceutical Sciences, Sun Yat-sen University, Guangzhou 510006, China; Guangdong Provincial Key Laboratory of New Drug Design and Evaluation, School of Pharmaceutical Sciences, Sun Yat-sen University, Guangzhou 510006, China; Department of Orthopaedics and Traumatology, Li Ka Shing Institute of Health Sciences, Chinese University of Hong Kong, Hong Kong SAR 999077, China; Department of Chemistry and State Key Laboratory of Marine Pollution, City University of Hong Kong, Hong Kong SAR 999077, China; Department of Biomedical Sciences, College of Veterinary Medicine and Life Sciences, City University of Hong Kong, Hong Kong SAR 999077, China; Department of Orthopaedics and Traumatology, Li Ka Shing Institute of Health Sciences, Chinese University of Hong Kong, Hong Kong SAR 999077, China; Guangdong Provincial Key Laboratory of New Drug Design and Evaluation, School of Pharmaceutical Sciences, Sun Yat-sen University, Guangzhou 510006, China; Department of Chemistry and State Key Laboratory of Marine Pollution, City University of Hong Kong, Hong Kong SAR 999077, China; Shenzhen Research Institute of City University of Hong Kong, Shenzhen 518057, China

## Abstract

RNA G-quadruplexes (rG4s) are non-canonical secondary nucleic acid structures found in the transcriptome. They play crucial roles in gene regulation by interacting with G4-binding proteins (G4BPs) in cells. rG4-G4BP complexes have been associated with human diseases, making them important targets for drug development. Generating innovative tools to disrupt rG4-G4BP interactions will provide a unique opportunity to explore new biological mechanisms and potentially treat related diseases. Here, we have rationally designed and developed a series of rG4-based proteolytic targeting chimeras (rG4-PROTACs) aimed at degrading G4BPs, such as DHX36, a specific G4BP that regulates gene expression by binding to and unraveling rG4 structures in messenger RNAs (mRNAs). Our comprehensive data and systematic analysis reveals that rG4-PROTACs predominantly and selectively degrade DHX36 through a proteosome-dependent mechanism, which promotes the formation of the rG4 structure in mRNA, leading to the translation inhibition of rG4-containing transcripts. Notably, rG4-PROTACs inhibit rG4-mediated APP protein expression, and impact the proliferative capacity of skeletal muscle stem cells by negatively regulating Gnai2 protein expression. In summary, rG4-PROTACs provide a new avenue to understand rG4-G4BP interactions and the biological implications of dysregulated G4BPs, promoting the development of PROTACs technology based on the non-canonical structure of nucleic acids.

## Graphical Abstract

**Graphical Abstract:**
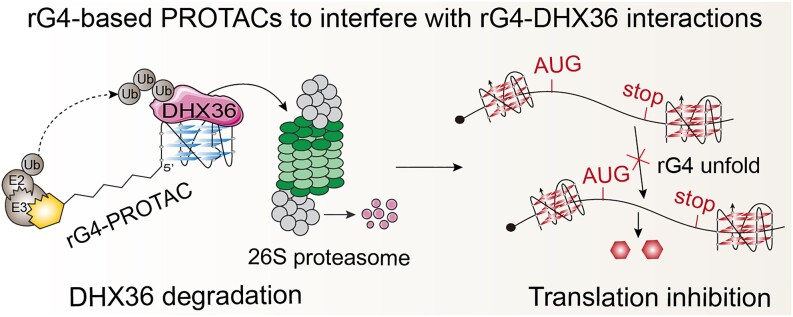

## Introduction

RNAs fold into complex and dynamic structures, allowing them to interact with proteins in cells to perform unique functions [1]. RNA G-quadruplex (rG4) is a crucial secondary structure of nucleic acids assembled by multiple G-tetrads [2]. Each G-quartet is composed of four guanines arranged via Hoogsteen hydrogen bonds and a central cation (K^+^>Na^+^>Li^+^) coordinates with the G-quartets to stabilize the rG4 structure [3, 4]. A canonical rG4 sequence consists of four consecutive guanine sequences separated by three runs of 1–7 nucleotides in length (GGGN_1–7_GGGN_1–7_GGGN_1–7_GGG) except for a few special rG4s [5, 6]. In recent years, many non-canonical rG4s subtypes, including long loops, bulges, and two quartets, have been reported [7], and these interesting RNA secondary structures can affect cellular processes [8, 9]. For instance, studies have reported that rG4s play an important role in genome regulation [8], including telomere maintenance [10], post-transcriptional regulation of messenger RNA (mRNA), and maturation of non-coding RNA [11–13].

The G4-binding protein (G4BP) is a pivotal player in regulating rG4-mediated gene expression and has emerged as an attractive target for innovative drug development [14, 15]. DEAH-Box helicase 36 (DHX36), a specialized helicase for the G4 structures [16, 17], has been shown to bind and unfold rG4 [11, 18–20], linking it closely to various human diseases. For example, DHX36 has been shown to be over-expressed in several cancer cells, particularly lung cancer and cervical cancer [21]. Additionally, studies have shown that DHX36 interacts with the G4-containing long non-coding RNA FLJ39051, facilitating increased migration of colon cancer cells [10]. Previously, we have demonstrated that DHX36 unfolds rG4 in the 5′-untranslated region (5′ UTR) and specifically regulates the translation of guanine nucleotide-binding protein G(i) subunit alpha-2 (*Gnai2*) mRNA, which is crucial for the regenerative ability of skeletal muscle stem cells (SCs) [20]. Furthermore, we found a long non-coding RNA named lockD interacts with DHX36 to form a lockD/DHX36/Anp32e complex, which promotes myoblast proliferation and muscle regeneration by facilitating the translation of Anp32e through the unwinding the 5′ UTR rG4 motif [22]. More recently, we identified an rG4 within the 5′ UTR of ADAR1 (Adenosine Deaminase Acting on RNA, RNA adenosine deaminase) and demonstrated that DHX36 modulates its expression by targeting this rG4 motif [11]. These findings position DHX36 as a key regulator in the translation of rG4-containing transcripts, leveraging DHX36 as a therapeutic target for controlling rG4-mediated gene expression in cells.

Proteolytic targeting chimeras (PROTACs) hijack the endogenous ubiquitin-proteasome system to degrade target proteins, providing a new therapeutic mode for nucleic acid-binding proteins, especially undruggable proteins [23]. PROTACs consist of two covalently linked ligand molecules: one binds to the E3 ubiquitin ligase and the other to the target protein [24]. A single PROTAC molecule can perform catalytic reaction iteratively, enabling the ubiquitination reaction to be turned around multiple times, resulting in a multi-ubiquitin chain in proteins that is subsequently recognized by the proteasome and rapidly degraded into short peptide residues (usually 3–25 in length) [25]. Currently, the ligands for the protein of interest in PROTACs are predominantly based on peptides and fully synthesized small molecules [25, 26]. Recently, PROTACs use nucleic acid motifs as warheads, have been successfully applied to degrade proteins that lack ligand-binding pockets, including RNA-binding proteins (RBPs) [27] and transcription factors [28, 29]. In addition to intracellular proteins, nucleic acid-based PROTACs have also been used to degrade membrane proteins [30]. PROTACs with aptamer AS1411(AS) as warheads have been developed into a tumor-specific targeted degradation tool [31, 32]. These studies highlighted the feasibility of targeting protein degradation mediated by nucleic acid motifs. Phan lab designed a DNA G4 (dG4) based PROTACs to deplete DHX36 protein through proteasome [33], which showed that PROTAC could be applied to target G4BP. However, the gene regulation ability and the biological potential of dG4-PROTACs by targeting DHX36 were not investigated in that study and therefore remained elusive. Besides that, our understanding of rG4 and its interacting partners is quite limited, as such developing novel molecular tools to identify and interfere with rG4-G4BP interactions is urgently needed. Moreover, the binding affinity of dG4-PROTACs needs to be further improved. We envisioned whether rG4 motifs could be introduced and designed as warheads to develop rG4-PROTACs with stronger binding affinity and regulate rG4-G4BP interactions in human and mouse cells for the first time.

Herein, we present novel rG4-PROTACs created by conjugating the *hTERC* rG4 motif with the von Hippel−Lindau (VHL) ligand through click chemistry. Upon rG4-PROTACs’ treatment in cells, rG4 moiety directly interacts with G4BPs, with DHX36 serving as a specific example, while the VHL ligand moiety recruits the E3 ligase to facilitate the ubiquitination and subsequent degradation of DHX36 protein. We demonstrate that rG4-PROTACs exhibit significantly greater binding and degradation efficiency of DHX36 compared to dG4-PROTACs. Moreover, we show that rG4-PROTACs can regulate the expression of rG4-mediated genes, such as *APP* and *Gnai2*, by degrading DHX36 in mammalian cells. Importantly, our findings indicate that rG4-PROTACs impair the proliferative capacity of skeletal muscle SCs by downregulating *Gnai2* protein expression.

## Materials and methods

### Materials

All reagents and solvents used in the organic synthesis were commercially available without further purification. Detailed compound synthesis methods can be found in the Supporting Information. (S, R, S)-AHPC-PEG_2_-N_3_ (Cas: 2010159-45-0) and Pomalidomide-PEG_1_-C_2_-N_3_ (Cas: 2271036-44-1) were purchased from MedChemExpress (USA). MG132 (Cas: 133407-82-6) was purchased from TargetMol (USA). 5′Hexynyl oligonucleotides, *APP* rG4 WT_ polyA, Trap RNA, FAM_*APP* rG4 WT, FAM_*APP* rG4 Mut, FAM_*Gnai2* rG4 WT, and FAM_*Gnai2* rG4 Mut oligonucleotides (Supplementary Table S1) were manufactured by Integrated DNA Technologies (USA). Zymo-Spin IC Column used for rG4-PROTACs conjugates purification was purchased from Zymo Research (USA). The psiCHECK-2 vector and Dual-Luciferase Reporter System E1910 were purchased from Promega (USA). Lipofectamine® 2000 and Lipofectamine® 3000 were purchased from Invitrogen (USA). ReverTra Ace® qPCR RT Master Mix was purchased from TOYOBO (Japan). SYBR Green qPCR master mix was purchased from Bio-Rad (USA). The DNA sequences encoding *APP* rG4 WT/Mut and *APP* full-length WT/Mut containing Myc tag were cloned into the psiCHECK-2 and pEGFPN1 vectors separately by Genewiz (China). Recombinant protein DHX36 was purchased from OriGene Technologies Inc. (USA). RHAU53 peptide was synthesized by SynPeptide Co., Ltd. (China).

### Methods

#### Click reaction between 5′-hexynyl oligonucleotides and azide-modified E3 ligase ligands

The following protocol is for alkyne-modified oligonucleotide and azide-containing compounds in 20 μl of Dimethyl sulfoxide (DMSO) and nuclease-free water reaction mixture [27]. Before the reaction, 1.6 nmol of 5′-hexynyl oligonucleotides (from 1 mM stock in nuclease-free water) was denatured at 95°C for 5 min and cooled for 10 min on ice. Two nanomoles of triethylammonium acetate buffer (from 2 M stock in nuclease-free water, pH 7.0) was added to a final concentration of 0.2 M. Two microliter of DMSO was added and the solution was vortexed. 6.4 nmol of azide compounds stock solution (from 1 mM stock in DMSO) was added to a final concentration of 320 μM, and vortex. Ten nanomoles freshly prepared ascorbic acid (from 10 mM stock in nuclease-free water) and 10 nmol Cu (II)-Tris(benzyltriazolylmethyl) amine [Cu (II)-TBTA], from 10 mM stock in DMSO] were added to the mixture, and vortex briefly. 0.6 μl of DMSO and 5.4 μl of nuclease-free water (∼50:50 ratios in the final reaction mixture) were added to solubilize all the reagents used in the reaction mixture. The solution was degassed by bubbling nitrogen gas for 30 s and tightly sealing it. The click reaction was kept on a thermo shaker at 40°C at 1000 rpm for 6 h. The rG4 conjugate was purified by spin column. The ethanol precipitation protocol [34] was performed for purification of dG4 conjugate (oligonucleotide nucleotide length <20 nt). Twelve percent denaturing polyacrylamide gel was used to analyze click reaction efficiency after staining with SYBR Gold and click efficiency determination was carried out by ImageJ. The synthesized conjugate was confirmed by 4800 Plus MALDI TOF/TOF Analyzer (ABI).

#### Electrophoretic mobility shift assay

All oligonucleotides were heated for 5 min at 75°C and cooled down for 10 min on ice. Different concentrations of RHAU53 peptide (0–2 μM) and FAM-labeled oligonucleotides or G4-compound conjugate (30 nM) were mixed in the Tris–HCl buffer (25 mM Tris–HCl, pH 7.5, 150 mM KCl, and 1 mM MgCl_2_). RHAU53 concentration against FAM_rG4_P_PEG_1_-C_2_ is 0–4 μM. The mixture was incubated at 37°C for 30 min and 5% glycerol was added to each sample. Bound and unbound RNAs were separated on 10% native polyacrylamide gel in 0.5 × Tris/Borate/EDTA (TBE) at 4°C, 150 V for 30 min. The gel was scanned by Amersham TYPHOON scanner at 500 V and quantified by ImageJ. Band intensity was normalized as the fraction of RNA bound (R). For two shift bands, *K*_d_1 was determined from the fraction of first transition band (R1) versus the concentration of RHAU53, and *K*_d_2 was determined from the fraction of second transition band (R2) versus the concentration of RHAU53. The binding curve was fitted by GraphPad Prism, and *K*_d_ is estimated by fitting the equation:


\begin{eqnarray*}R1 &=& \frac{{\left[ {{\mathrm{Bound}}1} \right]}}{{\left[ {{\mathrm{Unbound}}} \right] + \left[ {{\mathrm{Bound}}1} \right]}}\\ \\ R2 &=& \frac{{\left[ {{\mathrm{Bound}}2} \right]}}{{\left[ {{\mathrm{Bound}}1} \right] + \left[ {{\mathrm{Bound}}2} \right]}}\end{eqnarray*}

#### Competitive inhibition of FAM_dG4_A-RHAU53 binding by rG4_A

FAM-labeled dG4_A (30 nM) was mixed with different concentrations of rG4_A (0–2 μM) and a fixed amount of RHAU53 (100 nM) in Tris–HCl buffer (25 mM Tris–HCl, pH 7.5, 150 mM KCl, and 1 mM MgCl_2_). The mixture was incubated at 37°C for 30 min and 5% glycerol was added to each sample. Bound and unbound RNAs were separated on 10% native polyacrylamide gel in 0.5 × TBE at 4°C, 150 V for 30 min. The gel was scanned by Amersham TYPHOON scanner at 500 V and quantified by ImageJ. Band intensity was normalized as the fraction of RNA bound. The inhibition curve of rG4_A on the FAM_dG4_A-RHAU53 complex was fitted by GraphPad Prism using the dose-response inhibition model. IC50 value was calculated using the log[rG4_A] and normalized response.

#### Competitive inhibition of FAM_rG4_A-RHAU53 binding by dG4_A

FAM-labeled rG4_A (30 nM) was mixed with different concentrations of dG4_A (0–2 μM) and a fixed amount of RHAU53 (100 nM) in Tris–HCl buffer (25 mM Tris–HCl, pH 7.5, 150 mM KCl, and 1 mM MgCl_2_). The preparation and analysis method of polyacrylamide gel electrophoresis (PAGE) Gel is the same as that of competitive inhibition of FAM_dG4_A-RHAU53 binding by rG4_A.

#### Microscale thermophoresis

Before the reaction, FAM labeled G4-compound conjugate was heated at 75°C for 5 min and cooled down on the ice for 10 min. DHX36 protein was serially diluted to 16 samples from the highest concentration of 1 μM. FAM-labeled oligonucleotides (40 nM) were added to each sample and incubated in Tris–HCl buffer (25 mM Tris–HCl, pH 7.5, 150 mM KCl, and 1 mM MgCl_2_) at 37°C for 30 min. Binding affinity was carried out on capillary tubes using an microscale thermophoresis (MST) machine (Monolith NT.115). The curve fitting was obtained by NanoTemper Analysis with a *K*_d_ model. The *K*_d_ is estimated by fitting the equation:


\begin{eqnarray*} && f\left( {{c_{ligand}}} \right) = Unbond + \left( {Bound - Unbound} \right) \nonumber\\ &&\,\,\cdot \frac{{{c_{ligand}} + {c_{target}} + {K_d} - \sqrt {{{\left( {{c_{ligand}} + {c_{target}} + {K_d}} \right)}^2} - 4 \cdot {c_{ligand}} \cdot {c_{target}}} }}{{2{c_{target}}}}\end{eqnarray*}

where $f( {{c_{ligand}}} )$ is the *F*_norm_ value at a given ligand concentration ${c_{ligand}}$; $Unbound$ is the *F*_norm_ signal of the target alone; $Bound$ is the *F*_norm_ signal of the complex; *K*_d_ is the dissociation constant or binding affinity; and ${c_{target}}$ is the final concentration of target in the assay.

#### rG4-PROTACs degradation effect on DHX36 by western blot assay

1 × 10^5^ HeLa cells were seeded on a 12-well plate and cultured overnight. rG4-PROTACs or rG4 mut-PROTACs were transfected to cells for 24 h using a Lipofectamine 2000 reagent (Thermo Fisher Scientific). Cells were lysed in RIPA buffer supplemented with a 1 × protease inhibitor cocktail (Thermo Fisher Scientific). Total protein was obtained by centrifuging at 13 000 rpm for 15 min at 4°C. Thirty nanogram cell lysates of each sample were resolved by 8% SDS-PAGE gel at 100 V for 15 min and 120 V for 40 min. The protein blots were transferred to PVDF membrane (Sigma, German) at 100 V for 90 min at 4°C and blocked with 5% non-fat milk for 1 h at room temperature. Then the membrane was incubated overnight with DHX36 primary antibody (1:1000, Invitrogen # PA5-57259) at 4°C. After washing five times with TBST, the membrane was incubated with secondary antibody (1:1000, Proteintec # SA00001-2) for 1h at room temperature and then washed five times with TBST. The membrane was then detected by ChemiDoc Touch Imaging System (Biorad, USA) and analyzed by ImageJ.

#### Immunofluorescent staining

2 × 10^4^ HeLa cells were seeded in a 35 mm diameter confocal dish and cultured overnight. FAM-labeled rG4-PROTACs (3 pmol) or FAM-labelled rG4 mut-PROTACs (3 pmol) were transfected to cells by Lipofectamine® 2000 for 4 h. The cells were fixed with 4% paraformaldehyde for 15 min and washed three times with nuclease-free PBS. Then the cells were permeabilized with 1 ml 0.3% Triton^@^ X-100 at room temperature for 20 min. After washing three times with nuclease-free PBS, incubate cells with 1% BSA in PBS for 30 min to block unspecific binding of the antibodies. Then incubate cells in the diluted DHX36 antibody (1:500, Proteintec #13159-1-AP) in 1% BSA overnight at 4°C. Wash the cells three times with PBS and incubate cells with Alexa Fluor™ 647 secondary antibody (1:500, Thermo Fisher Scientific # A-21245) in 1% BSA for 1 h at room temperature in the dark. After washing three times with PBS, HeLa cells were stained with DAPI for 15 min. Cell imaging was performed with a confocal microscope (Leica TCS SPE).

#### DHX36 protein unwinding assay on *APP* rG4

The unwinding assay was performed as previously reported [11]. Briefly, an annealing buffer (60 mM HEPES, pH 7.5, 6 mM KCl, 2 mM MgCl_2_, and 1 mM DTT) was prepared before the reaction, Lane 1 (Fig. 4B): The positive control (Marker band) was obtained by incubating 100 nM FAM-labelled *APP* rG4 WT with 25 nM Trap RNA (Supplementary Table S1) in annealing buffer at 95°C for 5 min and then programmed freezing to 21°C at 0.1°C/min [35]. Five percent glycerol was added to the sample and kept on ice before loading on gel. Lane 2: 100 nM *APP* rG4 WT was incubated in an annealing buffer at 95°C for 5 min and then decreased to 21°C at 0.1°C/min. Lane 3: 100 nM *APP* rG4 WT was incubated with 25 nM Trap RNA in an annealing buffer but did not undergo an annealing procedure. Lane 4: 20 μl reaction mixture containing 100 nM *APP* rG4 WT was incubated at 37°C for 10 min with 76 nM DHX36 protein. 25 nM Trap RNA was added to the mixture without ATP at 37°C for 15 min. Lane 5: 20 μl reaction mixture containing 100 nM *APP* rG4 WT was incubated at 37°C for 10 min with 76 nM DHX36 protein. 25 nM Trap RNA and 2 mM AMP-PNP (Sigma–Aldrich, USA) were added to the mixture at 37°C for 15 min. Lane 6: 20 μl reaction mixture containing 100 nM *APP* rG4 WT was incubated at 37°C for 10 min with 76 nM DHX36 protein. 25 nM Trap RNA and 2 mM ATP (Thermo Fisher Scientific, USA) were added to the mixture at 37°C for 15 min. Lane 4–6 samples were treated with proteinase K (Thermo Fisher Scientific, USA) at 37°C for another 15 min to degrade DHX36 protein. All prepared samples were separated on 15% native polyacrylamide gel in Tris–HCl buffer (25 mM Tris–HCl, pH 7.5, 50 mM KCl, and 1 mM MgCl_2_) at 4°C, 35 mA for 40 min. The gel was scanned by the Amersham TYPHOON scanner at 500 V.

#### Dual-luciferase reporter gene assay

The DNA sequences encoding *APP* rG4 WT and *APP* rG4 Mut (Supplementary Table S1) were introduced into the 5′ UTR of the Renilla luciferase gene in the psiCHECK-2 vector, respectively, by Genewiz (China). 2 × 10^4^ HeLa cells were seeded on 96-well black-wall plates (SPL, Korea). Ten nanogram WT or Mut plasmids were co-transfected with rG4_A and rG4 mut_A (0, 50, 100, and 200 nM) separately to cells by Lipofectamine® 2000 and incubated for 48 h. Luciferase activity was determined by Molecular Devices SpectraMax ID5. The Renilla activity was normalized to Firefly activity for data analysis.

#### Quantitative real-time PCR assay for dual luciferase reporter gene

The Co-transfection method was the same as the Dual-luciferase reporter gene assay. Total RNA in 1 × 10^5^ HeLa cells was extracted by RNeasy Plus Mini Kit (Qiagen, Germany). One hundred nanogram total RNA was reverse transcribed by the RT-Ace (TOYOBO, Japan) using a random primer. For PCR amplification procedures, the primers of reporter genes (Supplementary Table S1) and cDNA were mixed with the SYBR Green qPCR master mix (Bio-Rad). The RT-PCR was performed by a CFX96 TouchTM Real-Time PCR Detection System (Bio-Rad).

#### rG4-PROTACs effect on native APP protein expression

The DNA sequences encoding the *APP* full-length coding region, the Myc tag, and the *APP* rG4 WT or rG4 Mut (Supplementary Table S1) were introduced into the pEGFPN1 vector (coined to *APP* WT or Mut native plasmid), respectively, by Genewiz (China). 1 × 10^5^ HEK 293T cells were seeded on a 24-well plate and cultured overnight. Five hundred nanogram *APP* native WT or Mut plasmids were co-transfected with rG4_A and rG4 mut_A (0, 25, 50, and 100 nM) separately to cells by Lipofectamine® 2000 and incubated for 24 h. DHX36, APP, and Myc protein expression were analyzed by western blot, which was the same as rG4-PROTACs degradation effect on DHX36 by western blot assay. APP primary antibody (1:1000, Merck # MABN380) and Myc primary antibody (1:1000, Thermo Fisher Scientific # MA1-980) were used to incubate the PVDF membrane.

#### rG4-PROTACs effect on endogenous APP protein expression

1 × 10^5^ HeLa cells were seeded on a 12-well plate and cultured overnight. rG4-PROTACs (0, 15.6, 31.2, 62.5, 125, 250, and 500 nM) were transfected to cells for 24 h using a Lipofectamine 2000 reagent (Thermo Fisher Scientific). Endogenous APP protein expression was analyzed by western blot which was the same as rG4-PROTACs degradation effect on DHX36 by western blot assay. APP primary antibody (1:1000, Merck # MABN380) were used to incubate the PVDF membrane.

#### rG4-PROTACs effect on Gnai2 protein expression

Mouse C2C12 myoblast cells (CRL-1772) were cultured in DMEM medium supplemented with 10% fetal bovine serum and 1% penicillin/streptomycin (complete media) at 37°C in 5% CO_2_. 5 × 10^4^ C2C12 cells were seeded on a 24-well plate and cultured overnight. rG4-PROTACs or rG4 mut-PROTACs (0, 50, 100, 150, and 200 nM) were transfected to cells for 24 h using a Lipofectamine 3000 reagent (Thermo Fisher Scientific). DHX36 and *Gnai2* protein expression were analyzed by western blot, which was the same as rG4-PROTACs degradation effect on DHX36 by western blot assay. *Gnai2* primary antibody (1:5000, Abcam # ab157204) was used in this assay.

#### Proteomics analysis

Total protein was extracted from HeLa cells following a systematic procedure. Briefly, cells were collected and suspended in RIPA lysis buffer (Beyotime) containing a protease inhibitor cocktail (Fdbio science) and phosphatase inhibitors (Fdbio science). The suspension was incubated on ice for 15 min, sonicated in an ice bath for 18 s, and then centrifuged at 12 000 rpm for 15 min at 4°C. Protein concentration was determined using the BCA protein assay (Thermo). A total of 300 μg of protein was precipitated overnight at −20°C by adding 800 μl of pre-cooled acetone. The precipitated proteins were washed with pre-cooled acetone and resuspended in 50 μl of UA buffer (8 M urea, 0.1 M Tris, pH 8.5) for digestion. Next, 2 μl of 50 mM DTT was added, and the samples were incubated at 37°C for 1 h. Following this, 13 μl of 50 mM IAA was added, and the samples were incubated in the dark for 30 min. Subsequently, 3.75 μg of trypsin (Thermo) was added along with 535 μl of 50 mM NH_4_HCO_3_, and the mixture was incubated overnight at 37°C. The resulting peptides were desalted using a C18 column. After vacuum centrifugation to dryness, the peptides were dissolved in 0.1% formic acid for mass spectrometry analysis. Differential protein expression levels were analyzed under the following criteria: a ratio >2 or <0.5 and a *P* value <0.05. The analysis focused on differential protein expression based on abundance ratios and significance thresholds. Results were exported for Gene Ontology (GO) analysis, enabling exploration of the biological functions associated with the differentially expressed proteins.

#### EdU cell proliferation assay

C2C12 myoblast cells and satellite cells (SCs) were used in this assay. SCs were isolated from skeletal muscle tissues and cultured in Ham’s F10 medium supplemented with 20% fetal bovine serum and bFGF (0.025 μg/ml) (growth medium) as previously described [20]. EdU staining was followed by the manufacturer’s protocols (Thermo Fisher Scientific, C10086). 5 × 10^4^ C2C12 or SCs cells were seeded on coverslips and cultured overnight. rG4-PROTACs or rG4 mut-PROTACs (200 nM) were transfected to cells for 24 h using a Lipofectamine 3000 reagent (Thermo Fisher Scientific). Dilute 10 mM EdU stock solution by complete medium to 20 μM. Transfer coverslips into the 6-well plate. Then add an equal volume of the 20 μM EdU solution to obtain a 10 μM final solution and incubate for 3 h. Remove the medium and fix cells by 1ml 3.7% PFA for 15 min at room temperature. Wash cells two times with 3% BSA in PBS. Add 1ml 0.5% Triton^@^ X-100 for 20 min. Wash cells two times with 3% BSA in PBS. Add freshly prepared Alexa Fluor® 594 azide reaction cocktail to each well and incubate away from light for 30 min. Wash cells two times with 3% BSA in PBS, cells were stained with DAPI for 15 min. Cell imaging was performed with a confocal microscope (Leica TCS SPE).

## Results

### Design and synthesis of rG4-PROTACs targeting DHX36

**Figure 1.**
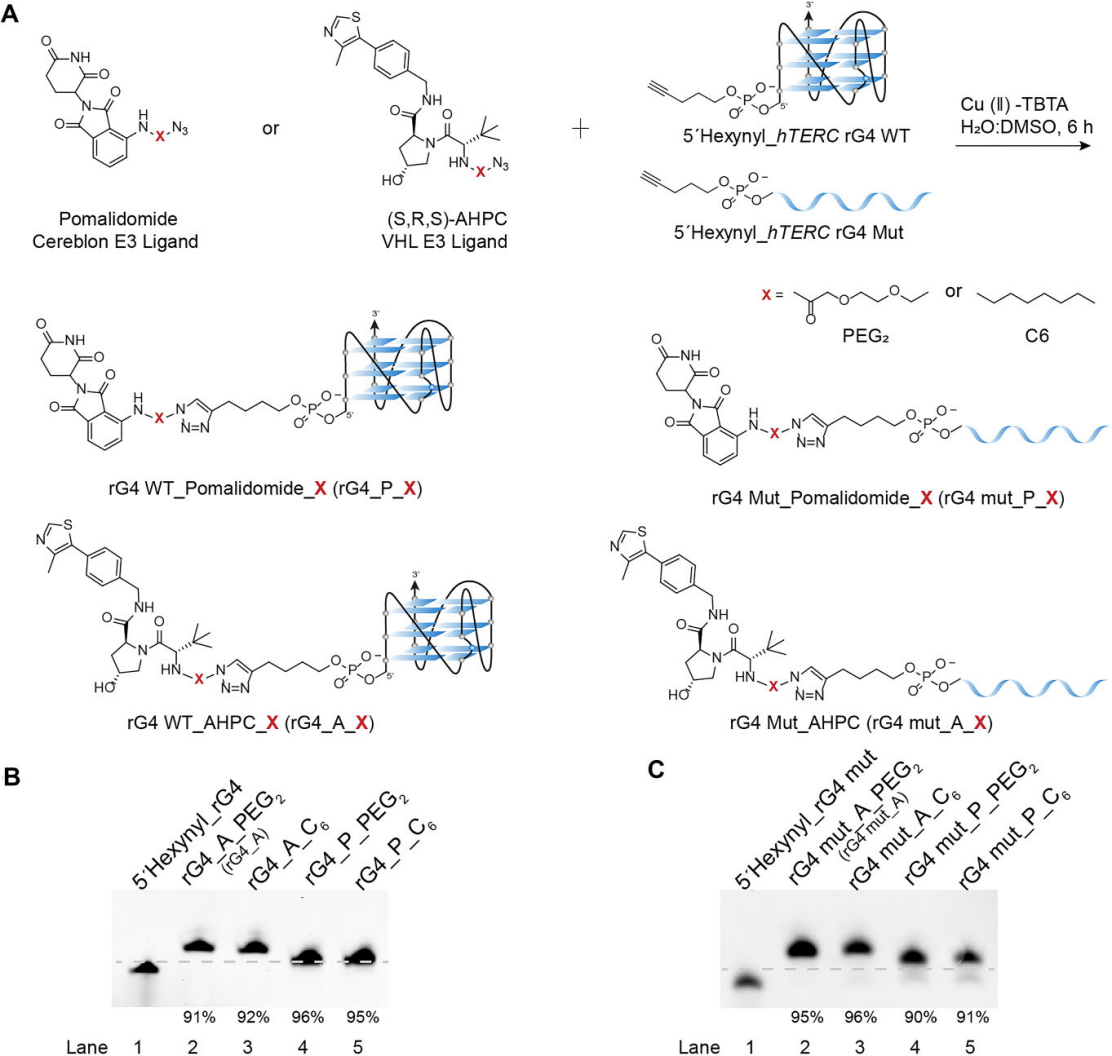
Rational design and preparation of rG4-PROTACs by click chemistry. (**A**) Schematic representation of the synthesis of rG4-PROTACs between azide-modified E3 ligase ligands and alkyne-modified rG4 oligos. (S, R, S)-AHPC and Pomalidomide are E3 ligase ligand-linker conjugates. Alkyne was introduced to the 5′ end of *hTERC* rG4 WT or *hTERC* rG4 Mut sequences (Supplementary Table S1). The E3 ligase recruiter compounds were separately conjugated onto 5′Hexynyl_ *hTERC* rG4 WT and 5′Hexynyl_ *hTERC* rG4 Mut by click reaction for 6 h to generate rG4-PROTACs (rG4_A, rG4_A_C_6_, rG4_P_PEG_2_, rG4_P_C_6_, rG4 mut_A, rG4 mut_A_C_6_, rG4 mut_P_PEG_2_, and rG4 mut_P_C_6_). X: PEG_2_ or C_6_. (**B**) Confirmation of rG4-PROTACs conjugates. (**C**) Confirmation of rG4 mut-PROTACs conjugates. The click reaction products were subjected to 12% denaturing PAGE at 300 V for 30 min. All these conjugates achieved >90% of reaction yields. Dotted lines are used to distinguish bands.

RHAU-specific motif-containing 53 amino acids (RHAU53), the truncated DHX36 fragment, is the most important core protein domain required for rG4-RHAU interaction [16, 36]. We previously reported that rG4 located in the human telomerase RNA component *(hTERC)* (5′-GGGUUGCGGAGGGUGGGCCU-3′) could strongly bind to RHAU53 with nanomolar affinity [37]. Accordingly, we initially selected *hTERC* rG4 (*hTERC* rG4 WT) as the warhead targeting DHX36 and designed the control sequence (*hTERC* rG4 Mut) by mutating several Gs into As to disrupt rG4 formation (Supplementary Table S1). Meanwhile, we employed two E3 recruiting molecules AHPC (von Hippel–Lindau, VHL ligands, referred to as A) [38] and Pomalidomide (cereblon ligands, referred to as P) [39] with diverse linkers to connect with the rG4 motif (Synthetic schemes S1 and S2). Click reaction has been widely used to covalently modify nucleic acid with fast, efficient, selective manner [40]. We therefore attempted to use copper(I)-catalyzed azide-alkyne cycloaddition (CuAAC) [41] to connect rG4 and E3 recruiters. Specifically, alkyne was attached to the 5′ end of rG4 (Supplementary Table S1) and azide was introduced to derivatives of AHPC and Pomalidomide (Fig. 1A). To improve the efficiency of CuAAC, we optimized the reaction conditions including reaction time and the molar ratio of 5′-hexynyl oligonucleotides to azide-modified E3 ligase ligands, eventually enhancing the click efficiency to >90% (Supplementary Fig. S1). We successfully synthesized the rG4-PROTACs (rG4_A_linker, rG4 mut_A_linker, rG4_P_linker, and rG4 mut_P_linker) using 5′ hexynyl-modified RNA oligomers and azide-modified compounds via CuAAC click reactions (Fig. 1A and Supplementary Fig. S2A). After clean-up by Zymo-Spin IC Columns, the click efficiency was monitored by denaturing PAGE and these conjugates achieved click efficiency ranged from 90% to 96% (Fig. 1B and C; Supplementary Fig. S2B), suggesting an almost quantitative reaction. All conjugates were further confirmed by Mass spectrometry (Supplementary Table S2).

### rG4-PROTACs exhibits stronger binding affinity to RHAU53 and DHX36 than dG4-PROTACs

**Figure 2.**
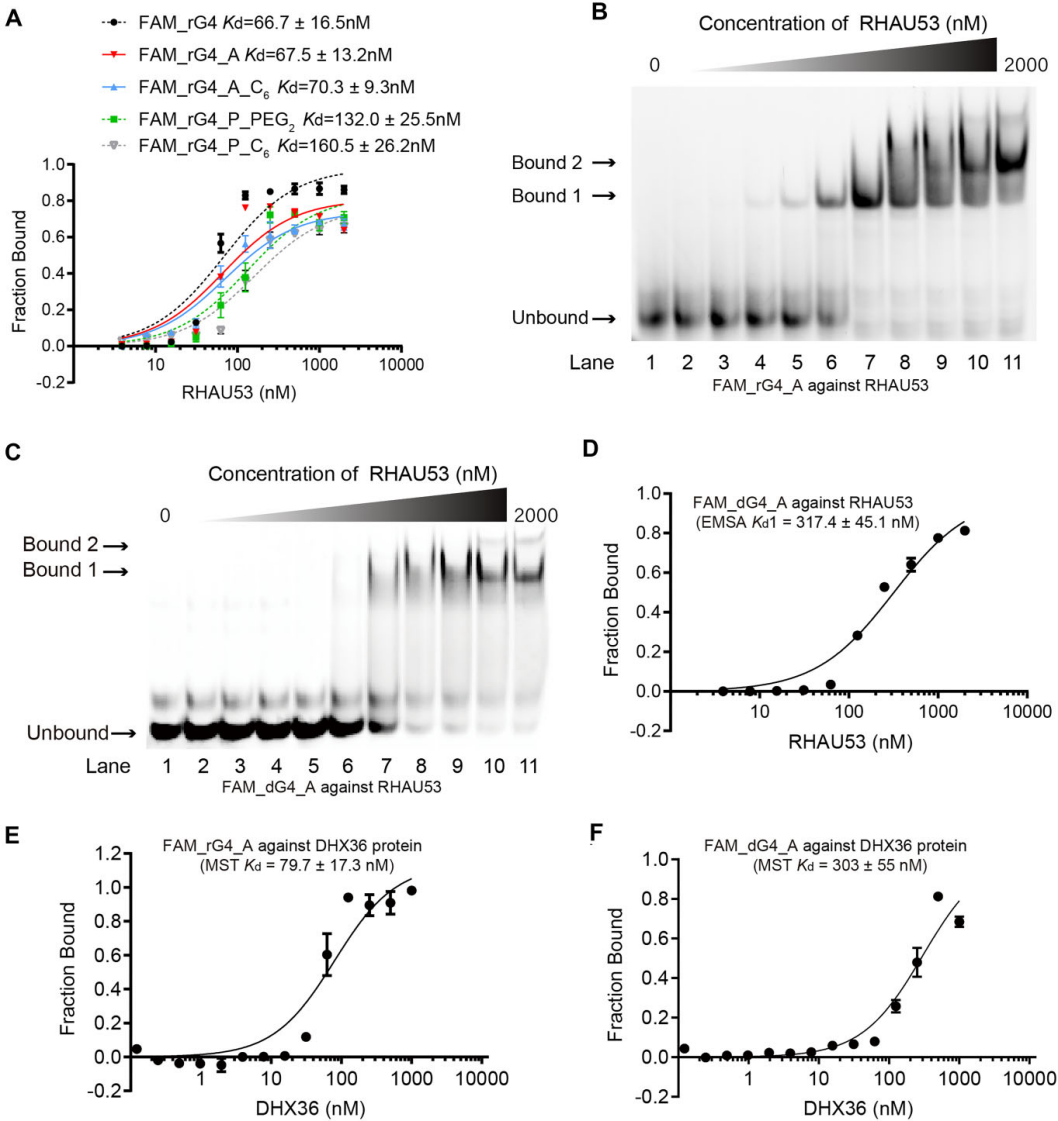
rG4_PROTACs demonstrate stronger molecular recognition toward RHAU53 and DHX36 than dG4_PROTACs. (**A**) Binding curves of FAM_rG4 WT_Protacs against RHAU53 from the first shift bound (Bound 1). For 2 transitions, *K*_d_1 was determined from the fraction of first transition band (R1) versus the concentration of RHAU53 and *K*_d_2 was determined from the fraction of second transition band (R2) versus the concentration of RHAU53. (**B**) Native gel of the binding between FAM_rG4_A and RHAU53 detected by EMSA. The free rG4 band was completely shifted to bound 1 at lane 7, and bound 2 appeared at lane 9. (**C**) EMSA result of the binding between FAM_dG4_A and RHAU53. The free dG4 band was completely shifted to bound 1 at lane 10, and the bound 2 band appeared at lane 10. (**D**) Binding curves of FAM_dG4_A against RHAU53 from the first shift bound (Bound 1) and the *K*_d_1 was calculated to be 317.4 ± 45.1 nM. (**E**) MST binding curve between FAM_rG4_A and DHX36 protein and the *K*_d_ was found to be 79.7 ± 17.3 nM, which verifies the binding interaction between FAM_rG4_A and DHX36. (**F**) MST binding curve between FAM_dG4_A and DHX36 protein and the *K*_d_ was found to be 303 ± 55 nM, which showed a relatively weaker binding interaction between FAM_dG4_A and DHX36. The data were obtained from three biological replicates with the standard deviation as an error bar.

To evaluate the target recognition ability of the rG4-PROTACs, the 3′ end of 5′Hexynyl_*hTERC* rG4 WT or Mut sequences (Supplementary Table S1) was labeled with Fluorescein (FAM) and conjugated to the E3 ligase recruiters, hereafter referred to as FAM_rG4_A_linker, FAM_rG4 mut_A_linker, FAM_rG4_P_linker, and FAM_rG4 mut_P_linker (Supplementary Fig. S3A and B; Supplementary Table S2). We performed the binding assay of rG4-PROTACs to RHAU53 peptide by electrophoretic mobility shift assay (EMSA). Firstly, FAM_rG4_P_PEG_2_, FAM_rG4_P_C_6_, and FAM_rG4_P_PEG_1_-C_2_ were prepared to determine the effect of the linker types on the binding affinity. Among all, FAM_rG4_P_PEG_2_ exhibited a higher binding affinity against *hTERC* rG4 WT (Supplementary Fig. S4A and Fig. 2A; *K*_d_ value 132.0 ± 25.5 nM) than that of the FAM_rG4_P_C_6_ (Supplementary Fig. S4B and Fig. 2A; *K*_d_ value 160.5 ± 26.2 nM) and FAM_rG4_P_PEG_1_-C_2_ (Supplementary Fig. S4C and D; *K*_d_ value 173.5 ± 35.3 nM). As such, PEG_2_ and C_6_ were more suitable as the linkers between FAM_rG4 and E3 ligase ligands. Secondly, we determined the effect of E3 ligase recruiters on the binding affinity. PEG_2_ and C_6_ were attached to AHPC, and then covalently coupled with FAM_rG4 separately to produce FAM_rG4_A_PEG_2_ and FAM_rG4_A_C_6_. EMSA results showed that replacing pomalidomide with AHPC can improve the binding affinity of rG4_PROTACs conjugate to its target by 1.8 times (Supplementary Fig. S4E and Fig. 2A; *K*_d_ = 70.3 ± 9.3 nM) and 1.9 times (Fig. 2A and B; *K*_d_ = 67.5 ± 13.2 nM). Thirdly, the *K*_d_ of the original FAM_rG4 to RHAU53 was found to be 66.7 ± 16.5 nM (Supplementary Fig. S4F and Fig. 2A), which indicates that FAM_rG4_A_PEG_2_ binding ability to RHAU53 is not interfered by AHPC-PEG_2_. We then used full-length DHX36 protein to further confirm the recognition and the *K*_d_ (79.7 ± 17.3 nM) is consistent with the EMSA result, which suggests the strong interaction between FAM_rG4_A and DHX36 (Fig. 2E). Overall, we have optimized the E3 ligase ligands and linker for the rG4_PROTACs, and FAM_rG4_A_PEG_2_ (FAM_rG4_A) was chosen for downstream experiments.

Recently, alkyne functionalized T95-2T dG4 and mutant sequences (Supplementary Table S1) have been connected to E3 ligase-binding small molecules (dG4-PROTACs) to showcase potent degradation of RHAU by the proteasomal complex [33]. To compare the binding affinity of rG4-PROTACs with dG4-PROTACs side-by-side, we also synthesized FAM_dG4_A and FAM_dG4 mut_A conjugates following the same click reaction method as rG4-PROTACs (Supplementary Fig. S3C). Mass spectrometry validated the formation of the conjugated products (Supplementary Table S2). Notably, the *K*_d_ of FAM_dG4_A to RHAU53 was found to be 317.4 ± 45.1 nM (Fig. 2C and D), which is ∼4.7 times higher than FAM_rG4_A (Fig. 2A and B). Also, the interaction of FAM_dG4_A to DHX36 by MST was shown nearly 3.8 times higher (*K*_d_= 303 ± 55 nM) than that of FAM_rG4_A (Fig. 2E and F), indicating a relatively weaker interaction with DHX36. Furthermore, to investigate the binding specificity of G4-PROTACs, we conducted the EMSA assay using the non-G4 motif (FAM_rG4 mut_A and FAM_dG4 mut_A, Supplementary Table S1), and no binding was observed, suggesting the targeting of G4-PROTACs to DHX36 is depending on G4 formation (Supplementary Fig. S4G and H). To further confirm the strong affinity of rG4-PROTACs to DHX36, we performed a competition assay between FAM_rG4_A and FAM_dG4_A against RHAU53. With an increasing concentration of rG4_A, the FAM_dG4_A–RHAU53 complex (upper bands) diminishes while the unbind dG4 becomes darker (lower bands). This suggests the interaction of FAM_dG4_A and RHAU53 is inhibited competitively by rG4_A (Supplementary Fig. S5A and B). However, the FAM_rG4_A–RHAU53 complex (upper bands) slightly diminishes with an increasing concentration of dG4_A, indicating FAM_rG4_A-RHAU53 complex can’t be completely displaced by dG4_A (Supplementary Fig. S5C and D). Overall, rG4-PROTACs showed ∼4-fold improvement in binding affinity to DHX36 as compared to dG4-PROTACs, indicating stronger molecular recognition for downstream applications.

### rG4-PROTACs induce DHX36 degradation in a ubiquitination-dependent manner and work more efficiently than dG4-PROTACs and DHX36 siRNAs in cells

**Figure 3.**
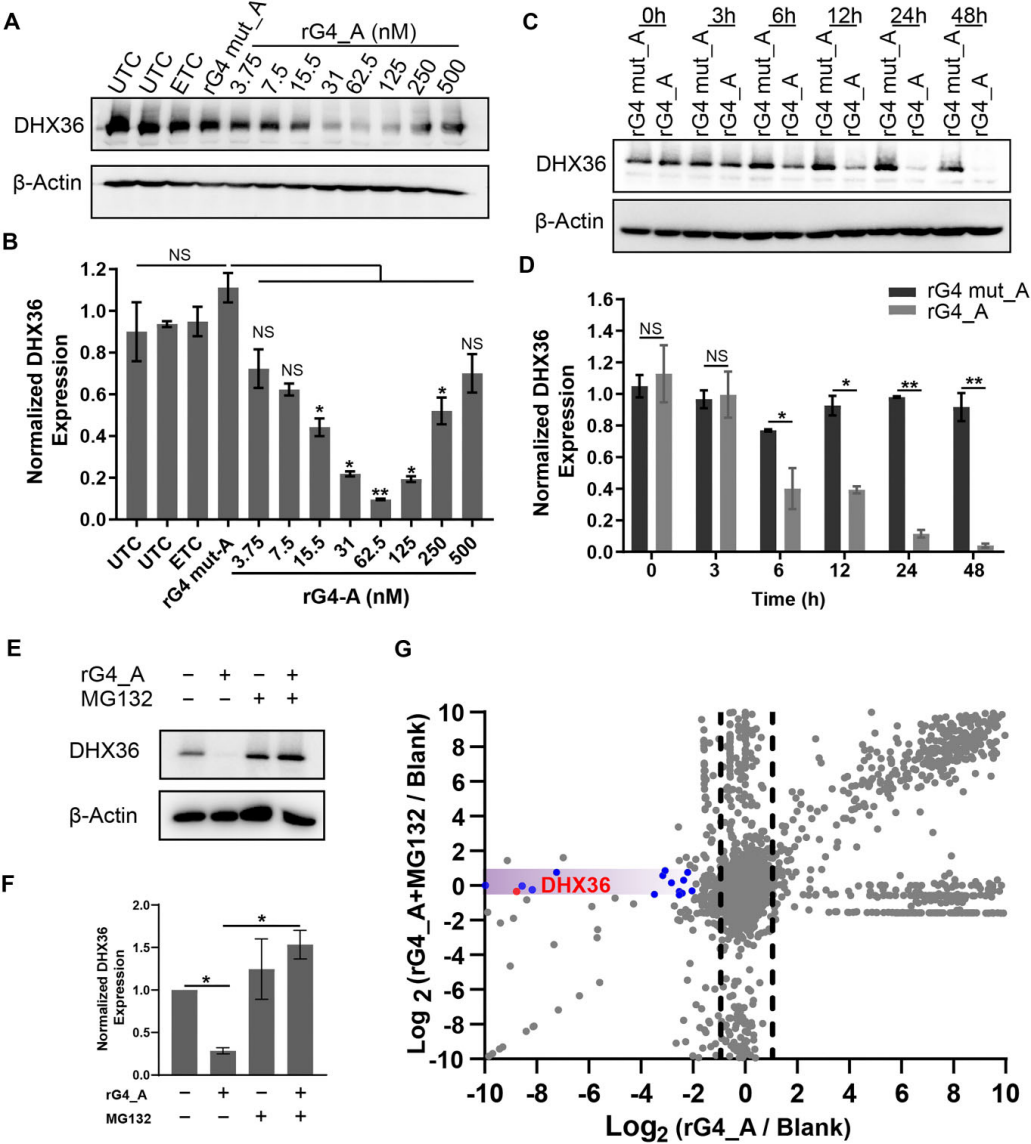
rG4_PROTACs degrade DHX36 protein in a proteasome-dependent manner in different human cells. (**A**) rG4_A degrades DHX36 in a dose-dependent manner. HeLa cells were treated with different concentrations of rG4_A and 250 nM rG4 mut_A for 24 h, and the cells were harvested for western blotting analysis of DHX36. UTC: Untreated cells; and ETC: empty transfection control. (**B**) Quantitative analysis of western blot from panel (A). (**C**) Degradation effect of rG4_A (50 nM) and rG4 mut_A (50 nM) on DHX36 0–48 h post-transfection. (**D**) Quantitative analysis of western blot results at different time points. (**E**) MG132 blocked rG4_A induced DHX36 protein reduction. HeLa cells were treated with rG4_A (50 nM) with or without MG132 (1 μg/ml), and the cells were harvested for western blotting analysis of DHX36. (**F**) Quantification diagram of western blot from panel (E). (**G**) Proteomic analysis of rG4_A selectively degraded DHX36 through the proteasome. Hela cells were treated with 50 nM rG4_A, or rG4 mut_A plus MG132 for 24 h. Lysates were subjected to mass spec-based proteomics analysis. The volcano plot shows protein abundance (Log_2_) as a function of significance level. The significantly downregulated proteins through proteasome were found in the purple zone [−0.41< [Log_2_ (rG4_A + MG132 / Blank) ≈ 0] <0.32. Nonaxial vertical lines mark log_2_Fold Change >1 or <−1 significance threshold. Log_2_fold change was obtained from three independent experiments of Log_2_ (rG4_A / Blank) or Log_2_ (rG4_A + MG132 / Blank). *P < 0.05 and **P < 0.01. Normalized DHX36 expression was obtained from biological triplicates with the standard deviation as an error bar.

To verify if rG4-PROTACs can enter cells, we performed confocal microscopy and the results suggested that FAM-labeled rG4-PROTACs (FAM_rG4_A and FAM_rG4 mut_A) could be transfected successfully into Hela cells (Supplementary Fig. S6A). Meanwhile, Cells treated with FAM_rG4_A exhibited strong fluorescent foci colocalized with endogenous DHX36, whereas little to no colocalized foci were observed in cells with FAM_rG4 mut_A transfection (Supplementary Fig. S6B), indicating that only the transfected rG4 motif degrader could target and interact with DHX36 protein. To characterize the degradation profile of rG4-PROTACs, we transfected varying concentrations of rG4_A in Hela cells. The western blot result showed that the protein levels of DHX36 were significantly reduced by increasing concentrations of rG4_A, the highest DHX36 degradation (over 90%) was observed at 62.5 nM (Fig. 3A and B). Above 62.5 nM, protein degradation was attenuated with the increased dose of rG4-A (Fig. 3A and B), which corroborated those of previously reported PROTACs [33, 42]. The weaker effects at higher concentrations beyond 62.5 nM could be attributed to the cellular hook effect [43], where the DHX36-rG4_A / rG4_A-E3 ligase binary interactions efficiently compete with DHX36-rG4_A-E3 ligase ternary interactions. As negative controls, neither untreated control (UTC), empty transfection control (ETC), nor rG4 mutant variant (rG4 mut_A) had a significant effect on DXH36 protein levels. In addition, the time dependency of rG4-A on DHX36 showed that DHX36 reduction exceeded 50% within 6 h of cells treated with 50 nM rG4_A, and maximal degradation was achieved at 95% at 48 h (Fig. 3C,D). We also performed similarly with other rG4_PROTACs (50 nM, 24 h) and found that rG4_A and rG4_P with diverse linkers could degrade >80% DHX36 protein. Notably, rG4_A with PEG_2_ demonstrated higher efficiencies in inducing DHX36 degradation (>85% degradation for rG4_A, Supplementary Fig. S7A and B). While, treatment cells with rG4_P_PEG_1__C_2_ (50 nM, 24 h) resulted in weaker effects (42% degradation for rG4_P_PEG_1__C_2_ versus 85% degradation for rG4_A) in Hela cells (Supplementary Fig. S7C and D). We further monitored the concentration-dependent degradation of DHX36 protein by rG4_P_PEG_1__C_2_. The highest DHX36 degradation (∼50%) was observed at 125 to 500 nM after rG4_P_PEG_1__C_2_ treatment for 24h (Supplementary Fig. S7E and F). These data suggested that rG4-A worked most efficiently and was chosen for the subsequent experiments. Furthermore, rG4_A-induced degradation of DHX36 was blocked by the proteasome inhibitor MG132 (1 μM), indicating that rG4 PROTACs-mediated DHX36 degradation in a proteosome-dependent manner (Fig. 3E and F). Similarly, transfection of rG4_A (50 nM) to HEK293T and MCF-7 cell lines also led to ∼90% proteasomal degradation of DHX36 protein at the 24 h time point (Supplementary Fig. S8A and B), supporting that rG4-PROTACs can be readily applied to degrade DHX36 protein efficiently in different human cell lines.

To explore the effects of rG4-PROTACs on the whole proteome, Hela cells were treated with blank (empty transfection control), rG4 mut_A, rG4_A, or rG4_A plus MG132, and a proteomic analysis was employed. We speculate that MG132 could block DHX36’s decrease and rG4-containing gene changes. Thus, the gene modulation activity directly caused by DHX36 degradation was determined. Among 8000 quantified proteins, 44 down-regulated proteins were detected and DHX36 ranked as one of the most significant degradation proteins after rG4_A treatment. At the same time, no obvious difference was observed in the rG4 mut_A treatment group (Supplementary Fig. S9A and B). Next, we analyzed some other G4BPs that have been proven to interact with rG4 and no significant changes were found, such as NCL (nucleolin), DHX9, hnRNP (the heterogeneous nuclear ribonucleoproteins), SRSF (serine/arginine-rich splicing factors), or FMR1 (Fragile X-related Protein1) (Supplementary Fig. S9A) [9]. It was worth noting that MG132 can abolish 34 significant declined proteins including DHX36 and none of them are known G4BP (purple zone, Fig. 3G, Supplementary Fig. S9C). Of the first 15 significantly down-regulated proteins, no classical RBPs were found except DHX36 (blue dots in purple zone, Fig. 3G, Supplementary Table S3), further proving that rG4_A routed only a few proteins into proteasome for degradation and the targeting effect of rG4_A to G4BP was highly specific. Importantly, we characterized the association between rG4 enrichment and DHX36 degradation from G4 atlas (*https://www.g4atlas.org/*). By further alignment with rG4 candidates from rG4-seq [7], we found that rG4s were significantly enriched (41%) in down-regulated proteins blocked by MG132, while only 9% reduced genes that MG132 could not block contain rG4s sites (Supplementary Fig. S9D), suggesting the direct regulating roles of DHX36 degradation on gene expression via rG4 motif, which is consistent with the negative function of the DHX36 as a helicase in modulating gene activity [44]. Furthermore, GO [45] analysis revealed that the downregulated targets of rG4_A are significantly associated with protein transport and protein binding function (Supplementary Fig. S10). Therefore, rG4_A triggered DHX36 degradation involving proteasome, which could not induce significant degradation of other known G4BPs.

We also tested the DHX36 degradation effect with dG4_A using the same conditions. Notably, the dose-dependent degradation of DHX36 by rG4_A showed a hook effect [43] (Supplementary Fig. S11A and B), while DHX36 protein expression was not significantly inhibited with the increase of dG4_A concentration (Supplementary Fig. S11C and D). These data, which are in good agreement with those obtained with EMSA results (Fig. 2), demonstrated that the targeting effect and degradation efficiency of dG4-PROTACs were relatively weaker than rG4-PROTACs. We further compared the effect of rG4-PROTACs with traditional DHX36 gene-silencing siRNA that triggers the RNAi mechanism. Two designed siRNAs, referred to as siRNA#1 and siRNA#2 (Supplementary Table S1), were tested for their effectiveness. A low concentration of siRNA#1 (<100 nM) resulted in a 50% reduction of DHX36 protein levels over 24 h. In comparison, siRNA#2 achieved the same level of knockdown, but at a higher concentration of 500 nM (Supplementary Fig. S12A and B). Consequently, siRNA#1 and verified siRNA#3 [46] were selected to investigate the time and concentration-dependent effects on DHX36 degradation. When comparing the treated samples to the control siRNA (siNC, Supplementary Table S1), no significant degradation of DHX36 was observed between 6 and 12 h of treatment (Supplementary Fig. S12C and D). It was not until 24 h post-treatment that a 50% decline in DHX36 protein levels was noted, while a maximal silencing effect of over 85% was reached at 48 h (Supplementary Fig. S12E and F). In contrast, siRNA#3 did not significantly reduce DHX36 protein levels before 24 h, achieving only a 50% knockdown by 48 h (Supplementary Fig. S12G–J). Accordingly, compared to the most effective siRNA#1, rG4-PROTACs showed the effect at a much shorter time (>50% reduction was achieved at the time point 6 h and nearly complete depletion of DHX36 was observed in 24 h) (Fig. 3C and D). Together, these findings support the view that rG4 motifs could be harnessed to construct DHX36 targeting degrader, induce ubiquitylation of DHX36, and its effective degradation by the ubiquitin–proteasome system.

### Applications of rG4-PROTACs in controlling *APP* gene and native protein expression

**Figure 4.**
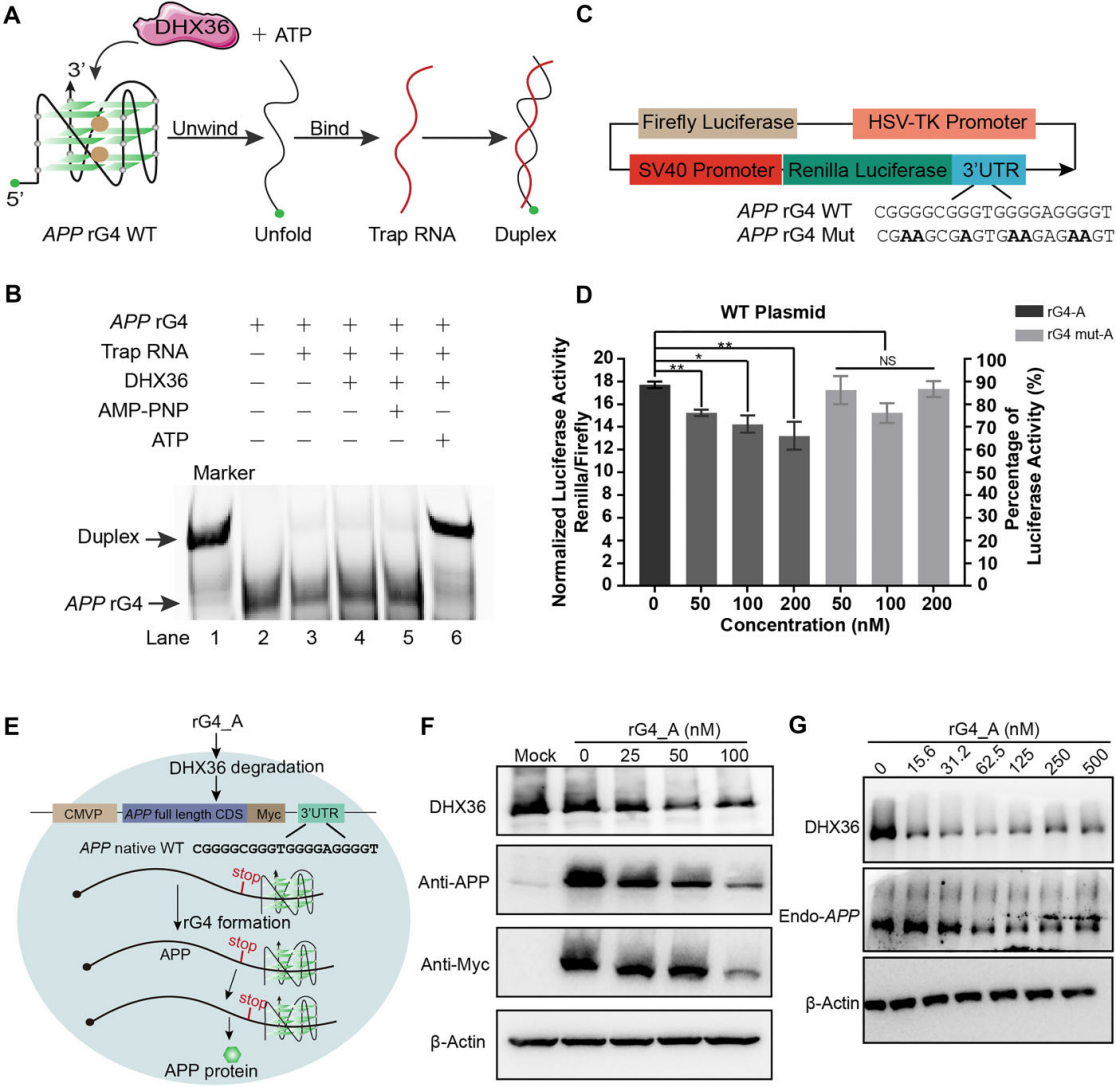
rG4_A negatively regulates *APP* gene and native protein expression in Hela cells. (**A**) Scheme of the functional mechanism of ATP-dependent DHX36 unwinding activity on *APP* 3′ UTR rG4 WT *in vitro*. (**B**) Native gel profile of DHX36 unwinding activity. Lane 1: *APP* rG4 WT and Trap RNA were thermally denatured and hybridized to form a duplex, which exhibits a shift band in the upper position compared with the free *APP* rG4 band. Lanes 2–5: negative controls in which the duplex cannot be formed in the absence of either DHX36 or ATP (lane 2, lane 3, and lane 4), or replacing ATP with AMP-PNP, a non-hydrolyzable analog of ATP (lane 5). Lane 6: after the addition of ATP, DHX36 bounds and unfolds the *APP* rG4 motif. The fully complementary sequence, Trap RNA, will hybridize with the unfolded rG4 motif to form a duplex. (**C**) Schematic illustration of dual luciferase reporter plasmids. The *APP* rG4 WT or *APP* rG4 Mut DNA sequence was inserted into the 3′ UTR of Renilla luciferase. (**D**) Normalized luciferase activity on *APP* rG4 WT plasmid. The luciferase activity of *the APP* rG4 WT construct group (the first black columns) was 100%, and the luciferase signals of all other groups were normalized by that of the *APP* rG4 WT construct group. (**E**) Scheme of translation inhibition of rG4_PROTACs on *APP* native WT plasmid. After co-transfection of the *APP* rG4-containing plasmid and rG4_A for 24 h, rG4 motif-mediated APP protein expression was reduced. (**F**) Western blot analysis of *APP* protein expression after treatment with different concentrations of rG4_A (0−100 nM). *APP* protein decreased along with DHX36 degradation. Anti-Myc tag and anti-APP antibodies were used to detect *APP* protein, respectively. β-Actin: internal loading control. Mock: empty transfection control. (**G**) Relative APP mRNA expression levels on endogenous APP expression. Western blot analysis was conducted to assess the expression of endogenous APP protein following treatment with various concentrations of rG4_A (0, 15.6, 31.2, 62.5, 125, 250, and 500 nM). The results showed that endogenous APP protein levels decreased in parallel with the degradation of DHX36. Normalized luciferase activities are obtained from biological triplicates with the standard deviation as an error bar. *P < 0.05, **P < 0.01, NS: not significant.

Recently, we reported that the rG4 motif existed in the 3′ untranslated region (3′ UTR) of *APP* mRNA by biochemical approaches [47]. The MST result showed that the binding affinity of *APP* rG4 to DHX36 is strong (*K*_d_ = 19.7 ± 2.3 nM) (Supplementary Fig. S13). We speculate that DHX36 may have a helicase effect on the *APP* 3′ UTR rG4, and that rG4-PROTACs can induce the formation of *APP* rG4, leading to the inhibition of its gene expression by promoting the degradation of DHX36. To test this hypothesis, we synthesized FAM-labeled *APP* rG4 WT oligomers containing the polyA tail and Trap RNA, which can pair complementarily with the *APP* rG4 motif (Supplementary Table S1), to perform the DHX36 unwinding assay (Fig. 4A). To assess the positive control (referred to as the marker), we incubated the *APP* rG4 WT and Trap RNA constructs (Supplementary Table S1) after thermal denaturation without DHX36. Our results showed a stronger duplex band and a lighter rG4 band (indicated by the black arrow, Lane 1, Fig. 4B). Notably, when DHX36 was present and unfolded the *APP* rG4 in the presence of ATP, the Trap RNA paired with the unfolded construct, resulting in the formation of duplex and rG4 bands with similar intensity to Lane 1 (Lane 6, Fig. 4B). The duplex could not form in the absence of either DHX36 or ATP (Lanes 3 and 4) or when ATP was replaced with AMP-PNP, a non-hydrolysable analog of ATP (Lane 5, Fig. 4B). Overall, we demonstrated that DHX36 could bind and unfold *APP* 3′ UTR rG4 *in vitro*.

To investigate the effect of rG4-PROTACs on *APP* gene regulation in cells, we constructed a luciferase reporter plasmid by introducing *APP* rG4 WT or rG4 Mut motif (Supplementary Table S1) into the 3′ UTR of the Renilla gene, referred to as *APP* rG4 WT or *APP* rG4 Mut. The luciferase signal was normalized to the firefly luciferase in the same plasmid (Fig. 4C). The *APP* rG4 WT construct group showed a 36.03 ± 1.02% reduction in normalized luciferase activity than that of the *APP* rG4 Mut construct (Fig. 4D and Supplementary Fig. S14; the first black columns), suggesting that the *APP* rG4 motif negatively regulates gene expression. Importantly, treatment of rG4_A in cells inhibited the luciferase activity of *APP* rG4 WT construct (Fig. 4D), but not that of *APP* rG4 Mut construct (Supplementary Fig. S14), in a concentration-dependent manner. As the negative control, treatment of rG4 mut_A in cells cannot modulate luciferase activity either in *APP* rG4 WT or *APP* rG4 Mut constructs (Fig. 4D and Supplementary Fig. S14). Finally, the mRNA levels of dual luciferase genes were assessed using RT-qPCR, revealing no significant changes. (Supplementary Fig. S15A and B), indicating that rG4_A affect the translational level but not the transcriptional level.

To further substantiate the effect of the rG4-PROTACs on rG4-mediated gene regulation above, the *APP* native protein expression was determined by constructs containing the *APP* full-length coding sequence, the Myc tag, and the rG4 wildtype or mutant motifs (Supplementary Table S1) in the 3′ UTR, coined to *APP* native WT (Fig. 4E) and *APP* native Mut (Supplementary Fig. S16A). The *APP* native WT plasmid was co-transfected with various concentrations of rG4_A or rG4 mut_A treatment (Fig. 4E and Supplementary Fig. S17A). Western blotting analysis revealed that the amount of native *APP* protein expression declined and coincided with an increased dose of rG4_A (Fig. 4F and Supplementary Fig. S16A). In addition, to evaluate whether rG4_A can suppress endogenous APP protein expression by promoting *APP* 3′ UTR rG4 structure, we transfected Hela cells with different concentrations of rG4_A (0−500 nM) and detected the endogenous APP level by western blot. The APP protein showed a concentration-dependent declination, followed by a slight increase at higher doses of rG4_A. This trend was consistent with the expression pattern of the DHX36 protein. (Fig. 4G and Supplementary Fig. S16B). In contrast, the normalized *APP* expression in *APP* native WT groups did not exhibit a similar protein-suppressing effect in the presence of rG4 mut_A (Supplementary Fig. S18B and C). We also examined the effect of rG4_A on gene expression in native *APP* Mut construct and there was no difference was observed under rG4_A treatment (Supplementary Fig. S17B and C), which verified that the rG4-PROTACs only interfere with rG4-mediated gene regulation. Moreover, neither rG4_A nor rG4 mut_A can affect the mRNA expression of APP gene, indicating that rG4_PROTACs affect the translational level but not the transcriptional level (Supplementary Fig. S19A and B). Taken together, these data suggested that rG4_A could induce the formation of rG4 motif by degrading DHX36 protein, thereby negatively regulating rG4-mediated *APP* gene translation, resulting in the reduction of *APP* protein in cells.

### rG4-PROTACs reduce *Gnai2* protein expression and impact proliferative capacity in myoblast and muscle stem cells

The regeneration capability of skeletal muscle plays important roles in the growth, repair, and homeostasis, which is mainly dependent on its myosatellite cells (also known as skeletal muscle SCs) [48]. Previous studies revealed that *Gnai2* (an α subunit of G protein) was required for skeletal muscle growth and muscle SC differentiation [49]. Recently, we revealed that DHX36 specifically regulates the translation of *Gnai2* mRNA by unwinding the rG4 motif at the 5′ UTR, which is essential for the regenerative ability of SCs [20]. To further substantiate the effect of the rG4-PROTACs on the rG4 motif in different regions of mRNA and in different mammalian cells, we examined Gnai2 gene expression and associated biological behaviors, such as cell proliferative potential, following the inactivation of DHX36 in mouse myoblast cells and SCs. We began by verifying the binding affinity of the *Gnai2* rG4 towards DHX36 protein. For this, we prepared a FAM-labeled rG4 WT motif (Supplementary Table S1), derived from the 5′ UTR of *Gnai2*. This motif has previously been shown to mediate the modulation of *Gnai2* mRNA translation by DHX36, as demonstrated in an EGFP reporter assay [20]. We used MST to assess the binding. The *Gnai2* WT exhibited strong binding affinity (*K*_d_ = 37.9 ± 3.6 nM) and specificity to DHX36, with no binding detected in *Gnai2* mutant (Mut) (Supplementary Fig. S20).

**Figure 5.**
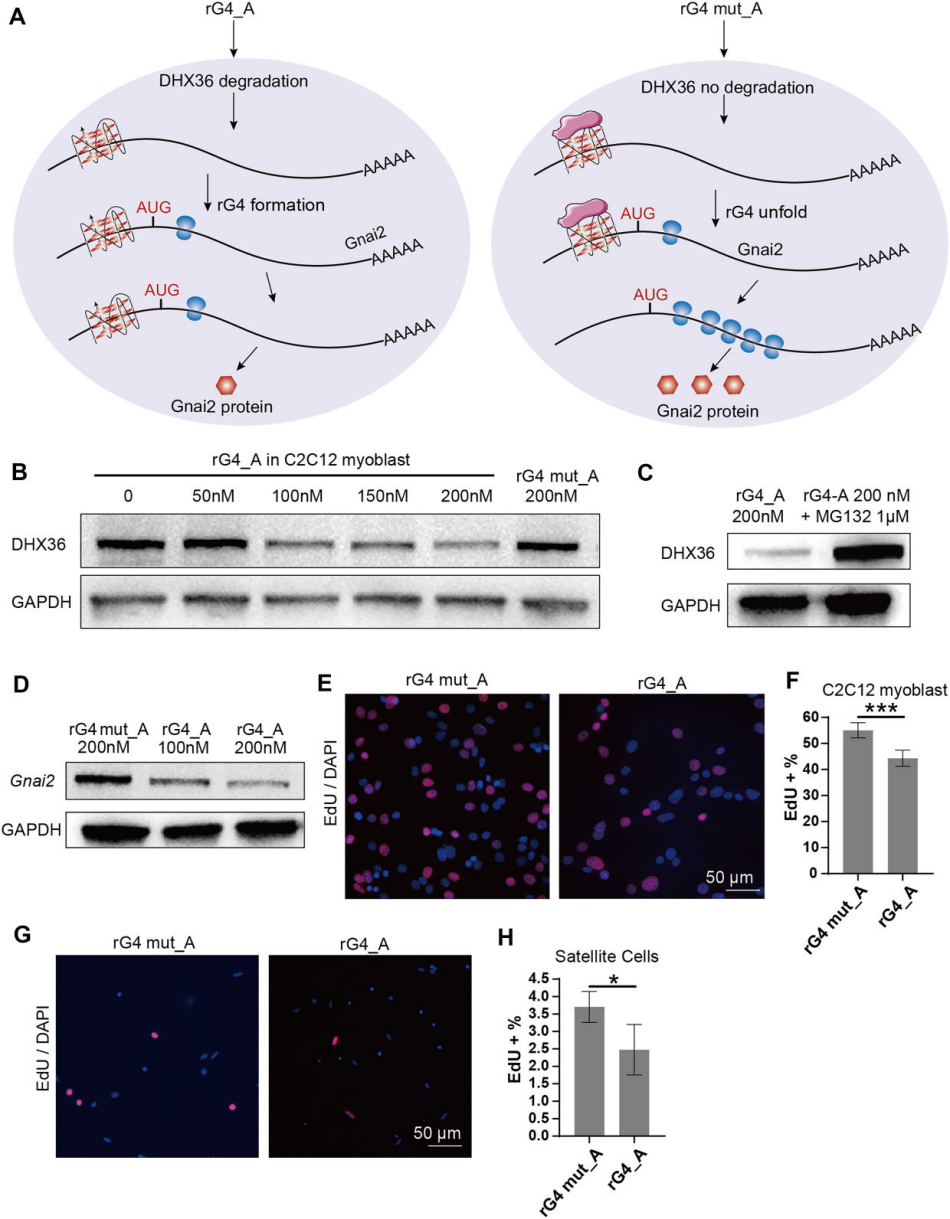
rG4_A impacts the proliferative capacity of SCs and C2C12 cells by negatively regulating *Gnai2* expression. (**A**) Illustration of translation inhibition of rG4_PROTACs on endogenous *Gnai2* gene. In C2C12 cells, rG4_A treatment induced the deletion of DHX36 protein and increased rG4 motif formation in the 5′UTR of *Gani2* transcript, resulting in the Gnai2 protein expression suppression. (**B**) rG4_A inhibits DHX36 protein expression in a concentration-dependent manner in C2C12 cells. (**C**) MG132 blocked rG4_A-induced DHX36 protein loss. C2C12 cells were treated with rG4_A (200 nM) with or without MG132 (1 μg/ml), and the cells were harvested for western blotting analysis of DHX36. (**D**) Gnai2 protein was reduced with an increased dose of rG4_A. Quantification diagrams of the western blot are shown in Supplementary Fig. S21. Normalized protein expression was obtained from biological triplicates with the standard deviation as an error bar. (**E**) The effect of rG4-PROTACs on proliferative capacity was investigated by EdU assay in C2C12 cells. Representative images are shown. Scale bar = 50 μm. (**F**) Quantitative analysis of the relative EdU incorporation percentage from five randomly selected fields per sample of panel (E). (**G**) The effect of rG4-PROTACs on proliferative capacity was investigated by EdU assay in SCs. Representative images are shown. Scale bar = 50 μm. (**H**) Quantitative analysis of the relative EdU incorporation percentage from five randomly selected fields per sample of panel (G). The relative EdU incorporation percentage is obtained from three biological replicates with the standard deviation as an error bar. *P < 0.05 and ***P < 0.001.

In contrast to the *APP* rG4 motif in the 3′ UTR mentioned earlier, we monitored the expression of *Gnai2* containing an rG4 motif in the 5′ UTR after rG4-PROTACs treatment in C2C12 mouse myoblast cells (Fig. 5A). After increasing concentrations of rG4_A treatment, DHX36 showed a dose-dependent degradation while rG4 mut_A could not degrade DHX36 (Fig. 5B and Supplementary Fig. S21A). Moreover, the degradation effect of rG4_A on DHX36 can be blocked by the proteasome inhibitor MG132 (Fig. 5C and Supplementary Fig. S21B). Simultaneously, the downstream effector gene *Gnai2* also showed a concentration-dependent down-regulation in C2C12 myoblast (Fig. 5D and Supplementary Fig. S21C), implying that rG4_A promoted the formation of rG4 at *Gnai2* 5′UTR by degrading DHX36 protein, and triggered the translation inhibition of *Gnai2* protein.

Cell proliferation is a crucial process necessary for myogenesis and muscle regeneration [50, 51]. To evaluate the effect of rG4_A on the proliferative capacity of C2C12 myoblasts and SCs, we measured EdU incorporation. This method is a sensitive and reliable way to detect and quantify cell proliferation in live cells using fluorescence microscopy [52]. In our observations, 55.06% ± 2.94 of C2C12 myoblasts treated with rG4 mut_A were EdU-positive, whereas only 44.33% ± 3.08 of those treated with rG4_A were EdU-positive (Fig. 5E and F). The inhibitory effect of rG4_A on proliferation was even more pronounced in SCs; 3.70% ± 0.44 of SCs were EdU-positive in the rG4 mut_A treatment, compared to only 2.47% ± 0.72 in the rG4_A treatment (Fig. 5G and H). The EdU assay indicated that the proliferative abilities of SCs and C2C12 myoblasts were compromised by the DHX36-*Gnai2* regulatory axis following rG4_A treatment. Overall, our findings demonstrate that DHX36 is essential for the proliferation of SCs and their derived myoblasts, suggesting that rG4-PROTACs are promising tools for controlling DHX36-mediated rG4 gene expression in mammalian cells.

## Discussion

**Figure 6.**
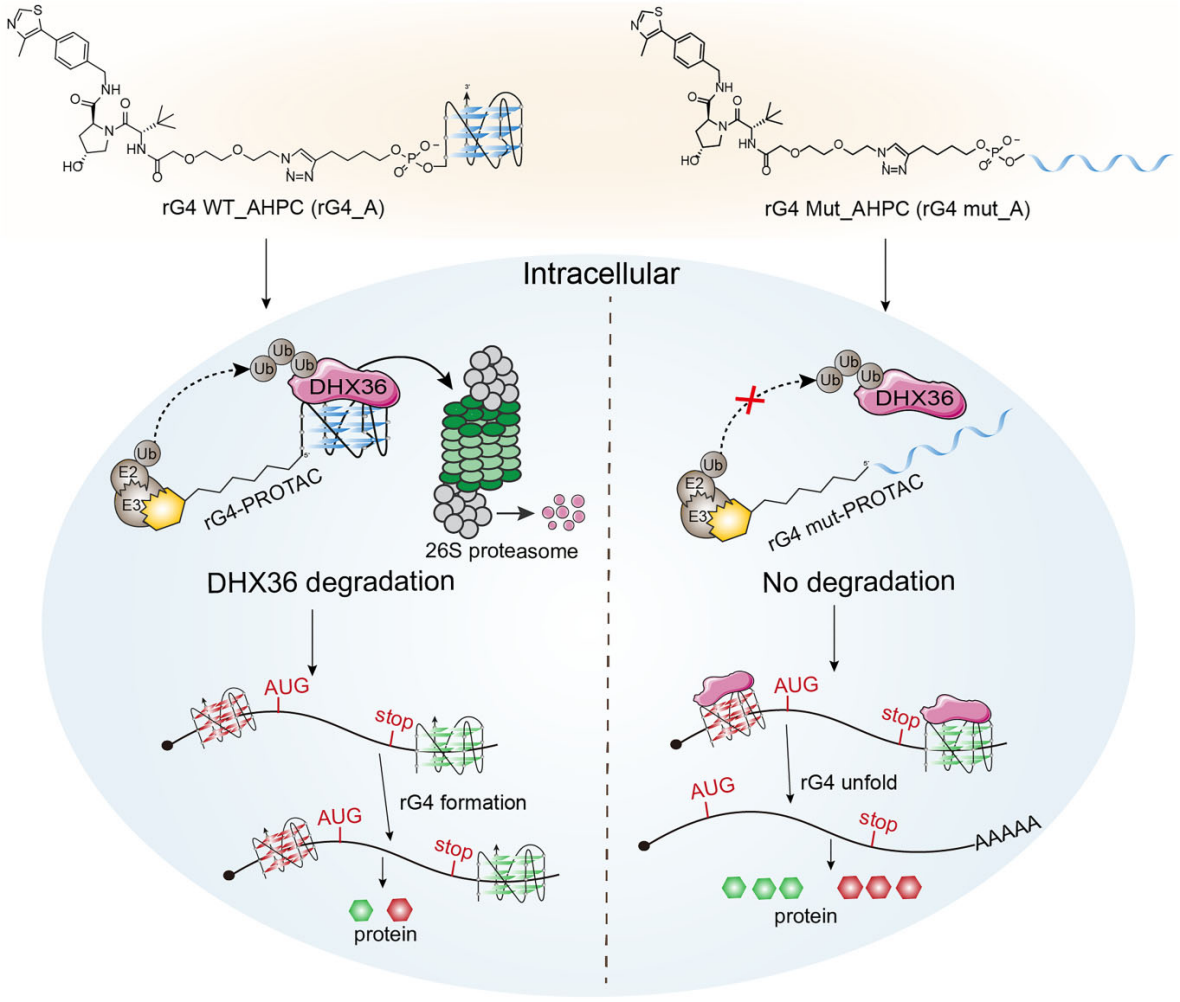
Schematic illustration of the functional mechanism of rG4-PROTACs in regulating rG4-mediated gene translation by degradation DHX36 protein. After transfection into cells, treatment with rG4_A caused a significant DHX36 degradation, which promoted rG4 motif formation and resulted in translation inhibition. While rG4 mut_A showed no effect on DHX36 protein expression and unfolded rG4 structures to induce the translation of the downstream genes.

In this study, we successfully developed rG4-PROTACs for the efficient degradation of G4BP and the regulation of translation for rG4-containing transcripts within cells. Notably, DHX36, a well-studied rG4 unwinding helicase, can be degraded by rG4_A. This approach outperforms existing techniques, such as siRNAs and dG4-PROTACs, leading to a significant reduction in the expression of APP and Gnai2 proteins (Fig. 6). Therefore, this novel rG4-PROTACs platform is valuable for understanding the interactions between rG4 and G4BP and has the potential to target dysregulated rG4-G4BP complex formation in biological contexts and diseases.

Although rG4-PROTACs and dG4-PROTACs used two similar PROTAC anchors, their binding affinity and degradation efficiency for DHX36 differ significantly. In the study on dG4-PROTACs [33], conjugation of dG4 with pomalidomide derivatives (CRBN recruiters with linkers C11, C6, and C2) and VH032 (VHL recruiter with an alkyl linker) led to a comparable level of DHX36 degradation in HeLa cells after 24 h at a dose of 50 nM. By examining the effect of linker types on the binding affinity between rG4-PROTACs and DHX36, we found that the affinity of pomalidomide and AHPC with PEG_2_ for its target is slightly stronger than that of the conjugate with the C6 linker. Interestingly, when both E3 ligase recruiters used PEG_2_ as a linker, the binding affinity of rG4_A_PEG_2_ (rG4_A) was twice as high as that of rG4_P_PEG_2_ (Fig. 2). This indicates that rG4-PROTACs that utilize VHL E3 ligands as a proteasome recruitment module are more effective than those using cereblon E3 ligands. Consequently, we designed an optimal AHPC with PEG_2_ for strong binding affinity when covalently coupled with rG4. rG4-A demonstrates a binding affinity to RHAU53 peptide and DHX36 protein that is four times stronger than that of dG4-A (Fig. 2). This likely contributes to the lesser effectiveness of dG4-A in degrading DHX36 compared to rG4-A. Data shows that treatment of HeLa cells with rG4-A can achieve over 80% reduction of DHX36, while dG4-A treatment results in only up to 50% degradation of the protein (Supplementary Fig. S11).

We provide a comprehensive proteomic analysis of the targets of rG4-PROTACs. Firstly, to investigate the gene expression changes induced by DHX36 degradation, we analyzed the enrichment of rG4 in significantly down-regulated proteins that could be blocked by MG132. Only three proteins—GLIPR2, STAG1, and SDE2—were found to be degraded via the ubiquitination pathway to the same extent as DHX36 (Fig. 3G). These proteins are predicted to contain rG4 motifs (Supplementary Table S3), though their involvement in G4 regulation has not yet been established. We speculated that these proteins may be directly regulated by rG4-PROTACs, as FAM-labeled rG4_A were primarily located in the cytoplasm and could not enter the nucleus (Supplementary Fig. S6). Moreover, SDE2 has been identified as a ubiquitin-fold-containing protein involved in pre-mRNA splicing [53], while STAG1 (Stromal antigen 1) is associated with DNA replication [54], suggesting some RNA-binding potential. Additionally, we observed that Ribonuclease H1 could be degraded by both rG4_A and rG4 mut_A, and its inhibition was rescued by MG132 (highlighted in yellow in Supplementary Data 1). This indicates that certain general RBPs may be affected by rG4-PROTACs. Further optimization, in combination with specific binders for G4BPs, is underway to develop a more selective tool for rG4-induced degradation. Importantly, no significant changes were noted in other known G4BPs, including NCL, DHX9, hnRNP, SRSF, and FMR1 (Supplementary Fig. S9). What is more, the proteomic analysis revealed that DHX36 does not affect the expression of dG4-containing genes, which mainly exist in the nucleus, such as c-Myc and BCL-2 (Supplementary Fig. S9A, green dot). Therefore, the regulatory effect of rG4_A on genes containing rG4 motifs is likely driven by DHX36.

The biological applications of rG4-PROTACs have been explored to reflect the important advancement of rG4-PROTACs compared to dG4-PROTACs. Thermodynamically stable rG4 structures in 5′ UTR of mRNA can impair cap-dependent translation by preventing the assembly of the translation initiation machinery at the 5′ cap of the mRNA and/or slow down its scanning process toward an AUG translation start codon [8, 55, 56]. Similar to 5′ UTR rG4s, 3′ UTR rG4s generally act as cap-dependent translation suppressors through RBPs or microRNAs [8, 57]. The human *APP* 3′ UTR rG4 and *Gnai2* 5′ UTR rG4 were used to showcase the negative regulating role of rG4-PROTACs in different regions of mRNAs. Previously, the formation of the rG4 motif was verified in the 3′UTR of *APP and* 5′UTR of *Gnai2* [20, 47]. Based on this finding, we assessed rG4-PROTAC’s ability to inhibit *APP* and *Gnai2* gene expression through regulating DHX36 binding and unfolding rG4 structure. First, the luciferase reporter assay showed that rG4-A could effectively suppress the *APP* gene translation in Hela cells (Fig. 4C and D). Furthermore, we constructed a native plasmid containing *APP* CDS, rG4 motif, and Myc tag to verify the regulatory effect of rG4-PROTACs on the expression of *APP* native protein. Western blot showed that *APP* and Myc proteins decreased in a dose-dependent manner with the degradation of DHX36 (Fig. 4E and F). Finally, we demonstrated the ability of rG4-PROTACs to inhibit endogenous *Gnai2* protein thus impacting the proliferative capacity of skeletal muscle SCs. After treatment with different concentrations of rG4-A, *Gnai2* protein is down-regulated in a concentration-dependent manner accompanied by DHX36 degradation (Fig. 5D). The EdU incorporation assay revealed an important function of rG4-A to impact the proliferative capacity of SCs and C2C12 cells by negatively regulating *Gnai2* expression (Fig. 5E, F, G, and H). Gnai2 was proven to be an indispensable post-transcriptional regulator of skeletal muscle regeneration and regulated by ubiquitinase-targeting chimeras (PROTACs), the utility of covalent chemoproteomic approaches like deubiquitinase-targeting chimeras [58] to facilitate DHX36-Gnai2 regulatory axis maybe beneficial to therapeutic purpose and is the subject of future direction.

The rG4-PROTACs have certain limitations that require further investigations and refinements. The poor cell penetration capacity of rG4-PROTACs will likely restrict them from exerting downstream biological function, and we think that covalent modifications [59, 60] and nucleotides-based delivery approach [61] may be applied to enhance the permeability. Taken together, this study highlights the potential use of rG4-PROTACs as a new approach to target functional rG4 structures and binding proteins, which has rarely been explored in the study of dG4-PROTACs and the field of RNA G4 in the past. In addition, our proof-of-concept study can be potentially extended to identify new G4 motifs regulated by G4BP and clarify the interaction mechanism between them.

## Conclusion

In summary, we used the non-canonical secondary structure rG4 as the warhead to develop the novel rG4-PROTACs, thus providing a new approach to target degradation of DHX36 with superior performance and high specificity in mammalian cells. We demonstrated the negative regulatory function of rG4-PROTACs on *APP* and *Gnai2* gene translation through the degradation of DHX36, thereby favoring *APP* and *Gnai2* rG4 formation. Importantly, the proliferative ability of myoblast and muscle SCs was impacted accompanied by Gnai2 protein reduction. Hence, rG4-PROTACs are valuable for the potential degradation of DHX36, which is dysregulated and aberrantly expressed *in vivo*.

## Supplementary Material

gkaf039_Supplemental_Files

## Data Availability

The mass spectrometry proteomics data have been deposited to the ProteomeXchange Consortium (http://proteomecentral.proteomexchange.org) via the iProX partner repository (Ma *et al.*, 2019) with the dataset identifier PXD058113.

## References

[1] Georgakopoulos-Soares I, Parada GE, Hemberg M Secondary structures in RNA synthesis, splicing and translation. Comput Struct Biotechnol J. 2022; 20:2871–4. 10.1016/j.csbj.2022.05.041. 35765654 PMC9198270

[2] Gellert M, Lipsett MN, Davies DR Helix formation by guanylic acid. Proc Natl Acad Sci USA. 1962; 48:2013–8. 10.1073/pnas.48.12.2013. 13947099 PMC221115

[3] Sen D, Gilbert W Formation of parallel four-stranded complexes by guanine-rich motifs in DNA and its implications for meiosis. Nature. 1988; 334:364–6. 10.1038/334364a0. 3393228

[4] Davis JT G-quartets 40 years later: from 5′-GMP to molecular biology and supramolecular chemistry. Angew Chem Int Ed Engl. 2004; 43:668–98. 10.1002/anie.200300589. 14755695

[5] Huppert JL, Balasubramanian S Prevalence of quadruplexes in the human genome. Nucleic Acids Res. 2005; 33:2908–16. 10.1093/nar/gki609. 15914667 PMC1140081

[6] Bolduc F, Garant JM, Allard F et al. Irregular G-quadruplexes found in the untranslated regions of human mRNAs influence translation. J Biol Chem. 2016; 291:21751–60. 10.1074/jbc.M116.744839. 27557661 PMC5076843

[7] Kwok CK, Marsico G, Sahakyan AB et al. rG4-seq reveals widespread formation of G-quadruplex structures in the human transcriptome. Nat Methods. 2016; 13:841–44. 10.1038/nmeth.3965. 27571552

[8] Lyu K, Chow EY, Mou X et al. RNA G-quadruplexes (rG4s): genomics and biological functions. Nucleic Acids Res. 2021; 49:5426–50. 10.1093/nar/gkab187. 33772593 PMC8191793

[9] Dai Y, Teng X, Zhang Q et al. Advances and challenges in identifying and characterizing G-quadruplex-protein interactions. Trends Biochem Sci. 2023; 48:894–909. 10.1016/j.tibs.2023.06.007. 37422364

[10] Matsumura K, Kawasaki Y, Miyamoto M et al. The novel G-quadruplex-containing long non-coding RNA GSEC antagonizes DHX36 and modulates colon cancer cell migration. Oncogene. 2017; 36:1191–9. 10.1038/onc.2016.282. 27797375

[11] Lyu K, Chen SB, Chow EY et al. An RNA G-quadruplex structure within the ADAR 5′UTR interacts with DHX36 helicase to regulate translation. Angew Chem Int Ed. 2022; 61:e202203553 10.1002/anie.202203553. 36300875

[12] Mou X, Liew SW, Kwok CK Identification and targeting of G-quadruplex structures in MALAT1 long non-coding RNA. Nucleic Acids Res. 2022; 50:397–410. 10.1093/nar/gkab1208. 34904666 PMC8754639

[13] Lyu K, Kwok CK A G-quadruplex structure in microRNA interferes with messenger RNA recognition and controls gene expression. Chem Commun (Camb). 2023; 59:8230–33. 10.1039/D3CC01549A. 37309572

[14] Shu H, Zhang R, Xiao K et al. G-quadruplex-binding proteins: promising targets for drug design. Biomolecules. 2022; 12:648 10.3390/biom12050648. 35625576 PMC9138358

[15] Kosiol N, Juranek S, Brossart P et al. G-quadruplexes: a promising target for cancer therapy. Mol Cancer. 2021; 20:40 10.1186/s12943-021-01328-4. 33632214 PMC7905668

[16] Heddi B, Cheong VV, Martadinata H et al. Insights into G-quadruplex specific recognition by the DEAH-box helicase RHAU: solution structure of a peptide-quadruplex complex. Proc Natl Acad Sci USA. 2015; 112:9608–13. 10.1073/pnas.1422605112. 26195789 PMC4534227

[17] Chen MC, Murat P, Abecassis K et al. Insights into the mechanism of a G-quadruplex-unwinding DEAH-box helicase. Nucleic Acids Res. 2015; 43:2223–31. 10.1093/nar/gkv051. 25653156 PMC4344499

[18] Lattmann S, Stadler MB, Vaughn JP et al. The DEAH-box RNA helicase RHAU binds an intramolecular RNA G-quadruplex in TERC and associates with telomerase holoenzyme. Nucleic Acids Res. 2011; 39:9390–404. 10.1093/nar/gkr630. 21846770 PMC3241650

[19] Lattmann S, Giri B, Vaughn JP et al. Role of the amino terminal RHAU-specific motif in the recognition and resolution of guanine quadruplex-RNA by the DEAH-box RNA helicase RHAU. Nucleic Acids Res. 2010; 38:6219–33. 10.1093/nar/gkq372. 20472641 PMC2952847

[20] Chen X, Yuan J, Xue G et al. Translational control by DHX36 binding to 5′UTR G-quadruplex is essential for muscle stem-cell regenerative functions. Nat Commun. 2021; 12:5043 10.1038/s41467-021-25170-w. 34413292 PMC8377060

[21] Thul PJ, Åkesson L, Wiking M et al. A subcellular map of the human proteome. Science. 2017; 356:eaal3321 10.1126/science.aal3321. 28495876

[22] Chen X, Xue G, Zhao J et al. Lockd promotes myoblast proliferation and muscle regeneration via binding with DHX36 to facilitate 5′ UTR rG4 unwinding and Anp32e translation. Cell Rep. 2022; 39:110927 10.1016/j.celrep.2022.110927. 35675771

[23] Sakamoto KM, Kim KB, Kumagai A et al. Protacs: chimeric molecules that target proteins to the Skp1-Cullin-F box complex for ubiquitination and degradation. Proc Natl Acad Sci USA. 2001; 98:8554–9. 10.1073/pnas.141230798. 11438690 PMC37474

[24] Buckley DL, Van Molle I, Gareiss PC et al. Targeting the von Hippel–Lindau E3 ubiquitin ligase using small molecules to disrupt the VHL/HIF-1α interaction. J Am Chem Soc. 2012; 134:4465–8. 10.1021/ja209924v. 22369643 PMC3448299

[25] Békés M, Langley DR, Crews CM PROTAC targeted protein degraders: the past is prologue. Nat Rev Drug Discov. 2022; 21:181–200. 10.1038/s41573-021-00371-6. 35042991 PMC8765495

[26] Verma R, Mohl D, Deshaies RJ Harnessing the power of proteolysis for targeted protein inactivation. Mol Cell. 2020; 77:446–60. 10.1016/j.molcel.2020.01.010. 32004468

[27] Ghidini A, Cléry A, Halloy F et al. RNA-PROTACs: degraders of RNA-binding proteins. Angew Chem Int Ed Engl. 2021; 60:3163–9. 10.1002/anie.202012330. 33108679 PMC7898822

[28] Samarasinghe KTG, Jaime-Figueroa S, Burgess M et al. Targeted degradation of transcription factors by TRAFTACs: TRAnscription factor TArgeting Chimeras. Cell Chem Biol. 2021; 28:648–61. 10.1016/j.chembiol.2021.03.011. 33836141 PMC8524358

[29] Liu J, Chen H, Kaniskan H et al. TF-PROTACs enable targeted degradation of transcription factors. J Am Chem Soc. 2021; 143:8902–10. 10.1021/jacs.1c03852. 34100597 PMC8225582

[30] Miao Y, Gao Q, Mao M et al. Bispecific aptamer chimeras enable targeted protein degradation on cell membranes. Angew Chem Int Ed Engl. 2021; 60:11267–71. 10.1002/anie.202102170. 33634555

[31] He S, Gao F, Ma J et al. Aptamer-PROTAC conjugates (APCs) for tumor-specific targeting in breast cancer. Angew Chem Int Ed Engl. 2021; 60:23299–305. 10.1002/anie.202107347. 34240523

[32] Zhang L, Li L, Wang X et al. Development of a novel PROTAC using the nucleic acid aptamer as a targeting ligand for tumor selective degradation of nucleolin. Mol Ther Nucleic Acids. 2022; 30:66–79. 10.1016/j.omtn.2022.09.008. 36250201 PMC9535278

[33] Patil KM, Chin D, Seah HL et al. G4-PROTAC: targeted degradation of a G-quadruplex binding protein. Chem Commun (Camb). 2021; 57:12816–9. 10.1039/D1CC05025G. 34783801

[34] Green MR, Sambrook J Precipitation of DNA with Ethanol. Cold Spring Harb Protoc. 2016; 2016:10.1101/pdb.prot093377. 27934690

[35] Zhang X, Yu L, Ye S et al. MOV10L1 Binds RNA G-quadruplex in a structure-specific manner and resolves it more efficiently than MOV10. iScience. 2019; 17:36–48. 10.1016/j.isci.2019.06.016. 31252377 PMC6600044

[36] Booy EP, Meier M, Okun N et al. The RNA helicase RHAU (DHX36) unwinds a G4-quadruplex in human telomerase RNA and promotes the formation of the P1 helix template boundary. Nucleic Acids Res. 2012; 40:4110–24. 10.1093/nar/gkr1306. 22238380 PMC3351167

[37] Mou X, Kwok CK Effect of RNA sequence context and stereochemistry on G-quadruplex-RHAU53 interaction. Biochem Biophys Res Commun. 2020; 533:1135–41. 10.1016/j.bbrc.2020.09.045. 33041003

[38] Bondeson DP, Mares A, Smith IE et al. Catalytic *in vivo* protein knockdown by small-molecule PROTACs. Nat Chem Biol. 2015; 11:611–7. 10.1038/nchembio.1858. 26075522 PMC4629852

[39] Fischer ES, Böhm K, Lydeard JR et al. Structure of the DDB1-CRBN E3 ubiquitin ligase in complex with thalidomide. Nature. 2014; 512:49–53. 10.1038/nature13527. 25043012 PMC4423819

[40] Baraniak D, Boryski J Triazole-modified nucleic acids for the application in bioorganic and medicinal chemistry. Biomedicines. 2021; 9:628 10.3390/biomedicines9060628. 34073038 PMC8229351

[41] Fantoni NZ, El-Sagheer AH, Brown T A Hitchhiker's guide to click-chemistry with nucleic acids. Chem Rev. 2021; 121:7122–54. 10.1021/acs.chemrev.0c00928. 33443411

[42] Douglass EF Jr., Miller CJ, Sparer G et al. A comprehensive mathematical model for three-body binding equilibria. J Am Chem Soc. 2013; 135:6092–99. 10.1021/ja311795d. 23544844 PMC3717292

[43] Riching KM, Caine EA, Urh M et al. The importance of cellular degradation kinetics for understanding mechanisms in targeted protein degradation. Chem Soc Rev. 2022; 51:6210–21. 10.1039/D2CS00339B. 35792307

[44] Guo JU, Bartel DP RNA G-quadruplexes are globally unfolded in eukaryotic cells and depleted in bacteria. Science. 2016; 353:aaf5371 10.1126/science.aaf5371. 27708011 PMC5367264

[45] Ashburner M, Ball CA, Blake JA et al. Gene Ontology: tool for the unification of biology. The Gene Ontology Consortium. Nat Genet. 2000; 25:25–9. 10.1038/75556. 10802651 PMC3037419

[46] Herviou P, Le Bras M, Dumas L et al. hnRNP H/F drive RNA G-quadruplex-mediated translation linked to genomic instability and therapy resistance in glioblastoma. Nat Commun. 2020; 11:2661 10.1038/s41467-020-16168-x. 32461552 PMC7253433

[47] Lyu K, Chen SB, Chan CY et al. Structural analysis and cellular visualization of APP RNA G-quadruplex. Chem Sci. 2019; 10:11095–102. 10.1039/C9SC02768H. 32206258 PMC7069244

[48] Relaix F, Bencze M, Borok MJ et al. Perspectives on skeletal muscle stem cells. Nat Commun. 2021; 12:692 10.1038/s41467-020-20760-6. 33514709 PMC7846784

[49] Minetti GC, Feige JN, Bombard F et al. Gαi2 signaling is required for skeletal muscle growth, regeneration, and satellite cell proliferation and differentiation. Mol Cell Biol. 2014; 34:619–30. 10.1128/MCB.00957-13. 24298018 PMC3911486

[50] Karalaki M, Fili S, Philippou A et al. Muscle regeneration: cellular and molecular events. In Vivo. 2009; 23:779–96. 19779115

[51] Yusuf F, Brand-Saberi B Myogenesis and muscle regeneration. Histochem Cell Biol. 2012; 138:187–99. 10.1007/s00418-012-0972-x. 22644378

[52] Flomerfelt FA, Gress RE Analysis of cell proliferation and homeostasis using EdU labeling. Methods Mol Biol. 2016; 1323:211–20. 10.1007/978-1-4939-2809-5_18. 26294411 PMC7490834

[53] Thakran P, Pandit PA, Datta S et al. Sde2 is an intron-specific pre-mRNA splicing regulator activated by ubiquitin-like processing. EMBO J. 2018; 37:89–101. 10.15252/embj.201796751. 28947618 PMC5753039

[54] Cuadrado A, Losada A Specialized functions of cohesins STAG1 and STAG2 in 3D genome architecture. Curr Opin Genet Dev. 2020; 61:9–16. 10.1016/j.gde.2020.02.024. 32294612

[55] Cammas A, Millevoi S RNA G-quadruplexes: emerging mechanisms in disease. Nucleic Acids Res. 2017; 45:1584–95. 28013268 10.1093/nar/gkw1280 PMC5389700

[56] Halder K, Wieland M, Hartig JS Predictable suppression of gene expression by 5′-UTR-based RNA quadruplexes. Nucleic Acids Res. 2009; 37:6811–7. 10.1093/nar/gkp696. 19740765 PMC2777418

[57] Cammas A, Desprairies A, Dassi E et al. The shaping of mRNA translation plasticity by RNA G-quadruplexes in cancer progression and therapy resistance. NAR Cancer. 2024; 6:zcae025 10.1093/narcan/zcae025. 38828391 PMC11140630

[58] Henning NJ, Boike L, Spradlin JN et al. Deubiquitinase-targeting chimeras for targeted protein stabilization. Nat Chem Biol. 2022; 18:412–21. 10.1038/s41589-022-00971-2. 35210618 PMC10125259

[59] Soutschek J, Akinc A, Bramlage B et al. Therapeutic silencing of an endogenous gene by systemic administration of modified siRNAs. Nature. 2004; 432:173–78. 10.1038/nature03121. 15538359

[60] Khan T, Weber H, DiMuzio J et al. Silencing myostatin using cholesterol-conjugated siRNAs induces muscle growth. Mol Ther Nucleic Acids. 2016; 5:e342 10.1038/mtna.2016.55. 27483025 PMC5023400

[61] Ma Y, Fenton OS A unified strategy to improve lipid nanoparticle mediated mRNA delivery using adenosine triphosphate. J Am Chem Soc. 2023; 145:19800–19811. 10.1021/jacs.3c05574. 37656876

