# Supplementary material for: RNA G-quadruplex structure-based PROTACs for targeted DHX36 protein degradation and gene activity modulation in mammalian cells
Source: Nucleic Acids Res. 2025 Jan 30;53(3):gkaf039. doi: 10.1093/nar/gkaf039 (PMC11780864; doi:10.1093/nar/gkaf039)
Supplement: gkaf039_Supplemental_Files [file gkaf039_supplemental_files.zip › Zhang_rG4_Protac_NAR_SI_R2.pdf]

Supporting Information

RNA G-Quadruplex Structure-Based PROTACs for Targeted DHX36 Protein Degradation and Gene Activity Modulation in Mammalian Cells

Kun Zhang[1, †], Qichang Nie[3, †], Maolin Li[5†], Xiaona Chen[4], Liting Zhong[5], Tianle Dai[5], Xiaofan Guo[4], Haizhou Zhao[1], Terrence Chi-Kong Lau[3], Huating Wang[4], Shuo-Bin Chen[5, *], and Chun Kit Kwok[1, 2, *]

[1] Department of Chemistry and State Key Laboratory of Marine Pollution, City University of Hong Kong, Hong Kong SAR 999077, China
[2] Shenzhen Research Institute of City University of Hong Kong, Shenzhen 518057, China
[3] Department of Biomedical Sciences, College of Veterinary Medicine and Life Sciences, City University of Hong Kong, Hong Kong SAR, China
[4] Department of Orthopaedics and Traumatology, Li Ka Shing Institute of Health Sciences, Chinese University of Hong Kong, Hong Kong SAR, China
[5] Guangdong Provincial Key Laboratory of New Drug Design and Evaluation, School of Pharmaceutical Sciences, Sun Yat-sen University, Guangzhou 510006, China

* To whom correspondence should be addressed. Tel: +852 3442 6858; Fax: +852 3442 0522; Email: ckkwok42@cityu.edu.hk
Correspondence may also be addressed to Shuo-Bin Chen. Email: chenshb8@mail.sysu.edu.cn
† The first three authors should be regarded as Joint First Authors.

Table of Contents

Table S1. Sequences of the oligonucleotides and peptides used in this study.

Table S2. List of all synthesized oligomers for the study and their MALDI-TOF/ESI analysis

Table S3. List of top 15 significantly down-regulated proteins (blue and red dots in Figure 3G) blocked by MG132

Scheme S1. Synthesis of Pomalidomide with different linkers

Scheme S2. Synthesis of AHPC with C6 linkers

Figure S1. Optimization of click reaction conditions.

Figure S2. Preparation of rG4-PROTACs by click chemistry.

Figure S3. Confirmation of FAM labeled rG4_PROTACs and dG4_PROTACs by PAGE.

Figure S4. The binding affinity of FAM_rG4_PROTACs towards RHAU53.

Figure S5. The binding affinity competition assay between FAM_rG4_A and FAM_dG4_A against RHAU53

Figure S6. FAM_rG4_A colocalized with DHX36 in Hela cells.

Figure S7. Screening of the rG4-PROTACs that degrade DHX36 in HeLa cells.

Figure S8. Targeted degradation of DHX36 in HEK293T and MCF-7 cell lines.

Figure S9. Proteomic analysis of rG4_A effectively degrades DHX36.

Figure S10. GO enrichment analysis of 44 down-regulated genes. Genes are significantly associated with protein transport and binding functions.

Figure S11. Comparison of the degradation effect of rG4-PROTACs and dG4-PROTACs on DHX36 protein.

Figure S12. Knockdown of DHX36 mediated by siRNA.

Figure S13. The binding affinity between *APP* rG4 and DHX36 detected by MST.

Figure S14. Normalized luciferase activity of cells transfected with *APP* rG4 Mut plasmid.

Figure S15. Relative luciferase mRNA expression levels of reporter assay.

Figure S16. Quantification analysis of western blot from Figure 4.

Figure S17. rG4-A does not affect the translation of APP native mutant plasmid.

Figure S18. rG4 mut_A does not affect the translation of APP native protein expression in cells.

Figure S19. Relative APP mRNA expression levels on APP native WT constructs.

Figure S20. The binding affinity between *Gnai2* rG4 and DHX36.

Figure S21. Quantification diagrams of western blot.

Table S1. Sequences of the oligonucleotides and peptides used in this study

Name [a]	Sequence (5'- 3' or N term - C term)
5' Hexynyl_*hTERC* rG4 WT	/5Hexynyl/**GGG**UU**G**C**GG**A**GGG**U**GGG**CCU
5' Hexynyl_*hTERC* rG4 Mut	/5Hexynyl/**GAG**UU**G**C**GG**A**GAG**U**GAG**CCU
5' Hexynyl_*T95-2T* dG4 WT	/5Hexynyl/TT**GGG**T**GGG**T**GGG**T**GGG**T
5' Hexynyl_*T95-2T* dG4 Mut	/5Hexynyl/TT**GTG**T**GTG**T**GTG**T**GTG**T
5' Hexynyl_*hTERC* rG4 WT_3' FAM	/5Hexynyl/**GGG**UU**G**C**GG**A**GGG**U**GGG**CCU/3-FAM/
5' Hexynyl_*hTERC* rG4 Mut_3' FAM	/5Hexynyl/**GAG**UU**G**C**GG**A**GAG**U**GAG**CCU/3-FAM/
5' Hexynyl_*T95-2T* dG4 WT_3' FAM	/5Hexynyl/TT**GGG**T**GGG**T**GGG**T**GGG**T/3-FAM/
5' Hexynyl_*T95-2T* dG4 Mut_3' FAM	/5Hexynyl/TT**GTG**T**GTG**T**GTG**T**GTG**T/3-FAM/
APP rG4 WT_ polyA	FAM-<br>AAAAAAAAAAG**GGG**C**GGG**U**GGG**GA**GGG**GAAAAAA
Trap RNA	UCCCCACCCGCCCCG
FAM_*APP* rG4 WT	FAM-CG**GGG**C**GGG**T**GGG**GA**GGG**GT
FAM_*APP* rG4 Mut	FAM-CG**AAG**C**GAG**T**GAA**GA**GAA**GT
APP rG4 WT inserted in dual luciferase vector	CG**GGG**C**GGG**T**GGG**GA**GGG**GT
APP rG4 Mut inserted in dual luciferase vector	CG**AAG**C**GAG**T**GAA**GA**GAA**GT
APP full-length coding and Myc tag sequences inserted in the native vector	**(Nhe I)**<br>**GCTAGC**GCCACC**ATG**CTGCCCGGTTTGGCACTGCTCCT<br>GCTGGCCGCCTGGACGGCTCGGGCGCTGGAGGTACCCA<br>CTGATGGTAATGCTGGCCTGCTGGCTGAACCCCAGATTG<br>CCATGTTCTGTGGCAGACTGAACATGCACATGAATGTCC<br>AGAATGGGAAGTGGGATTCAGATCCATCAGGGACCAAA<br>ACCTGCATTGATACCAAGGAAGGCATCCTGCAGTATTG<br>CCAAGAAGTCTACCCTGAACTGCAGATCACCAATGTGG<br>TAGAAGCCAACCAACCAGTGACCATCCAGAACTGGTGC<br>AAGCGGGGCCGCAAGCAGTGCAAGACCCATCCCCACTT<br>TGTGATTCCCTACCGCTGCTTAGTTGGTGAGTTTGTAAG<br>TGATGCCCTTCTCGTTCCTGACAAGTGCAAATTCTTACA<br>CCAGGAGAGGATGGATGTTTGCGAAACTCATCTTCACT<br>GGCACACCGTCGCCAAAGAGACATGCAGTGAGAAGAGT<br>ACCAACTTGCATGACTACGGCATGTTGCTGCCCTGCGGA<br>ATTGACAAGTTCCGAGGGGTAGAGTTTGTGTGTTGCCCA<br>CTGGCTGAAGAAAGTGACAATGTGGATTCTGCTGATGC<br>GGAGGAGGATGACTCGGATGTCTGGTGGGGCGGAGCAG<br>ACACAGACTATGCAGATGGGAGTGAAGACAAAGTAGTA<br>GAAGTAGCAGAGGAGGAAGAAGTGGCTGAGGTGGAAG<br>AAGAAGAAGCCGATGATGACGAGGACGATGAGGATGG<br>TGATGAGGTAGAGGAAGAGGCTGAGGAACCCTACGAA<br>GAAGCCACAGAGAGAACCACCAGCATTGCCACCACCAC<br>CACCACCACCACAGAGTCTGTGGAAGAGGTGGTTCGAG<br>TTCCTACAACAGCAGCCAGTACCCCTGATGCCGTTGACA

AGTATCTCGAGACACCTGGGGATGAGAATGAACATGCC
CATTTCCAGAAAGCCAAAGAGAGGCTTGAGGCCAAGCA
CCGAGAGAGAATGTCCCAGGTCATGAGAGAATGGGAAG
AGGCAGAACGTCAAGCAAAGAACTTGCCTAAAGCTGAT
AAGAAGGCAGTTATCCAGCATTTCCAGGAGAAAGTGGA
ATCTTTGGAACAGGAAGCAGCCAACGAGAGACAGCAGC
TGGTGGAGACACACATGGCCAGAGTGGAAGCCATGCTC
AATGACCGCCGCCGCCTGGCCCTGGAGAACTACATCAC
CGCTCTGCAGGCTGTTCCTCCTCGGCCTCGTCACGTGTT
CAATATGCTAAAGAAGTATGTCCGCGCAGAACAGAAGG
ACAGACAGCACACCCTAAAGCATTTCGAGCATGTGCGC
ATGGTGGATCCCAAGAAAGCCGCTCAGATCCGGTCCCA
GGTTATGACACACCTCCGTGTGATTTATGAGCGCATGAA
TCAGTCTCTCTCCCTGCTCTACAACGTGCCTGCAGTGGC
CGAGGAGATTCAGGATGAAGTTGATGAGCTGCTTCAGA
AAGAGCAAAACTATTCAGATGACGTCTTGGCCAACATG
ATTAGTGAACCAAGGATCAGTTACGGAAACGATGCTCT
CATGCCATCTTTGACCGAAACGAAAACCACCGTGGAGC
TCCTTCCCGTGAATGGAGAGTTCAGCCTGGACGATCTCC
AGCCGTGGCATTCTTTTGGGGCTGACTCTGTGCCAGCCA
ACACAGAAAACGAAGTTGAGCCTGTTGATGCCCGCCCT
GCTGCCGACCGAGGACTGACCACTCGACCAGGTTCTGG
GTTGACAAATATCAAGACGGAGGAGATCTCTGAAGTGA
AGATGGATGCAGAATTCCGACATGACTCAGGATATGAA
GTTCATCATCAAAAATTGGTGTTCTTTGCAGAAGATGTG
GGTTCAAACAAAGGTGCAATCATTGGACTCATGGTGGG
CGGTGTTGTCATAGCGACAGTGATCGTCATCACCTTGGT
GATGCTGAAGAAGAAACAGTACACATCCATTCATCATG
GTGTGGTGGAGGTTGACGCCGCTGTCACCCCAGAGGAG
CGCCACCTGTCCAAGATGCAGCAGAACGGCTACGAAAA
TCCAACCTACAAGTTCTTTGAGCAGATGCAGAACCCGGT
CGAGCAGAAACTCATCTCTGAAGAAGATCTGGAACAAA
AGTTGATTTCAGAAGAAGATCTGGAACAGAAGCTCATC
TCTGAGGAAGATCTGCTCGAGTCTAGAGGGCCCTTCGA
CTACAAAGACCATGACGGTGATTATAAAGATCATGACA
TCGACTACAAGGATGACGATGACAAG**TAGGCGGCCGC**
(Not Ⅰ)

APP rG4 WT inserted in the 3' UTR of native vector (*APP* native WT)

(Not Ⅰ)
GCGGCCGCACCCCCGCCACAGCAGCCTCTGAAGTTGGA
CAGCAAAACCATTGCTTCACTACCCATCGGTGTCCATTT
ATAGAATAATGTGGGAAGAAACAAACCCGTTTTATGAT
TTACTCATTATCGCCTTTTGACAGCTGTGCTGTAACACA
AGTAGATGCCTGAACTTGAATTAATCCACACATCAGTA
ATGTATTCTATCTCTCTTTACATTTTGGTCTCTATACTAC
ATTATTAATGGGTTTTGTGTACTGTAAAGAATTTAGCTG
TATCAAACTAGTGCATGAATAGATTCTCTCCTGATTATT
TATCACATAGCCCCTTAGCCAGTTGTATATTATTCTTGT
GGTTTGTGACCCAATTAAGTCCTACTTTACATATGCTTT
AAGAATCGATGGGGGATGCTTCATGTGAACGTGGGAGT
TCAGCTGCTTCTCTTGCCTAAGTATTCCTTTCCTGATCAC
TATGCATTTTAAAGTTAAACATTTTTAAGTATTTCAGAT
GCTTTAGAGAGATTTTTTTTCCATGACTGCATTTTACTGT
ACAGATTGCTGCTTCTGCTATATTTGTGATATAGGAATT
AAGAGGATACACACGTTTGTTTCTTCGTGCCTGTTTTAT
GTGCACACATTAGGCATTGAGACTTCAAGCTTTTCTTTT

	TTTGTCCACGTATCTTTGGGTCTTTGATAAAGAAAAGAA<br>TCCCTGTTCATTGTAAGCACTTTTACG**GGG**C**GGG**T**GGG**<br>GA**GGG**GTGCTCTGCTGGTCTTCAATTACCAAGAATTCTC<br>CAAAACAATTTTCTGCAGGATGATTGTACAGAATCATTG<br>CTTATGACATGATCGCTTTCTACACTGTATTACATAAAT<br>AAATTAAATAAAATAACCCCGGGCAAGACTTTTCTTTGA<br>AGGATGACTACAGACATTAAATAATCGAAGTAATTTTG<br>GGTGGGGAGAAGAGGCAGATTCAATTTTCTTTAACCAG<br>TCTGAAGTTTCATTTATGATACAAAAGAAGATGAAAAT<br>GGAAGTGGCAATATAAGGGGATGAGGAAGGCATGCCTG<br>GACAAACCCTTCTTTTAAGATGTGTCTTCAATTTGTATA<br>AAATGGTGTTTTCATGTAAATAAATACATTCTTGGAGGA<br>GCA**CAATTG (Mfe Ⅰ)**
APP rG4 Mut inserted in the 3' UTR of native vector (*APP* native Mut)	**(Not Ⅰ)**<br>**GCGGCCGC**ACCCCCGCCACAGCAGCCTCTGAAGTTGGA<br>CAGCAAAACCATTGCTTCACTACCCATCGGTGTCCATTT<br>ATAGAATAATGTGGGAAGAAACAAACCCGTTTTATGAT<br>TTACTCATTATCGCCTTTTGACAGCTGTGCTGTAACACA<br>AGTAGATGCCTGAACTTGAATTAATCCACACATCAGTA<br>ATGTATTCTATCTCTCTTTACATTTTGGTCTCTATACTAC<br>ATTATTAATGGGTTTTGTGTACTGTAAAGAATTTAGCTG<br>TATCAAACTAGTGCATGAATAGATTCTCTCCTGATTATT<br>TATCACATAGCCCCTTAGCCAGTTGTATATTATTCTTGT<br>GGTTTGTGACCCAATTAAGTCCTACTTTACATATGCTTT<br>AAGAATCGATGGGGGATGCTTCATGTGAACGTGGGAGT<br>TCAGCTGCTTCTCTTGCCTAAGTATTCCTTTCCTGATCAC<br>TATGCATTTTAAAGTTAAACATTTTTAAGTATTTCAGAT<br>GCTTTAGAGAGATTTTTTTTCCATGACTGCATTTTACTGT<br>ACAGATTGCTGCTTCTGCTATATTTGTGATATAGGAATT<br>AAGAGGATACACACGTTTGTTTCTTCGTGCCTGTTTTAT<br>GTGCACACATTAGGCATTGAGACTTCAAGCTTTTCTTTT<br>TTTGTCCACGTATCTTTGGGTCTTTGATAAAGAAAAGAA<br>TCCCTGTTCATTGTAAGCACTTTTACG**AAG**C**GAG**T**GAA**<br>GA**GAA**GTGCTCTGCTGGTCTTCAATTACCAAGAATTCTC<br>CAAAACAATTTTCTGCAGGATGATTGTACAGAATCATTG<br>CTTATGACATGATCGCTTTCTACACTGTATTACATAAAT<br>AAATTAAATAAAATAACCCCGGGCAAGACTTTTCTTTGA<br>AGGATGACTACAGACATTAAATAATCGAAGTAATTTTG<br>GGTGGGGAGAAGAGGCAGATTCAATTTTCTTTAACCAG<br>TCTGAAGTTTCATTTATGATACAAAAGAAGATGAAAAT<br>GGAAGTGGCAATATAAGGGGATGAGGAAGGCATGCCTG<br>GACAAACCCTTCTTTTAAGATGTGTCTTCAATTTGTATA<br>AAATGGTGTTTTCATGTAAATAAATACATTCTTGGAGGA<br>GCA**CAATTG (Mfe Ⅰ)**
Forward primer of Renilla gene	ACAAGTACCTCACCGCTTGG
Reverse primer of Renilla gene	GACACTCTCAGCATGGACGA
Forward primer of Firefly gene	GGACATCACCTATGCCGAGT
Reverse primer of Firefly gene	GTTCTCAGAGCACACCACGA
Forward primer of *APP* gene	GTGCTCTGCTGGTCTTCAAT
Reverse primer of *APP* gene	GAAAAGTCTTGCCCGGGGTT

Sense of DHX36 siRNA#1	GCACUUUACAUGAUCUCUUTT
Antisense of DHX36 siRNA#1	AAGAGAUCAUGUAAAGUGCTT
Sense of DHX36 siRNA#2	GGAGUCCACUUGGCACGAUTT
Antisense of DHX36 siRNA#2	AUCGUGCCAAGUGGACUCCTT
Sense of DHX36 siRNA#3	GGUGUUCGGAAAAUAGUAATT
Antisense of DHX36 siRNA#3	UUACUAUUUUCCGAACACCTT
Sense of siRNA negative control	UUCUCCGAACGUGUCACGUTT
Antisense of siRNA negative control	ACGUGACACGUUCGGAGAATT
FAM_*Gnai2* rG4 WT	FAM-**GGG**CCGGCGGU**GGG**AGCGGAGU**GGG**UC**GGG**C**GGGG**
FAM_*Gnai2* rG4 Mut	FAM-**G**A**G**CCGGCGGU**G**A**G**AGCGGAGU**G**A**G**UC**G**AAC**G**AA**G**
RHAU53	SMHPGHLKGREIGMWYAKKQGQKNKEAERQERAVVHMDERREEQIVQLLNSVQAK

[a] Table Footnote: The Gs involved in G4 quartets are in bold. The G4 Mut sequences designed by substituting some Gs with As or Ts are in yellow. Myc tag sequence is shown in blue highlight. *APP* rG4 WT or Mut motif inserted in the 3' UTR of the native vector is indicated by a gray highlight.

Table S2: List of all synthesized oligomers for the study and their MALDI-TOF/ESI analysis

Label	nucleotide sequence	Cal. MW (Da)	Obs. MW (Da)
rG4_A_PEG_2 (rG4_A)	5' Hexynyl_*hTERC* rG4 WT	$[M+H]^+$ 7311.82	$[M+H]^+$ 7311.50
rG4 mut_A_PEG_2 (rG4 mut_A_)	5' Hexynyl_*hTERC* rG4 Mut	$[M+H]^+$ 7263.82	$[M+H]^+$ 7266.08
rG4_A_C_6	5' Hexynyl_*hTERC* rG4 WT	$[M+K+Na]^+$ 7341.26	$[M+K+Na]^+$ 7337.21
rG4 mut_A_C_6	5' Hexynyl_*hTERC* rG4 Mut	$[M+H]^+$ 7231.38	$[M+H]^+$ 7226.10
rG4_P_PEG_2	5' Hexynyl_*hTERC* rG4 WT	$[M+H]^+$ 7168.15	$[M+H]^+$ 7166.37
rG4 mut_P_PEG_2	5' Hexynyl_*hTERC* rG4 Mut	$[M+H]^+$ 7120.25	$[M+H]^+$ 7123.0
rG4_P_C_6	5' Hexynyl_*hTERC* rG4 WT	$[M+H]^+$ 7108.17	$[M+H]^+$ 7103.95
rG4 mut_P_C_6	5' Hexynyl_*hTERC* rG4 Mut	$[M+H]^+$ 7060.27	$[M+H]^+$ 7063.0
rG4_P_PEG_1-C_2	5' Hexynyl_*hTERC* rG4 WT	$[M+H]^+$ 7096.46	$[M+H]^+$ 7096.30
rG4 mut_P_PEG_1-C_2	5' Hexynyl_*hTERC* rG4 Mut	$[M+H]^+$ 7048.46	$[M+H]^+$ 7052.05
dG4_A_PEG_2 (dG4_A)	5' Hexynyl_*T95-2T* dG4 WT	$[M+H]^+$ 6475.53	$[M+H]^+$ 6474.75
dG4 mut_A_PEG_2 (dG4 mut_A_)	5' Hexynyl_*T95-2T* dG4 Mut	$[M+H]^+$ 6375.52	$[M+H]^+$ 6374.88
FAM_rG4_A_PEG_2 (FAM_rG4_A)	5' Hexynyl_*hTERC* rG4 WT_3' FAM	$[M+H]^+$ 7881.22	$[M+H]^+$ 7881.85
FAM_rG4 mut_A_PEG_2 (FAM_rG4 mut_A)	5' Hexynyl_*hTERC* rG4 Mut	$[M+H]^+$ 7833.22	$[M+H]^+$ 7833.11
FAM_rG4_A_C_6	5' Hexynyl_*hTERC* rG4 WT_3' FAM	$[M+H]^+$ 7848.78	$[M+H]^+$ 7846.89
FAM_rG4 mut_A_C_6	5' Hexynyl_*hTERC* rG4 Mut_3' FAM	$[M+H]^+$ 7814.79	$[M+H]^+$7815.6
FAM_rG4_P_PEG_2	5' Hexynyl_*hTERC* rG4 WT_3' FAM	$[M+H]^+$ 7737.65	$[M+H]^+$7739.6
FAM_rG4 mut_P_PEG_2	5' Hexynyl_*hTERC* rG4 Mut_3' FAM	$[M+H]^+$ 7689.65	$[M+H]^+$ 7688.87
FAM_rG4_P_C_6	5' Hexynyl_*hTERC* rG4 WT_3' FAM	$[M+H]^+$ 7677.68	$[M+H]^+$ 7676.01
FAM_rG4 mut_P_C_6	5' Hexynyl_*hTERC* rG4 Mut_3' FAM	$[M+H]^+$ 7629.68	$[M+H]^+$ 7629.22
FAM_dG4_A_PEG_2 (FAM_dG4_A)	5' Hexynyl_*T95-2T* dG4 WT_3' FAM	$[M+H]^+$ 7045.02<br>$[M+K]^+$ 7083.02	$[M+H]^+$ 7045.12<br>$[M+K]^+$ 7083.30
FAM_dG4 mut_A_PEG_2 (FAM_dG4 mut _A)	5' Hexynyl_*T95-2T* dG4 Mut_3' FAM	$[M+H]^+$ 6944.92	$[M+H]^+$ 6942.65
FAM_rG4_P_PEG_1-C_2	5' Hexynyl_*hTERC* rG4 WT_3' FAM	$[M+H]^+$ 7665.86	$[M+H]^+$ 7665.87
FAM_rG4 mut_P_PEG_1-C_2	5' Hexynyl_*hTERC* rG4 Mut_3' FAM	$[M+H]^+$ 7617.86	$[M+H]^+$ 7617.82

Table S3: List of top 15 significantly down-regulated proteins (blue and red dots in Figure 3G) blocked by MG132

Genes	[Log_2 (rG4_A / Blank] < -1)	-0.4 < [Log_2 (rG4_A+MG132 / Blank)] < 0.31	G4BPs [a]	RBPs [a]	potential rG4s sites by G4 Atlas	Confirmed by rG4-seq(1,2)
GLIPR2	-9.97	0.006	No	No	No	Yes
DHX36	-8.77	-0.34	Yes	Yes	181	Yes
STAG1	-8.56	-0.02	No	Possible(3)	38	Yes
SDE2	-8.16	-0.24	No	Possible(4)	52	Yes
RNF14	-4.99	-0.71	No	No	No	No
MEGF9	-3.48	-0.51	No	No	136	Yes
R4462.563EH1	-7.23	0.76	No	No	No	No
MPDZ	-3.16	0.57	No	No	88	Yes
RAB42	-3.06	0.86	No	No	201	Yes
COBL	-2.82	0.15	No	No	No	No
SORBS2	-2.36	0.31	No	No	37	Yes
AAMDC	-2.2	0.75	No	No	15	Yes
ATPAF1	-2.41	-0.44	No	No	No	No
FADS2	-2.52	-0.34	No	No	10	Yes
LRRC57	-2.03	-0.3	No	No	114	Yes

[a] Table Footnote: G4BPs indicate G4 binding proteins. RBPs indicate RNA binding proteins. G4 Atlas data was obtained from *https://www.g4atlas.org/*.

Scheme S1. Synthesis of Pomalidomide with different linkers

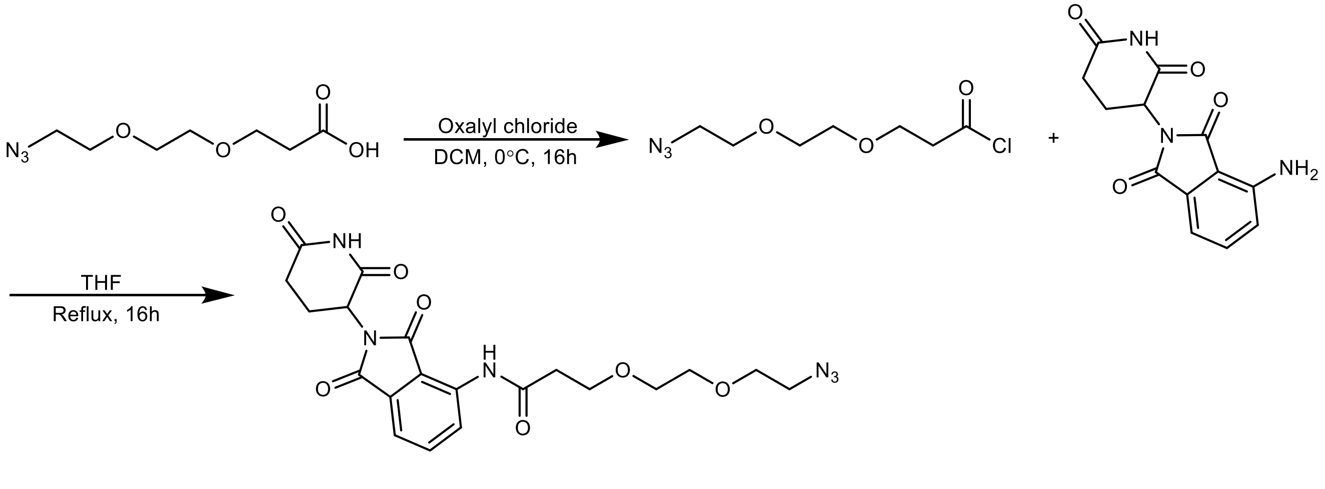

Pomalidomide-PEG_2-N_3

Synthesis of Pomalidomide-PEG_2-N_3: In a flask, 3-(2-(2-azidoethoxy)ethoxy)propanoic acid (100 mg, 0.5 mmol) was dissolved in dry dichloromethane (5.0 mL). The reaction was cooled to 0 °C and a catalytic amount of *N,N*-Dimethylformamide (10 µL) was added, followed by the dropwise addition of oxalyl chloride (212 µL, 2.5 mmol). The reaction was stirred at room temperature for 3 hours before the solvent was evaporated in vacuo and kept under argon. In another round bottom flask equipped with a reflux condenser, 4-amino-2-(2,6-dioxopiperidin-3-yl)isoindoline-1,3-dione (136 mg, 0.5 mmol) and 3-(2-(2-azidoethoxy)ethoxy)propanoyl chloride were dissolved in dry tetrahydrofuran (5.0 mL). The mixture was refluxed for 4 hours before the resulting solution was quenched with water (10 mL) and extracted with ethyl acetate (2 x 20 mL). The organic layer was combined and washed with brine, dried over anhydrous Na_2SO_4, filtered, and evaporated. The residue was applied onto a silica gel column and eluted with dichloromethane/methanol (40:1). The collected fractions were combined and concentrated. This resulted in 122 mg (yield: 53%) of 3-(2-(2-azidoethoxy)ethoxy)-*N*-(2-(2,6-dioxopiperidin-3-yl)-1,3-dioxoisoindolin-4-yl)propanamide as a white solid.

^{1}H NMR (400 MHz, $CDCl_3$) δ 9.85 (s, 1H), 8.86 (d, *J* = 8.5 Hz, 1H), 8.10 (s, 1H), 7.71 (t, *J* = 7.9 Hz, 1H), 7.55 (d, *J* = 7.3 Hz, 1H), 4.95 (dd, *J* = 12.1, 5.4 Hz, 1H), 3.87 (t, *J* = 5.7 Hz, 2H), 3.79 – 3.68 (m, 4H), 3.66 (t, *J* = 5.1 Hz, 2H), 3.34 (t, *J* = 5.1 Hz, 2H), 2.99 – 2.76 (m, 3H), 2.75 (t, *J* = 5.7 Hz, 2H), 2.23 – 2.12 (m, 1H).

^{13}C NMR (125 MHz, $CDCl_3$) δ 171.0, 170.9, 168.7, 168.0, 166.9, 137.8, 136.4, 131.4, 125.8, 118.6, 115.8, 70.8, 70.6, 70.2, 66.8, 50.8, 49.3, 38.8, 31.5, 22.8. HRMS (ESI) m/z: calcd for $C_{20}H_{22}N_6O_7$ $[M+Na]^+$ 481.1442, found 481.1439.

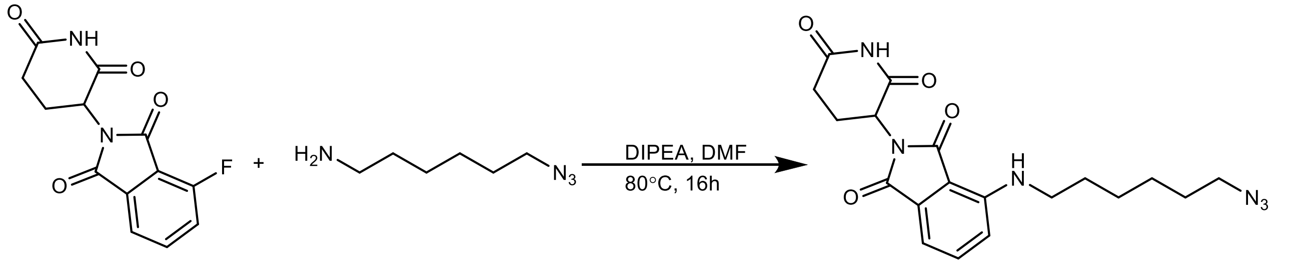

Pomalidomide-C_6-N_3

Synthesis of Pomalidomide-C_6-N_3: To a solution of 2-(2,6-dioxopiperidin-3-yl)-4-fluoroisoindoline-1,3-dione (140 mg, 0.5 mmol) and 6-azidohexan-1-amine (86 mg, 0.6 mmol) in *N,N*-Dimethylformamide (3.0 mL) was added DIPEA (139 µL , 0.8 mmol). The reaction mixture was stirred at 80 °C for 16 hours in an oil bath. The resulting solution was added with water (10 mL) and extracted with ethyl acetate (2 × 20 mL). The organic layer was combined and washed with brine, dried over anhydrous Na_2SO_4, filtered, and evaporated. The residue was applied onto a silica gel column and eluted with dichloromethane/methanol (v/v = 60:1). The collected fractions were combined and concentrated. This resulted in 110 mg (yield: 55%) of 4-((6-azidohexyl)amino)-2-(2,6-dioxopiperidin-3-yl)isoindoline-1,3-dione as a yellow solid.

^{1}H NMR (400 MHz, $CDCl_3$) δ 8.00 (s, 1H), 7.50 (t, *J* = 7.9 Hz, 1H), 7.09 (d, *J* = 7.1 Hz, 1H), 6.88 (d, *J* = 8.5 Hz, 1H), 6.23 (s, 1H), 4.91 (dd, *J* = 12.4, 5.1 Hz, 1H), 3.44 – 3.14 (m, 4H), 2.97 – 2.66 (m, 3H), 2.20 – 2.05 (m, 1H), 1.78 – 1.54 (m, 4H), 1.53 – 1.36 (m, 4H).
^{13}C NMR (125 MHz, $CDCl_3$) δ 171.2, 169.6, 168.5, 167.7, 147.1, 136.3, 132.6, 116.7, 111.6, 110.0, 51.4, 49.0, 42.6, 31.5, 29.2, 28.9, 26.6, 26.6, 22.9. HRMS (ESI) m/z: calcd for $C_{19}H_{22}N_6O_4$ $[M+H]^+$ 399.1775, found 399.1778.

Scheme S2. Synthesis of AHPC with C_6 linkers

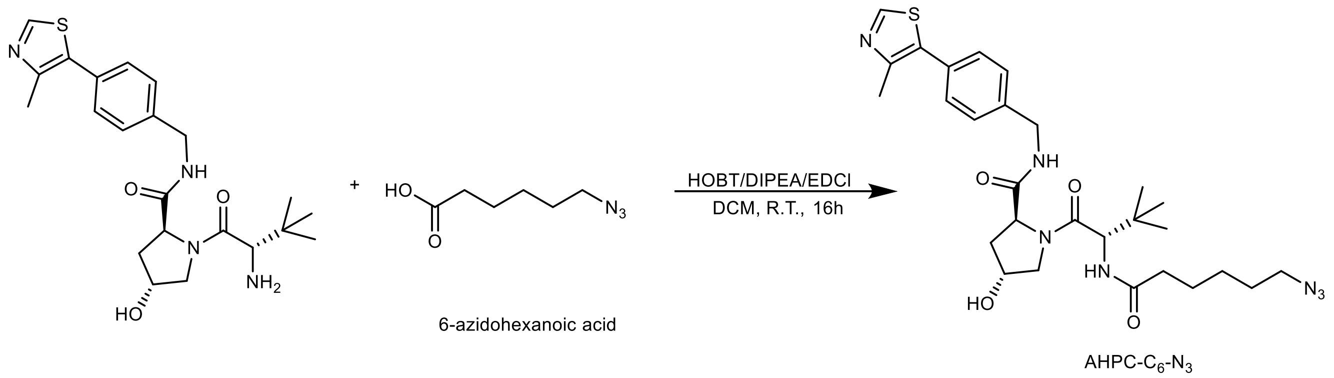

Synthesis of AHPC-C_6-N_3: To a solution of (4R)-1-((R)-2-amino-3,3-dimethylbutanoyl)-4-hydroxy-*N*-(4-(4-methylthiazol-5-yl)benzyl)pyrrolidine-2-carboxamide (172 mg, 0.4 mmol) and 7-azidoheptanoic acid (79 mg, 0.5 mmol) in dichloromethane (5.0 mL) was added HOBt (68 mg, 0.5 mmol), EDCI (96 mg, 0.5 mmol)and DIPEA (174 μL, 1.0 mmol). The reaction mixture was stirred at room temperature for 16 hours. The resulting solution was added with water (10 mL) and extracted with dichloromethane (2 × 20 mL). The organic layer was combined, washed with brine, dried over anhydrous Na_2SO_4, filtered, and evaporated. The residue was applied onto a silica gel column and eluted with dichloromethane/methanol (v/v = 20:1). The collected fractions were combined and concentrated. This resulted in 190 mg (yield: 83%) of (2S,4R)-1-((S)-2-(7-azidoheptanamido)-3,3-dimethylbutanoyl)-4-hydroxy-*N*-(4-(4-methylthiazol-5-yl)benzyl) pyrrolidine-2-carboxamide as a faint yellow liquid.
^{1}H NMR (500 MHz, $CDCl_3$) δ 8.68 (s, 1H), 7.43 – 7.29 (m, 5H), 6.26 (d, *J* = 8.9 Hz, 1H), 4.67 (t, *J* = 7.9 Hz, 1H), 4.56 – 4.48 (m, 3H), 4.32 (dd, *J* = 15.0, 5.3 Hz, 1H), 4.02 (d, *J* = 11.2 Hz, 1H), 3.62 (dd, *J* = 11.3, 3.7 Hz, 1H), 3.23 (t, *J* = 6.8 Hz, 2H), 2.49 (s, 3H), 2.44 (ddd, *J* = 12.9, 7.9, 4.6 Hz, 1H), 2.18 (t, *J* = 7.5 Hz, 2H), 2.10 (dd, *J* = 13.5, 8.1 Hz, 1H), 1.65 – 1.51 (m, 4H), 1.41 – 1.30 (m, 2H), 0.92 (s, 9H). ^{13}C NMR (125 MHz, $CDCl_3$) δ 173.5, 171.8, 171.0, 150.5, 148.5, 138.2, 131.7, 131.0, 129.6 (2C), 128.2 (2C), 70.1, 58.8, 57.5, 56.9, 51.3, 43.3, 36.3, 36.2, 35.2, 28.6, 26.5 (3C), 26.4, 25.1, 16.1. HRMS (ESI) m/z: calcd for $C_{28}H_{39}N_7O_4S$ $[M+Na]^+$ 592.2676, found 592.2676.

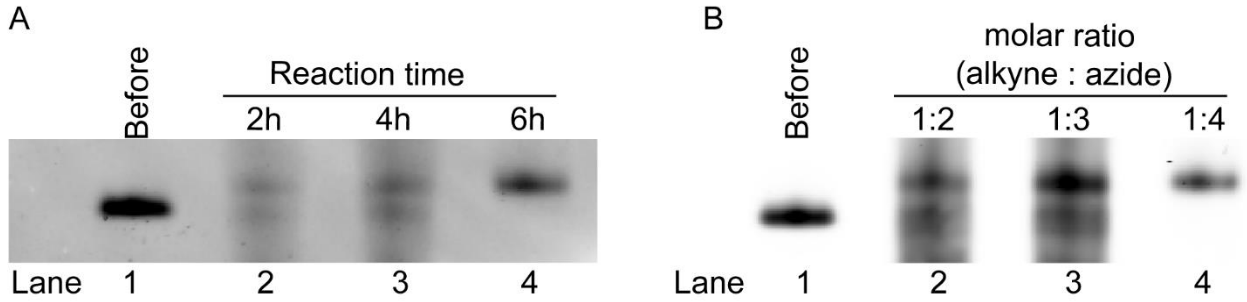

Figure S1. Optimization of click reaction conditions. (A) Optimization of reaction time for click reaction. The molar ratio of 5' hexynyl_ *hTERC* rG4 WT to (S, R, S)-AHPC-PEG$_2$-N$_3$ is 1:4. The concentration of [Cu (II)-TBTA] and ascorbic acid is 500 μM in all the samples. Lane 2: 2h (cyclization yield: 49%); Lane 3: 4h (cyclization yield: 62%); Lane 4: 6h (cyclization yield: 91%). Reaction time of 6 h was selected for further investigation. (B) Optimization of molar ratio for click reaction. The concentration of [Cu (II)-TBTA] and ascorbic acid is 500 μM in all the samples. Reactions were performed for 6 h. Lane 2: 1:2 (cyclization yield: 47%); Lane 3: 1:3 (cyclization yield: 60%); Lane 4: 1:4 (cyclization yield: 92%). The molar ration (alkyne: azide =1:4) was selected for click reaction between 5' hexynyl oligonucleotides and azide-modified E3 ligase ligands.

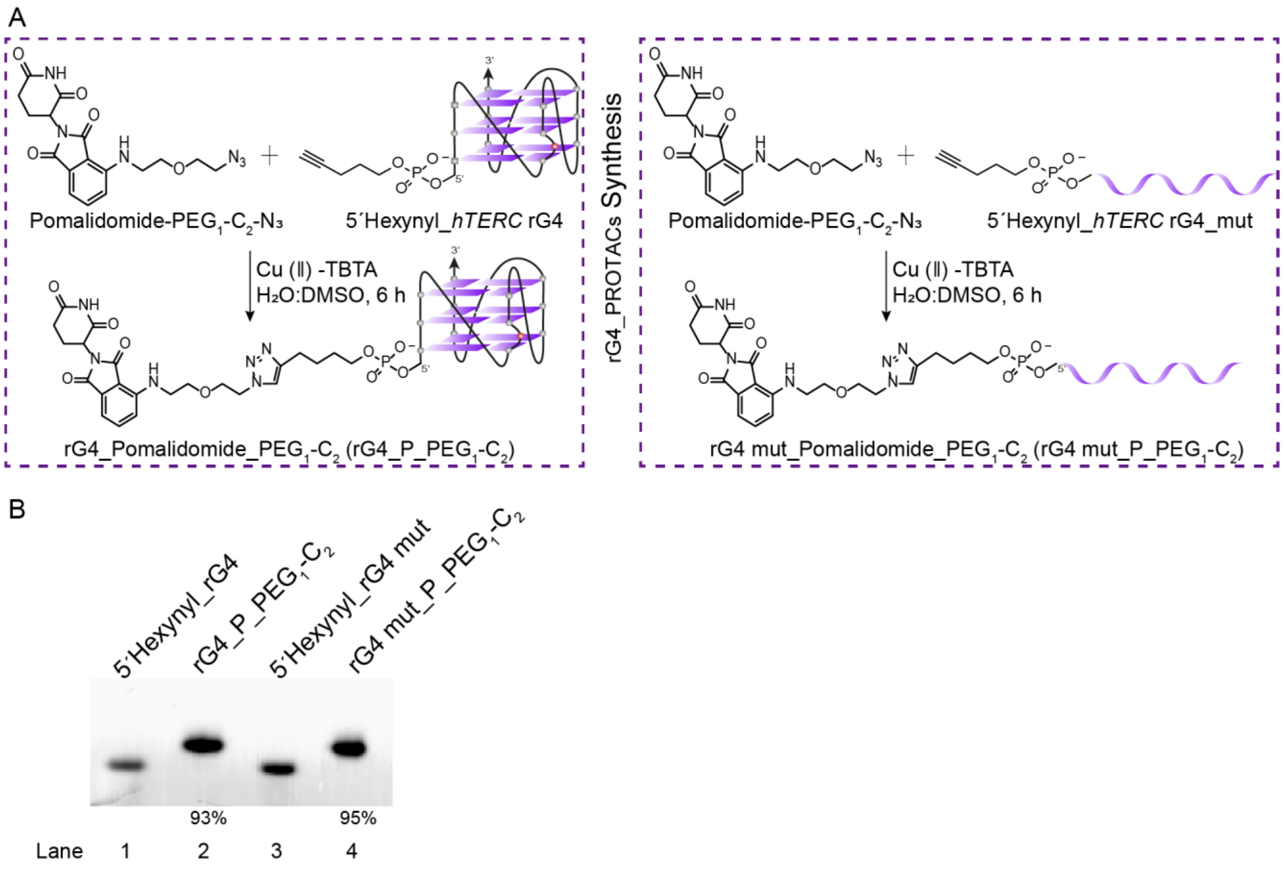

Figure S2. Preparation of rG4-PROTACs by click chemistry. (A) Schematic representation of the synthesis of rG4-PROTACs between azide-modified Pomalidomide-PEG_1-C_2-N_3 and alkyne-modified rG4 oligos (Table S1) to generate rG4-PROTACs (rG4_P_PEG_1-C_2, rG4 mut_P_PEG_1-C_2). (B) Confirmation of rG4-Pomalidomide-PEG1-C_2-N_3 conjugates. The click reaction products were subjected to 12% denaturing PAGE at 300 V for 30 min.

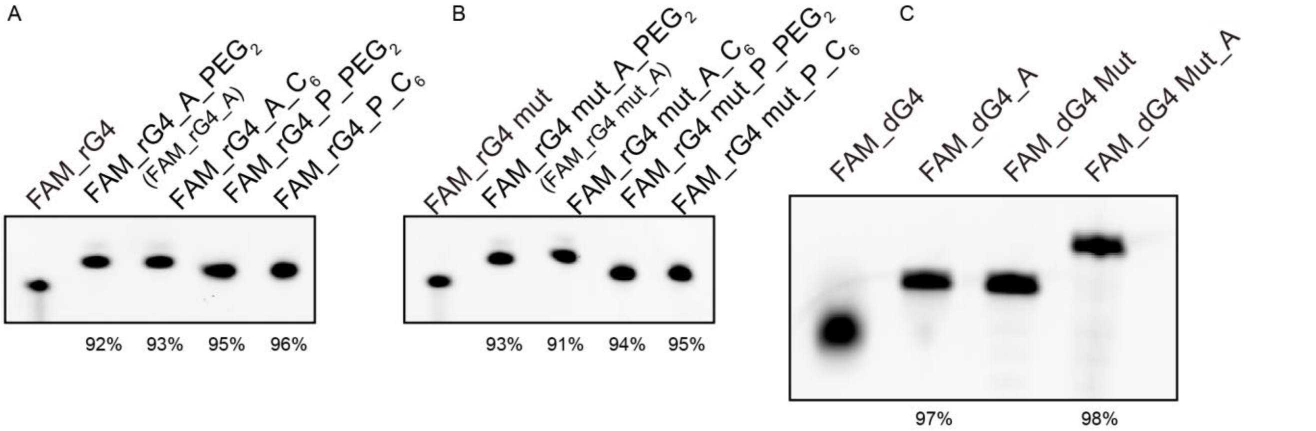

Figure S3. Confirmation of FAM labeled rG4_PROTACs and dG4_PROTACs by PAGE. (A) Denaturing PAGE analysis of FAM labeled rG4 WT_PROTACs after click reaction. (B) Denaturing PAGE analysis of FAM labeled rG4 Mut_PROTACs after click reaction. (C) Denaturing PAGE analysis of FAM_dG4_A and FAM_dG4 Mut_A after click reaction.

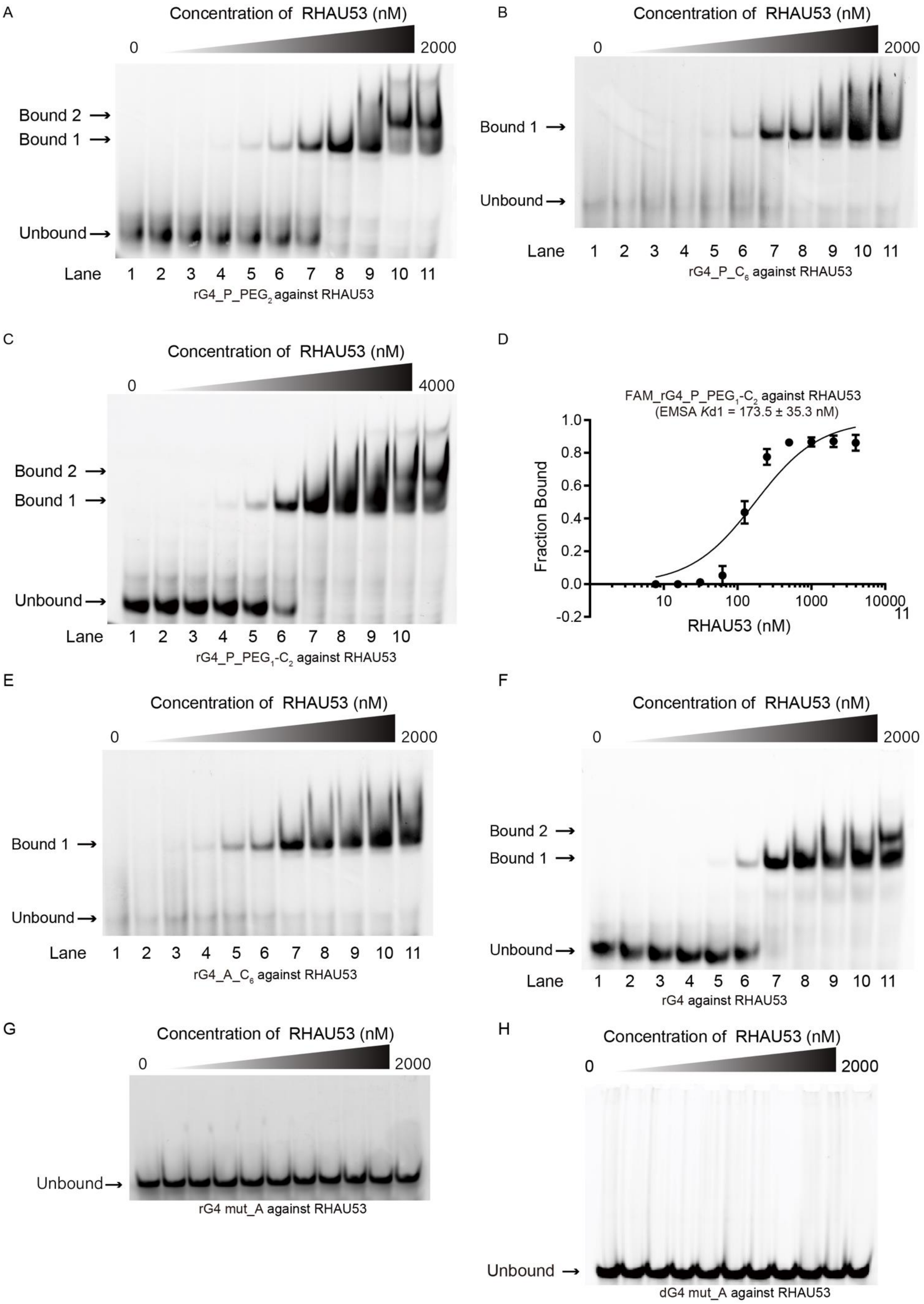

A
Concentration of RHAU53 (nM)
0
2000
Bound 2
Bound 1
Unbound
Lane 1 2 3 4 5 6 7 8 9 10 11
rG4_P_PEG2 against RHAU53
B
Concentration of RHAU53 (nM)
0
2000
Bound 1
Unbound
Lane 1 2 3 4 5 6 7 8 9 10 11
rG4_P_C6 against RHAU53
C
Concentration of RHAU53 (nM)
0
4000
Bound 2
Bound 1
Unbound
Lane 1 2 3 4 5 6 7 8 9 10
rG4_P_PEG1-C2 against RHAU53
D
FAM_rG4_P_PEG1-C2 against RHAU53
(EMSA Kd1 = 173.5 ± 35.3 nM)
Fraction Bound
1.0
0.8
0.6
0.4
0.2
0.0
-0.2
10
100
1000
10000
11
RHAU53 (nM)
E
Concentration of RHAU53 (nM)
0
2000
Bound 1
Unbound
Lane 1 2 3 4 5 6 7 8 9 10 11
rG4_A_C6 against RHAU53
F
Concentration of RHAU53 (nM)
0
2000
Bound 2
Bound 1
Unbound
Lane 1 2 3 4 5 6 7 8 9 10 11
rG4 against RHAU53
G
Concentration of RHAU53 (nM)
0
2000
Unbound
rG4 mut_A against RHAU53
H
Concentration of RHAU53 (nM)
0
2000
Unbound
dG4 mut_A against RHAU53

Figure S4. The binding affinity of FAM_rG4_PROTACs towards RHAU53. (A-C) Native Gel of the binding between FAM_rG4_pomalidomide PROTACs and RHAU53 detected by EMSA. Binding curves are shown in Figure 2A. (D) Binding curves of FAM_rG4_P_PEG$_1$_C$_2$ against RHAU53 from the first shift bound (Bound 1) and the K_d1 was calculated to be 173.5 ± 35.3 nM. (E) Native Gel of the binding between FAM_rG4_A_C$_6$ and RHAU53 detected by EMSA. Binding curves are shown in Figure 2A. (F) Native Gel of the binding between FAM_rG4 and RHAU53 detected by EMSA. Binding curves are shown in Figure 2A. (G-H) The binding analysis of FAM_rG4 mut_A and FAM_dG4 mut_A to RHAU53 and no binding was observed.

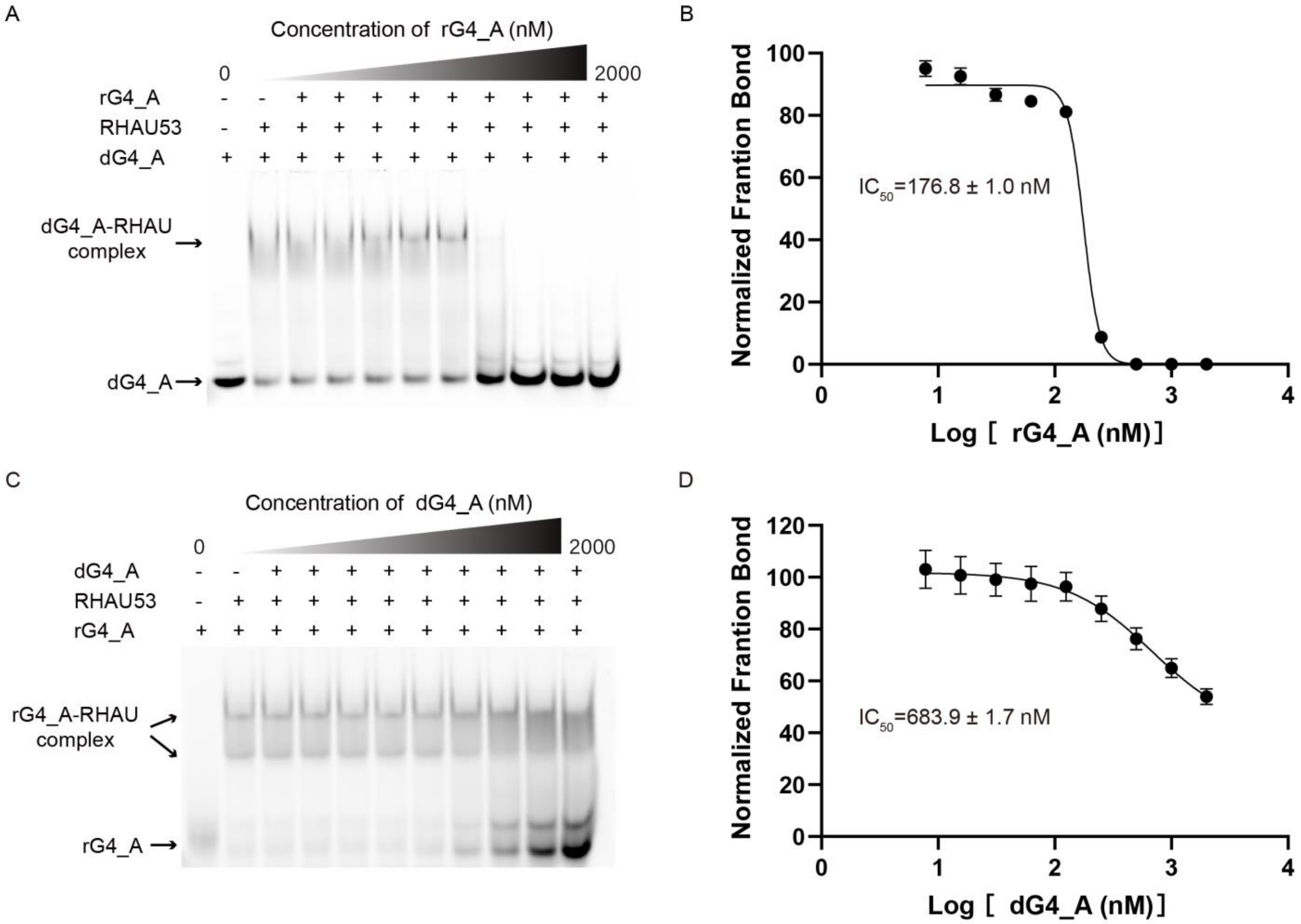

Figure S5. The binding affinity competition assay between FAM_rG4_A and FAM_dG4_A against RHAU53. (A) EMSA shows that the binding of RHAU53 and FAM_dG4_A is inhibited by FAM_rG4_A (0-2000 nM). (B) The inhibition curve of rG4_A on FAM_dG4_A-RHAU53 complex generated from A. IC_{50} was 176.8 ± 1.0 nM. (C) The binding of RHAU53 and FAM_rG4_A couldn't be fully inhibited by dG4_A (0-2000 nM). (D) The inhibition curve of dG4_A on FAM_rG4_A-RHAU53 complex generated from C. IC_{50} was 683.9 ± 1.7 nM. The Data were obtained from three replicates with the standard deviation as an error bar.

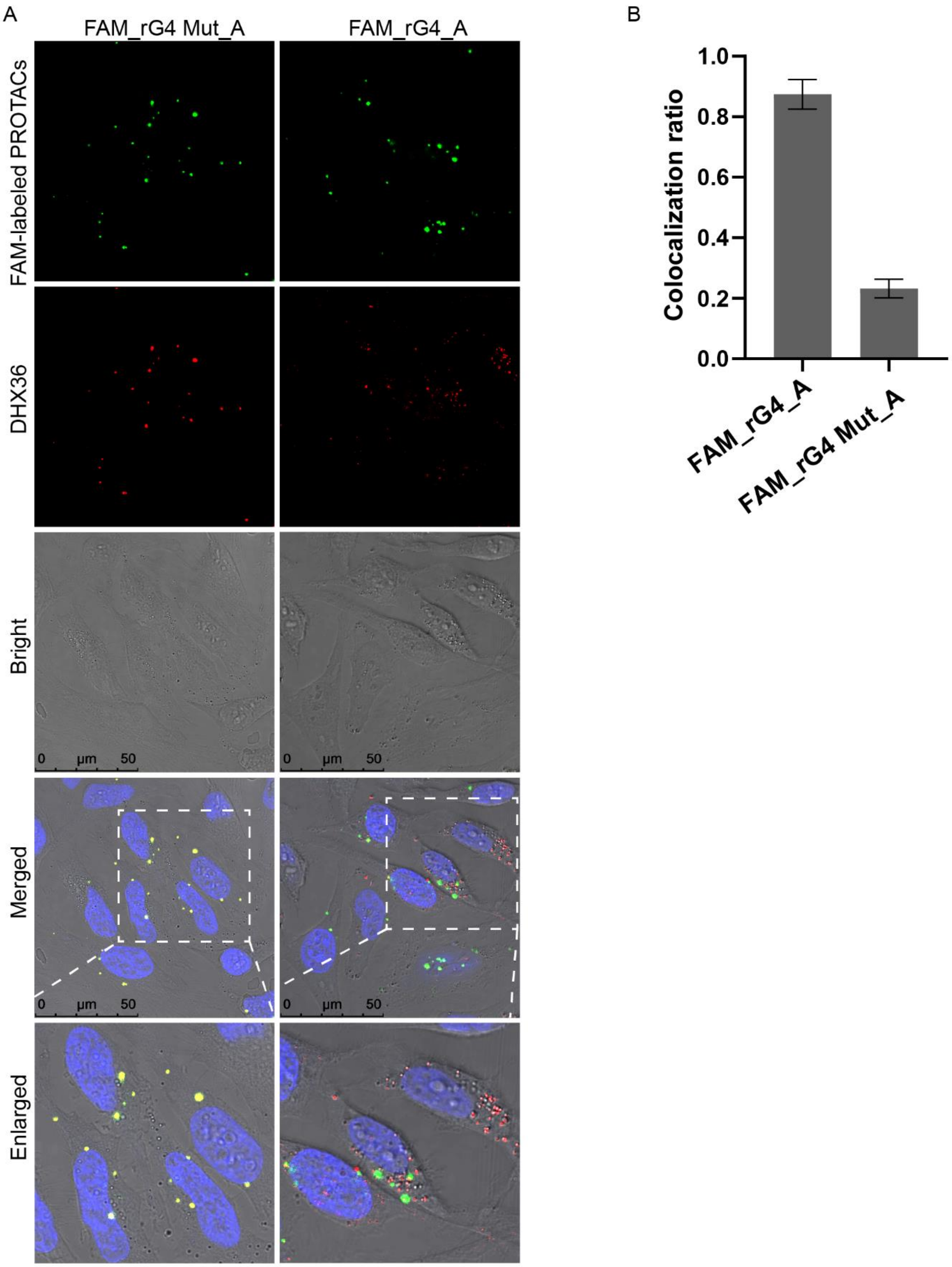

Figure S6. FAM_rG4_A colocalized with DHX36 in Hela cells. (A) FAM_rG4_A and FAM_rG4 Mut_A were transfected separately into HeLa cells using lipofectamine 2000 for 4h. The cells were immediately fixed with 4% PFA for 30min and permeated for another 20min. Anti-DHX36 antibody was added to cells after being washed with PBS and incubated overnight at 4 ℃. Fluorescence imaging was performed after DAPI staining (scale bar: 50 μm). FAM_rG4_A rather than FAM_rG4 Mut_A could be co-located with endogenous DHX36 protein. (B) Quantification diagram of FAM-rG4 foci and Cy5-DHX36 foci in cells. The colocalization ratios of the number of yellow foci (colocalization spots) to the number of green foci (FAM-labeled PROTACs spots) were quantified for data analysis. Colocalization ratios were obtained from 200 cells for each sample with the standard deviation as an error bar.

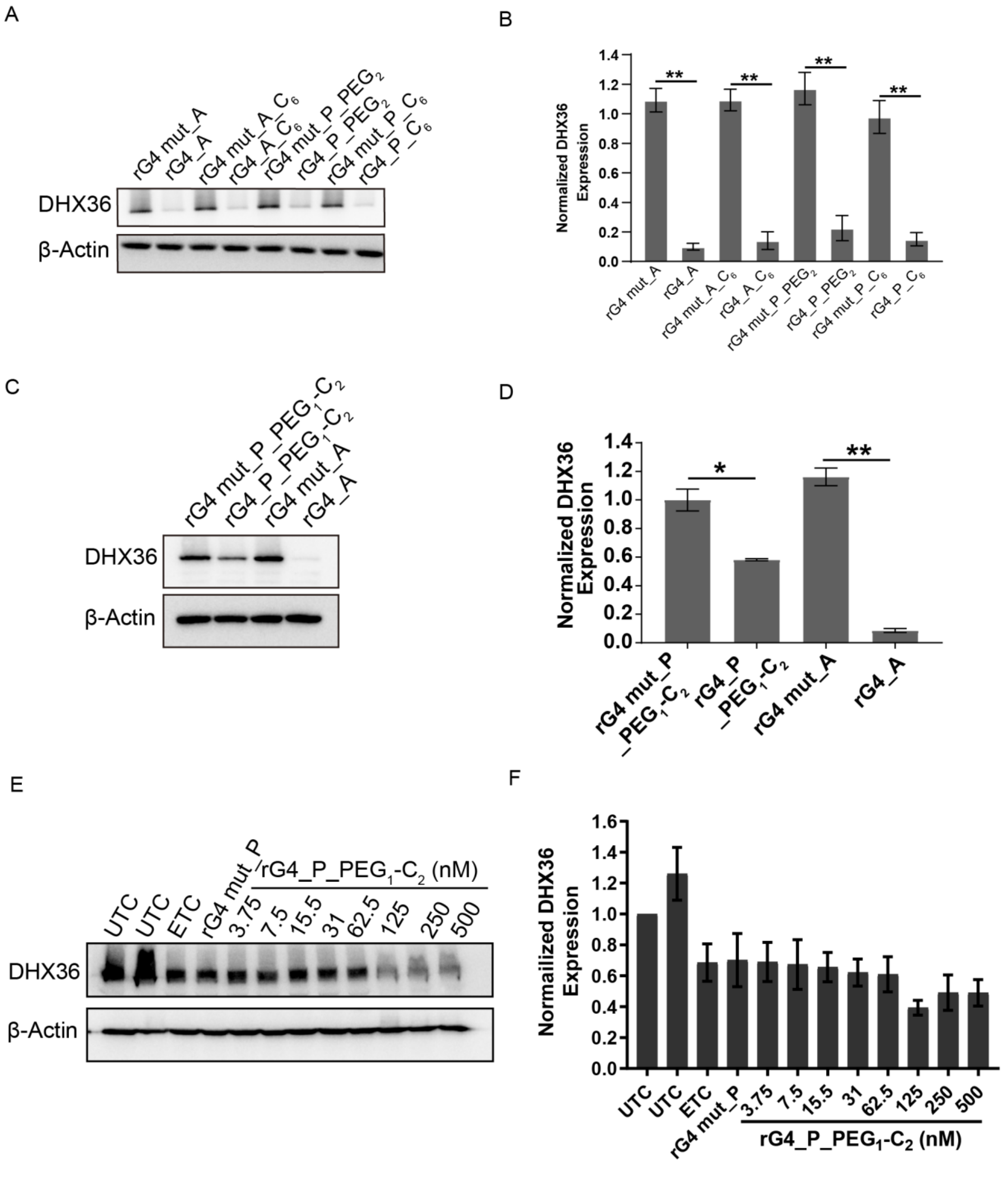

Figure S7. Screening of the rG4-PROTACs that degrade DHX36 in HeLa cells. (A) HeLa cells were treated with 50nM rG4-PROTACs with diverse linkers for 24 h, and the cells were harvested for western blot analysis of DHX36. (B) Quantification diagram of western blot from A. (C) Comparing the degradation effect of rG4_P_PEG$_1$_C$_2$ with rG4_A. HeLa cells were treated with 50nM rG4-PROTACs for 24 h, and the cells were harvested for western blot analysis of DHX36. (D) Quantification diagram of western blot from C. (E) rG4_P_PEG$_1$_C$_2$ degrades DHX36 dose-dependent. HeLa cells were treated with different concentrations of rG4_P_PEG$_1$_C$_2$ and 250nM rG4 mut_P_PEG$_1$_C$_2$ for 24 h, and the cells were harvested for western blotting analysis of DHX36. UTC: Untreated cells; ETC: empty transfection control (F) Quantitative western blot analysis from E. DHX36 protein levels is obtained from biological triplicate with the standard deviation as an error bar.

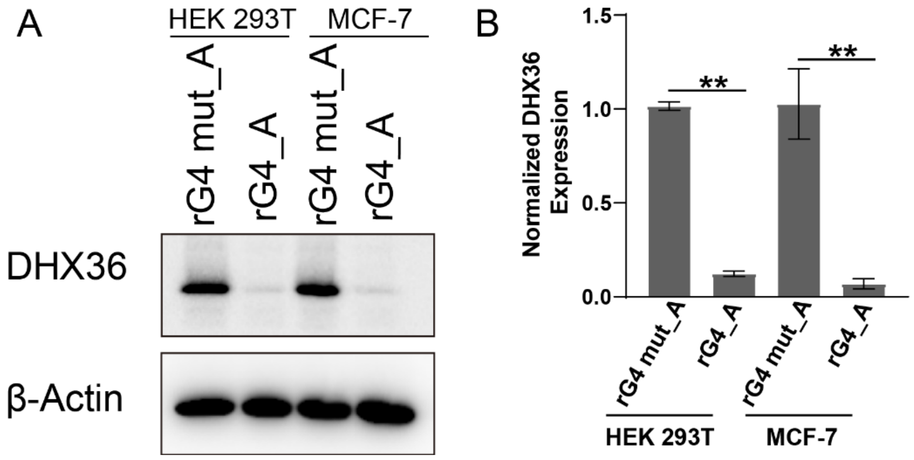

Figure S8. Targeted degradation of DHX36 in HEK293T and MCF-7 cell lines. (A) Cells were treated with 50 nM rG4_A and rG4 mut_A for 24 h and then harvested for western blot analysis of DHX36. (B) Quantification diagram of western blot from I. ** P <0.01. Normalized DHX36 expression was obtained from three biological replicates with the standard deviation as an error bar.

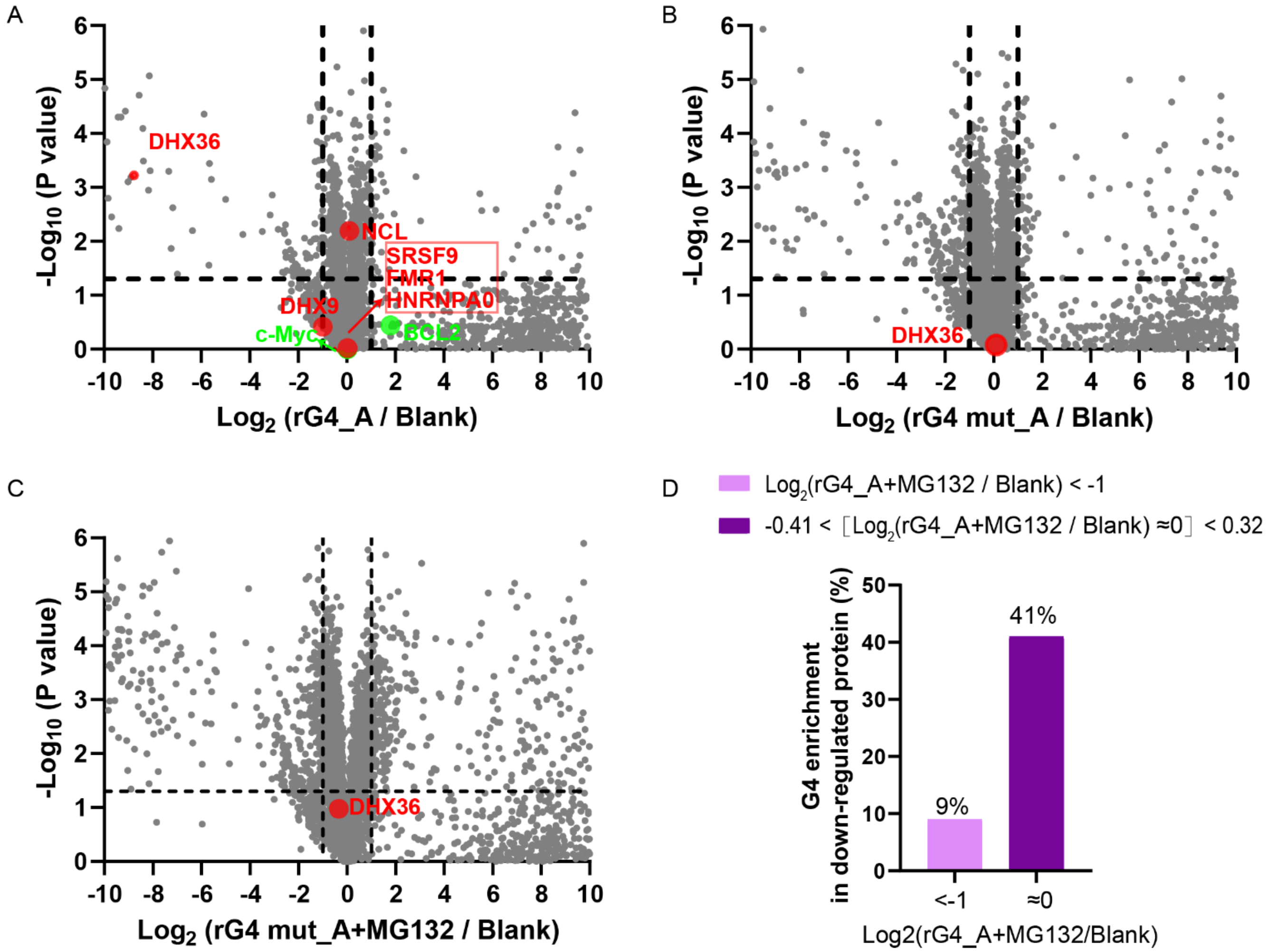

Figure S9. Proteomic analysis of rG4_A effectively degrades DHX36. Hela cells were treated with 50 nM (A) rG4_A, (B) rG4 mut_A, (C) rG4_A plus MG132 for 24 h. Lysates were subjected to mass spec-based proteomics analysis. The volcano plot shows protein abundance (Log_2) as a function of significance level. Significant down-regulated proteins are found in the upper left quadrant of the plots. Nonaxial vertical lines mark log_2Fold Change > 1 or < -1 significance threshold, whereas nonaxial horizontal line marks P = 0.05 (-Log_{10}=1.30103) significance threshold. Red dots represent known G4BPs, and green dots represent dG4-containing genes. (D) Effect of MG132 on prevalence of RTS sites in K^+ among down-regulated proteins. rG4s were significantly enriched in down-regulated proteins blocked by MG132 (41% vs 9%), suggesting these inhibited rG4-containing genes probably were the direct downstream effector of DHX36 and DHX36 regulated gene expression via rG4 structure. Data was obtained from three independent experiments of Log_2 (rG4_A / Blank) or Log_2 (rG4 mut_A / Blank) or Log_2 (rG4_A+MG132 / Blank) (see Online supporting information).

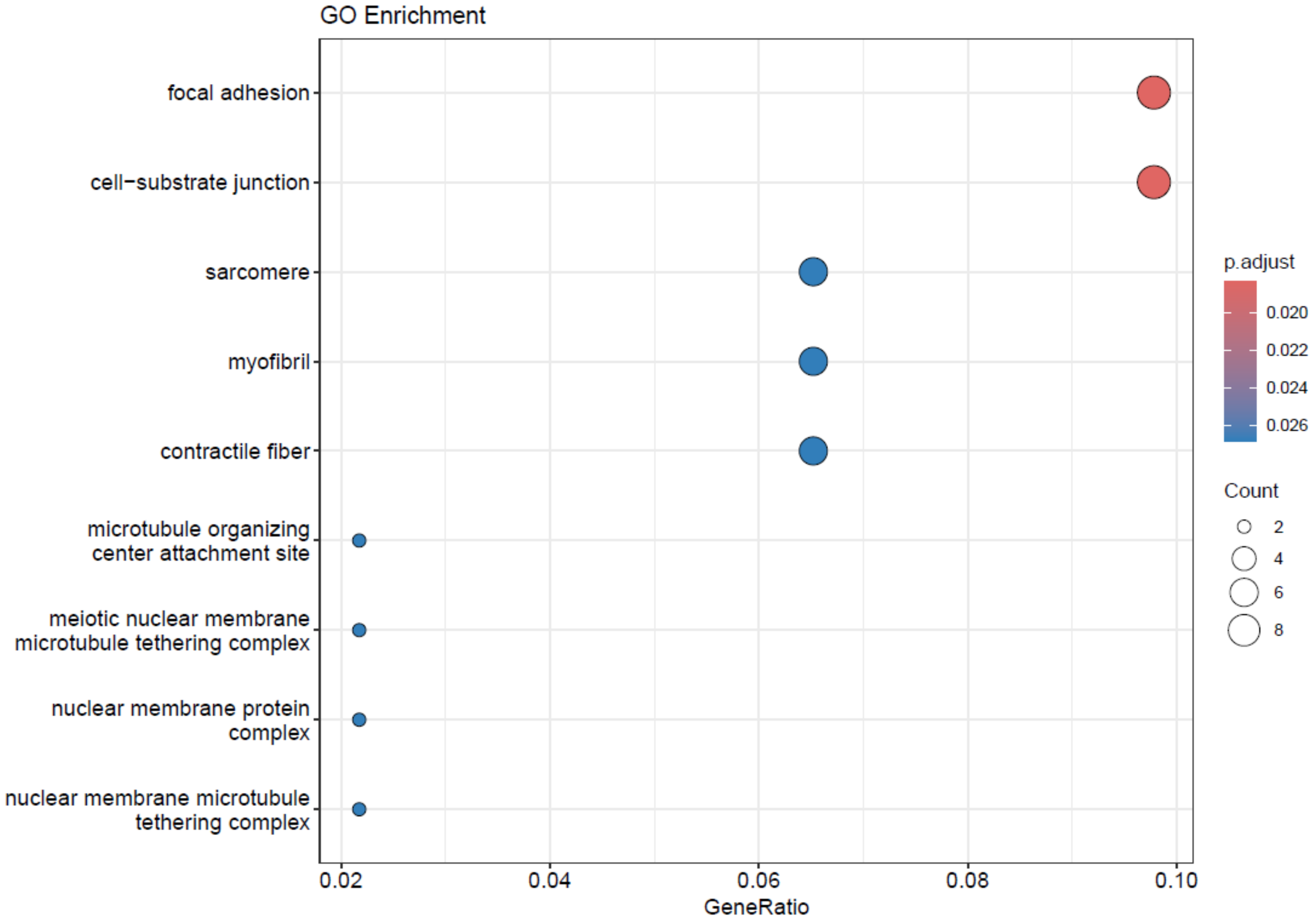

Figure S10. GO enrichment analysis of 44 down-regulated genes. Genes are significantly associated with protein transport and binding functions.

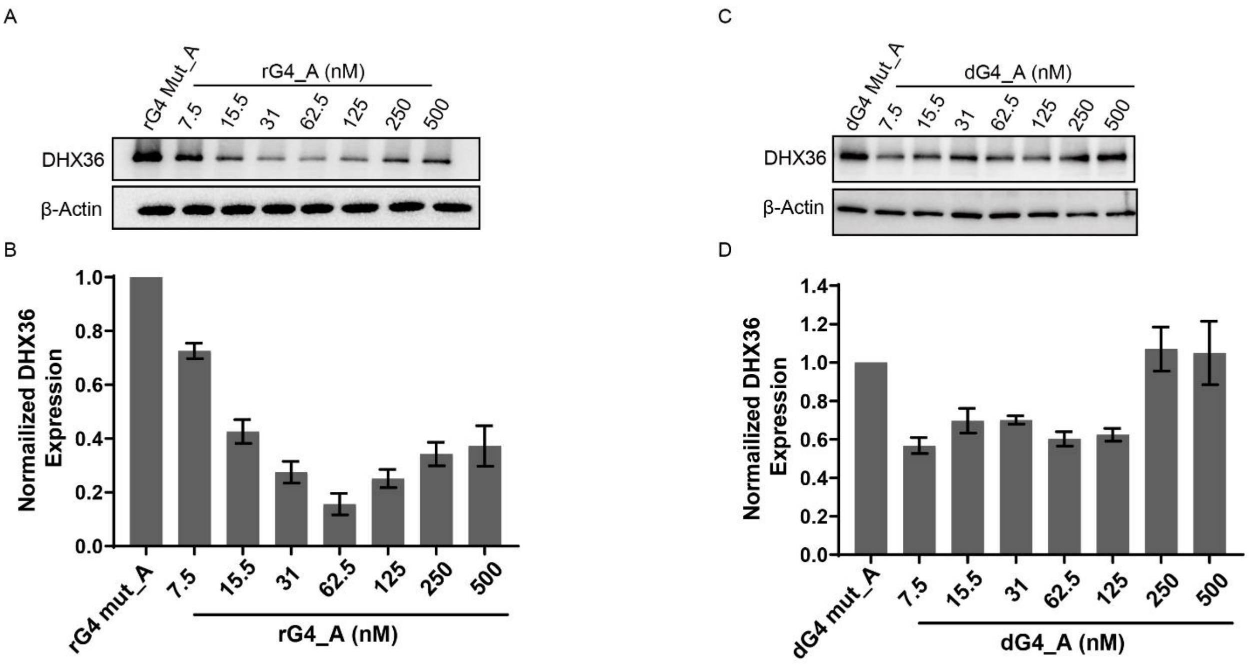

Figure S11. Comparison of the degradation effect of rG4-PROTACs and dG4-PROTACs on DHX36 protein. (A) HeLa cells were treated with different concentrations of rG4_A for 24 h, and the cells were harvested for western blot analysis of DHX36. The level of DHX36 was reduced by increasing concentrations of rG4_A. (B) Quantification diagram of western blot from A. (C) HeLa cells were treated with different concentrations of dG4_A for 24 h, and the cells were harvested for western blot analysis of DHX36. No significant DHX36 degradation was observed with the increasing dose of dG4_A.(D) Quantification diagram of western blot from D. Normalized DHX36 expression was obtained from biological triplicates with the standard deviation as an error bar.

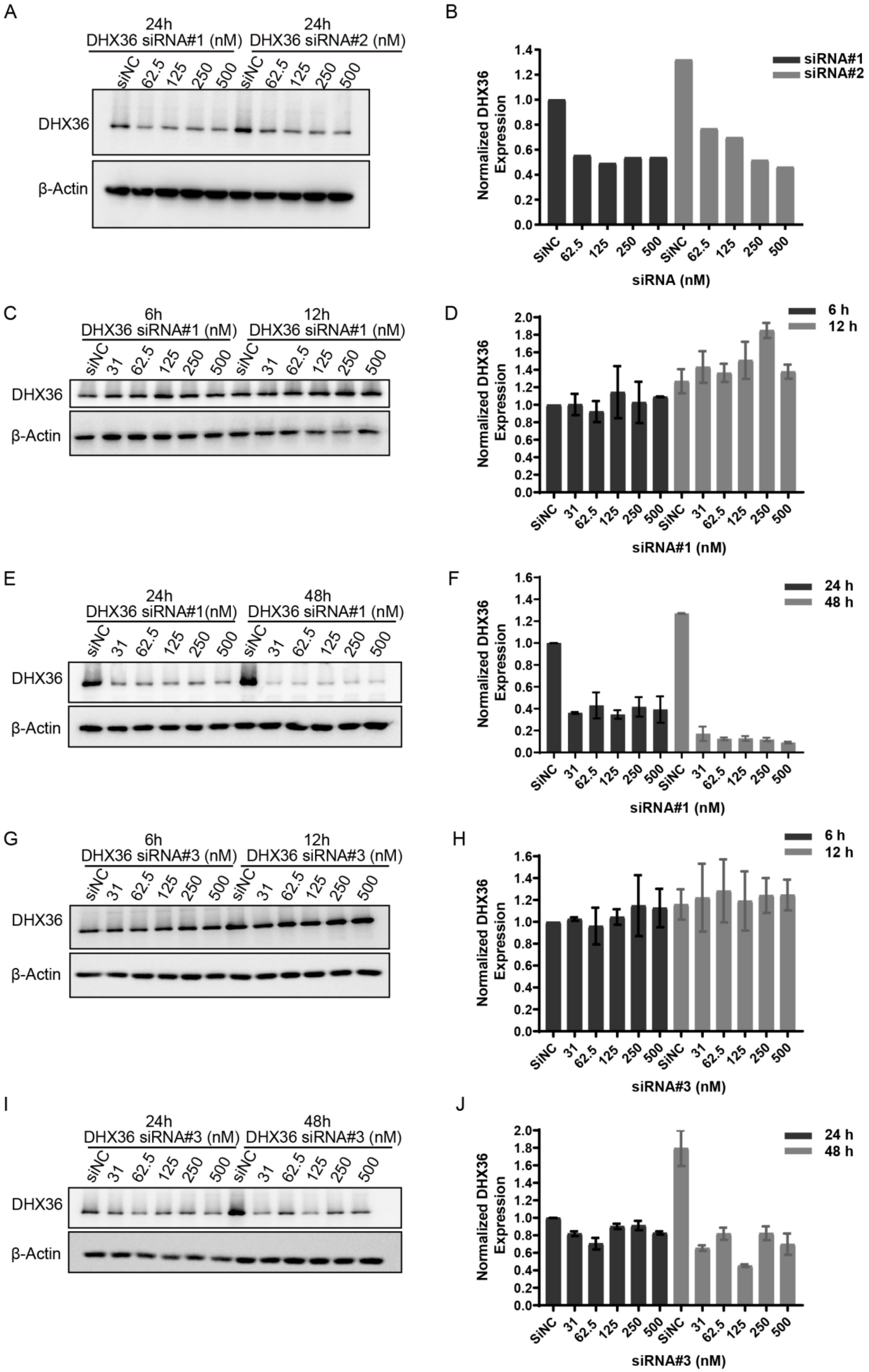
A
24h
DHX36 siRNA#1 (nM)
24h
DHX36 siRNA#2 (nM)
siNC 62.5 125 250 500 siNC 62.5 125 250 500
DHX36
β-Actin
B
Normalized DHX36 Expression
siRNA#1
siRNA#2
SiNC 62.5 125 250 500 SiNC 62.5 125 250 500
siRNA (nM)
C
6h
DHX36 siRNA#1 (nM)
12h
DHX36 siRNA#1 (nM)
siNC 31 62.5 125 250 500 siNC 31 62.5 125 250 500
DHX36
β-Actin
D
Normalized DHX36 Expression
6 h
12 h
SiNC 31 62.5 125 250 500 SiNC 31 62.5 125 250 500
siRNA#1 (nM)
E
24h
DHX36 siRNA#1(nM)
48h
DHX36 siRNA#1 (nM)
siNC 31 62.5 125 250 500 siNC 31 62.5 125 250 500
DHX36
β-Actin
F
Normalized DHX36 Expression
24 h
48 h
SiNC 31 62.5 125 250 500 SiNC 31 62.5 125 250 500
siRNA#1 (nM)
G
6h
DHX36 siRNA#3 (nM)
12h
DHX36 siRNA#3 (nM)
siNC 31 62.5 125 250 500 siNC 31 62.5 125 250 500
DHX36
β-Actin
H
Normalized DHX36 Expression
6 h
12 h
SiNC 31 62.5 125 250 500 SiNC 31 62.5 125 250 500
siRNA#3 (nM)
I
24h
DHX36 siRNA#3 (nM)
48h
DHX36 siRNA#3 (nM)
siNC 31 62.5 125 250 500 siNC 31 62.5 125 250 500
DHX36
β-Actin
J
Normalized DHX36 Expression
24 h
48 h
SiNC 31 62.5 125 250 500 SiNC 31 62.5 125 250 500
siRNA#3 (nM)

Figure S12. Knockdown of DHX36 mediated by siRNA. (A) Comparing the silencing effect of siRNA#1 and siRNA#2 on DHX36 at 24 h. (B) Quantitative analysis of western blot from A. (C) Silencing effect of siRNA#1 on DHX36 at the 6 h and 12 h time points. HeLa cells were treated with different concentrations of siRNA for 6 h to 48 h, and the cells were harvested for western blotting analysis of DHX36. (D) Quantitative analysis of western blot from C. (E) Silencing effect of siRNA#1 on DHX36 at the 24 h and 48 h time points. (F) Quantitative analysis of western blot from E. (G) Silencing effect of siRNA#3 on DHX36 at the 6 h and 12 h time points. HeLa cells were treated with different concentrations of siRNA for 6 h to 48 h, and the cells were harvested for western blotting analysis of DHX36. (H) Quantitative analysis of western blot from G. (I) Silencing effect of siRNA#3 on DHX36 at the 24 h and 48 h time points. (J) Quantitative analysis of western blot from I. Normalized DHX36 expression was obtained from biological triplicates with the standard deviation as an error bar.

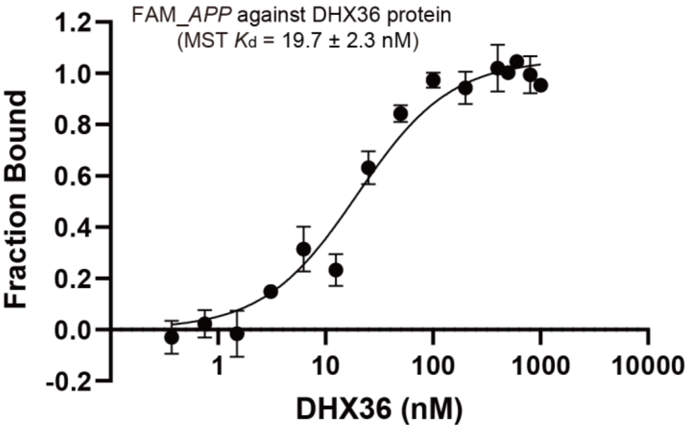

Figure S13. The binding affinity between *APP* rG4 and DHX36 detected by MST. *APP* rG4 exhibits strong binding affinity towards DHX36 protein and the K_d was found to be 19.7 ± 2.3 nM. The reaction mixture contains 40 nM *APP* rG4 and increasing concentrations of DHX36 (0.37–1000 nM). The data were obtained from three biological replicates with the standard deviation as an error bar.

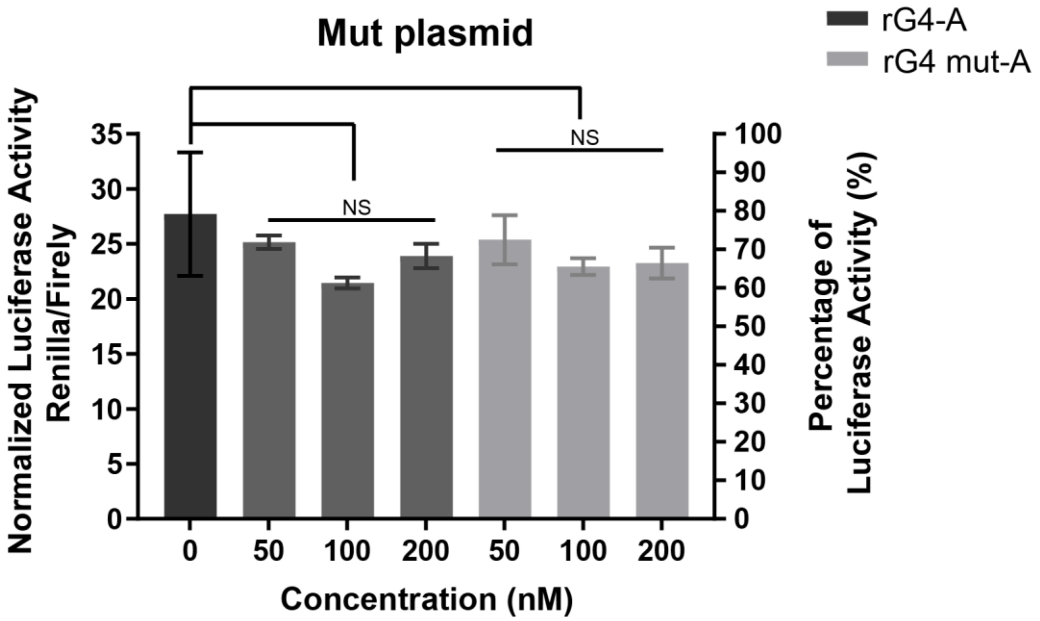

Figure S14. Normalized luciferase activity of cells transfected with *APP* rG4 Mut plasmid. No significant changes are observed with rG4_A or rG4 mut_A treatment. The luciferase activity of *the APP rG4 Mut construct group (the first black columns) was 100%,* and the luciferase signals of all other groups were normalized by that of the *APP* rG4 Mut construct group. Normalized luciferase activities are obtained from three biological replicates with the standard deviation as an error bar. NS: not significant.

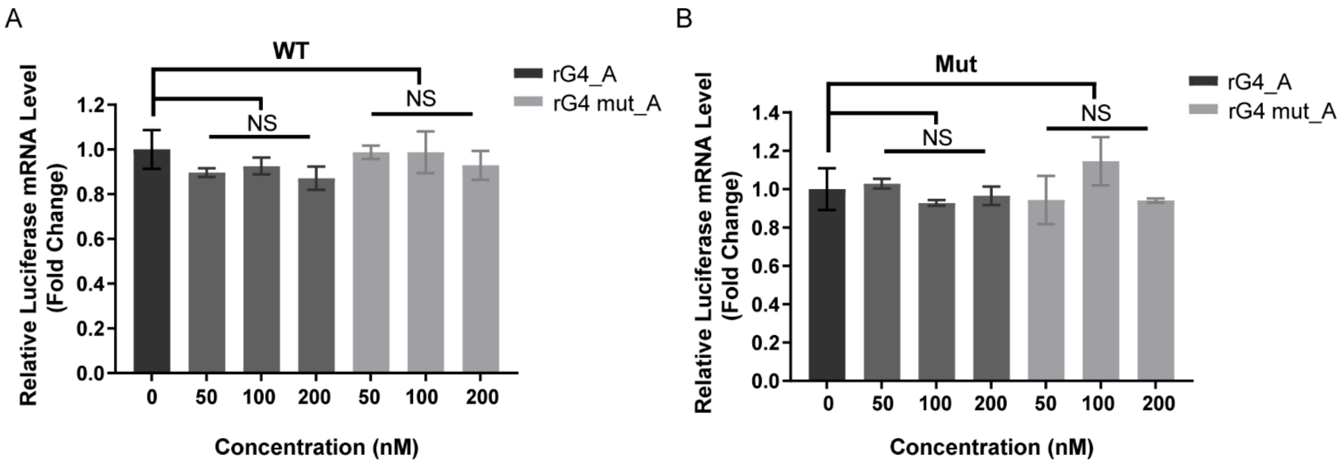

Figure S15. Relative luciferase mRNA expression levels of reporter assay. (A) Relative luciferase mRNA expression levels of reporter assay on *APP* rG4 WT construct. (B) Relative luciferase mRNA expression levels of reporter assay on *APP* rG4 Mut constructs. Relative luciferase mRNA levels are obtained from three biological replicates with the standard deviation as an error bar. NS: not significant.

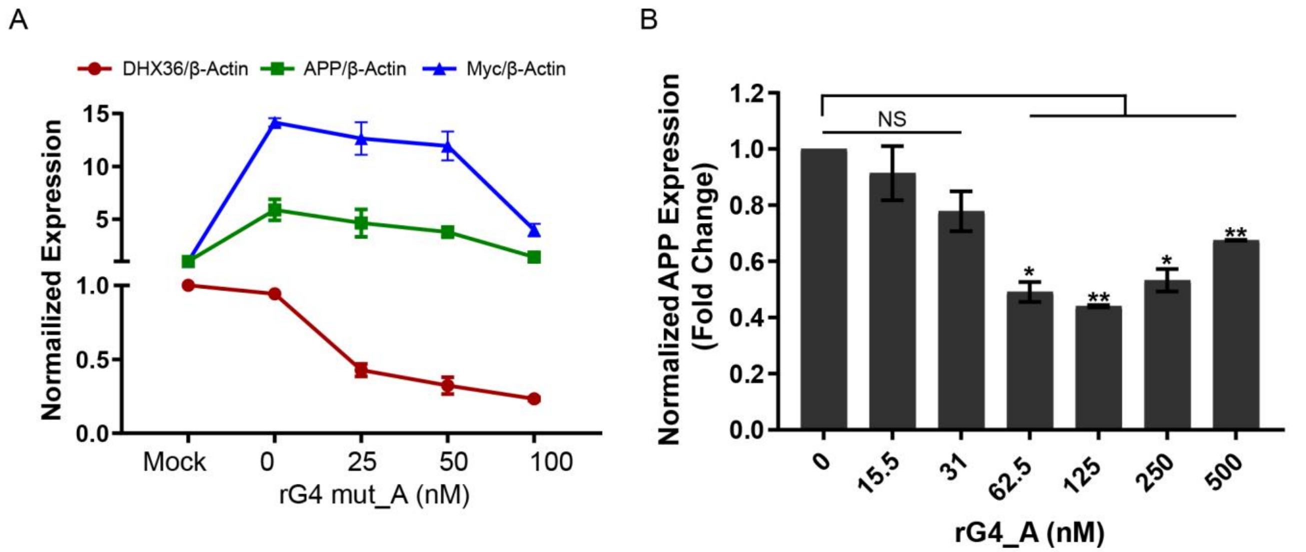

Figure S16. Quantification analysis of western blot from Figure 4. (A) Quantification diagram of western blot from 4F. (B) Quantitative analysis of western blot from 4G. Protein expression are obtained from biological triplicates with the standard deviation as an error bar. * P <0.05, ** P <0.01, NS: not significant.

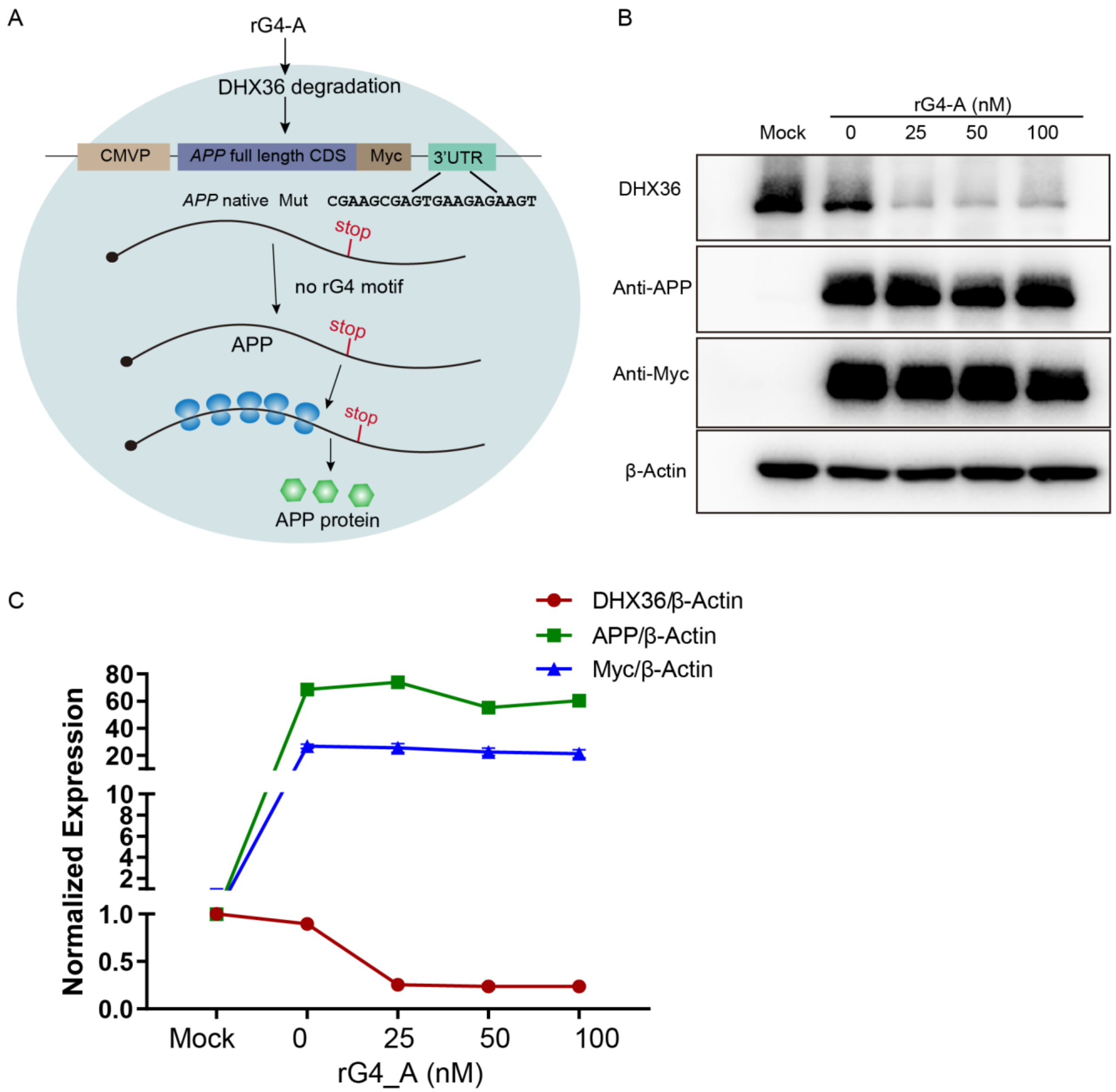

Figure S17. rG4-A does not affect the translation of *APP* native mutant plasmid. (A) Function illustration of rG4_A on *APP* native Mutant plasmid. After co-transfection of the *APP* rG4 Mut-containing plasmid and rG4_A for 24h, DHX36 was significantly degraded but had no unwinding activity on the rG4 Mut motif, resulting in no changes of APP protein expression. (B) Western blot analysis of APP protein expression after treatment with different concentrations of rG4_A (0−100 nM). The levels of the DHX36 protein decreased, while the APP protein, identified using an anti-Myc tag antibody, remained unchanged. (C) Quantification diagram of western blot from B. Normalized protein expression was obtained from biological triplicates with the standard deviation as an error bar.

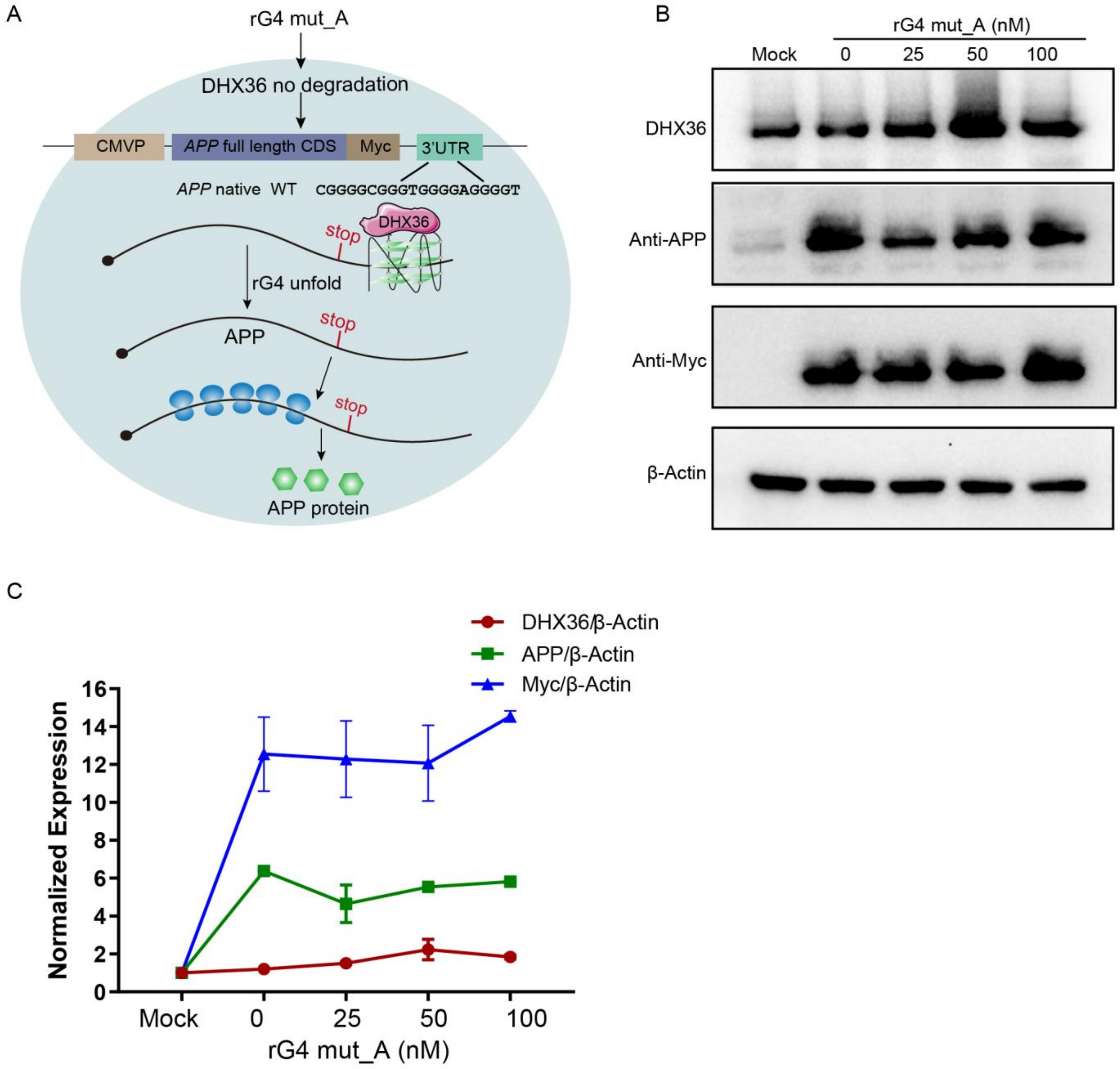

Figure S18. rG4 mut_A does not affect the translation of APP native protein expression in cells. (A) Function illustration of rG4 mut on APP native WT plasmid. After co-transfection of the APP rG4-containing plasmid and rG4 mut_A for 24h, rG4 mut_A does not affect DHX36 degradation, which could bind and unfold the rG4 motif and induce native APP protein expression. (B) Western blot analysis of APP protein expression after treatment with different concentrations of rG4 mut_A (0−100 nM). rG4 mut_A failed to induce DHX36 depletion or downregulate APP protein. Anti-Myc tag and anti-APP antibodies were utilized to detect APP protein respectively. β-Actin: internal loading control. Mock: empty transfection control. (C) Quantification diagram of western blot from B. Normalized protein expression was obtained from biological triplicates with the standard deviation as an error bar.

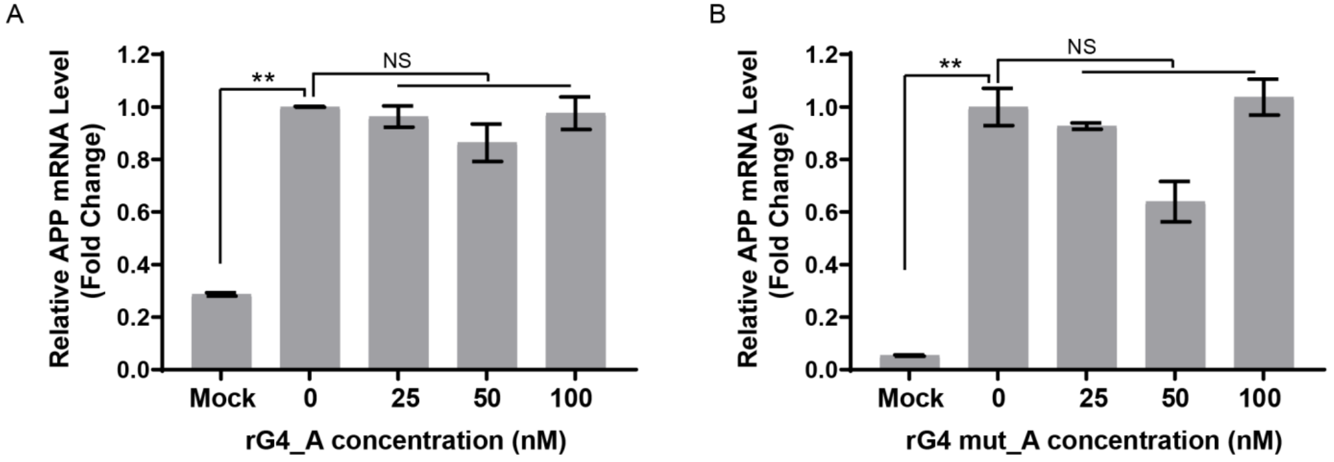

Figure S19. Relative *APP* mRNA expression levels on *APP* native WT constructs. (A) rG4_A has no effect on the *APP* mRNA expression with an increased concentration. (B) rG4 mut_A does not affect APP mRNA expression at increased concentrations. Relative APP mRNA levels are obtained from three biological replicates with the standard deviation as an error bar. ** P <0.01, NS: not significant.

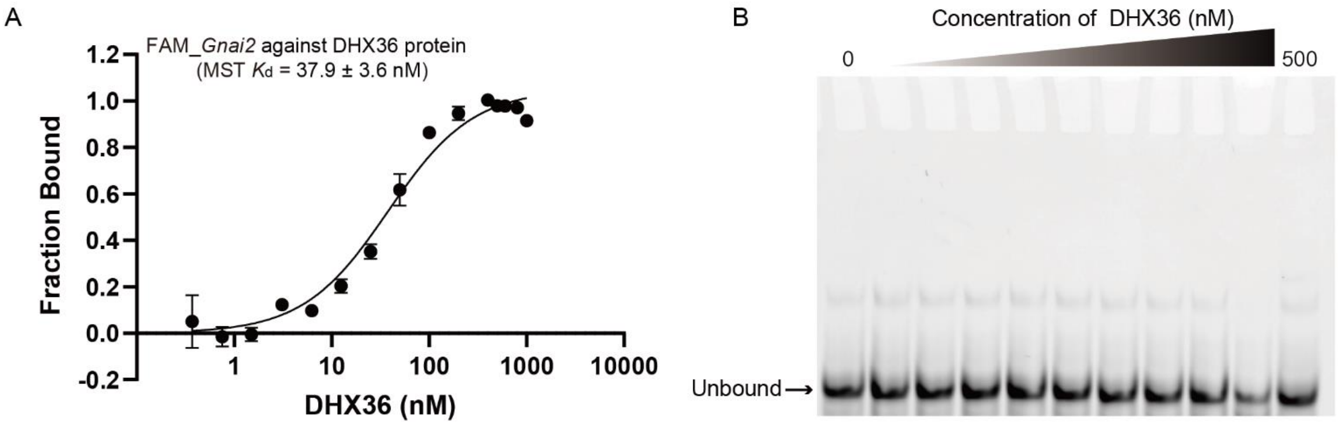

Figure S20. The binding affinity between *Gnai2* rG4 and DHX36. A. *Gnai2* rG4 WT exhibits strong binding affinity toward DHX36 protein, with a dissociation constant (K_d) measured at 37.9 ± 3.6 nM. The reaction mixture included 40 nM of *Gnai2* and varying concentrations of DHX36, ranging from 0.37 to 1000 nM. Data were collected from three biological replicates with the standard deviation as an error bar. B. No binding was observed between *Gnai2* rG4 Mut and the DHX36 protein, as determined by EMSA.

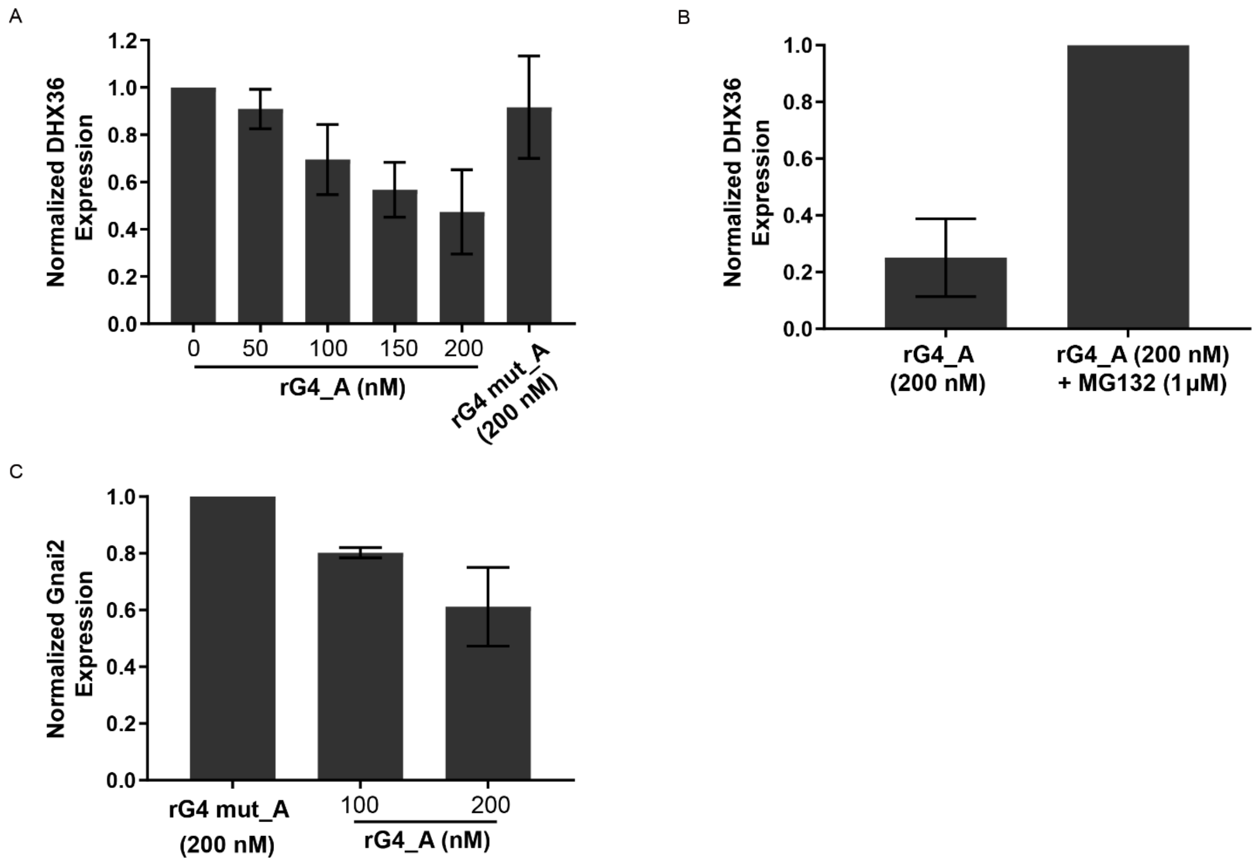

Figure S21. Quantification diagrams of western blot. (A) The relative band intensity of DHX36 from three biological replicates with representative figure shown in Fig. 5B. (B) The relative band intensity of DHX36 from three biological replicates with representative figure shown in Fig. 5C. (C) The relative band intensity of Gnai2 from three biological replicates with representative figure shown in Fig. 5D. (A-C) was calculated by ImageJ and normalized to the band intensity of normalization controls. Data represents the average of three independent experiments ± s.d.

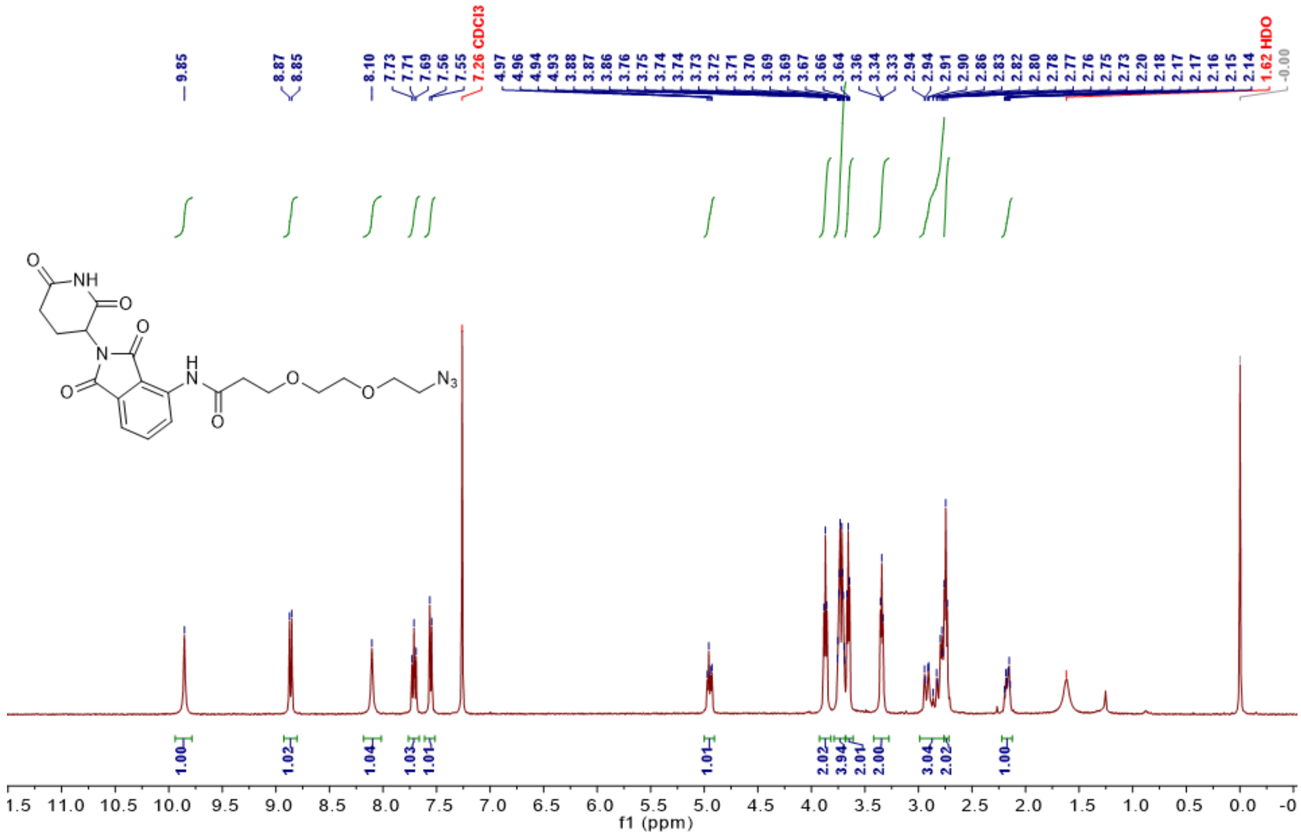

^{1}H-NMR of Pomalidomide-PEG$_2$-N$_3$

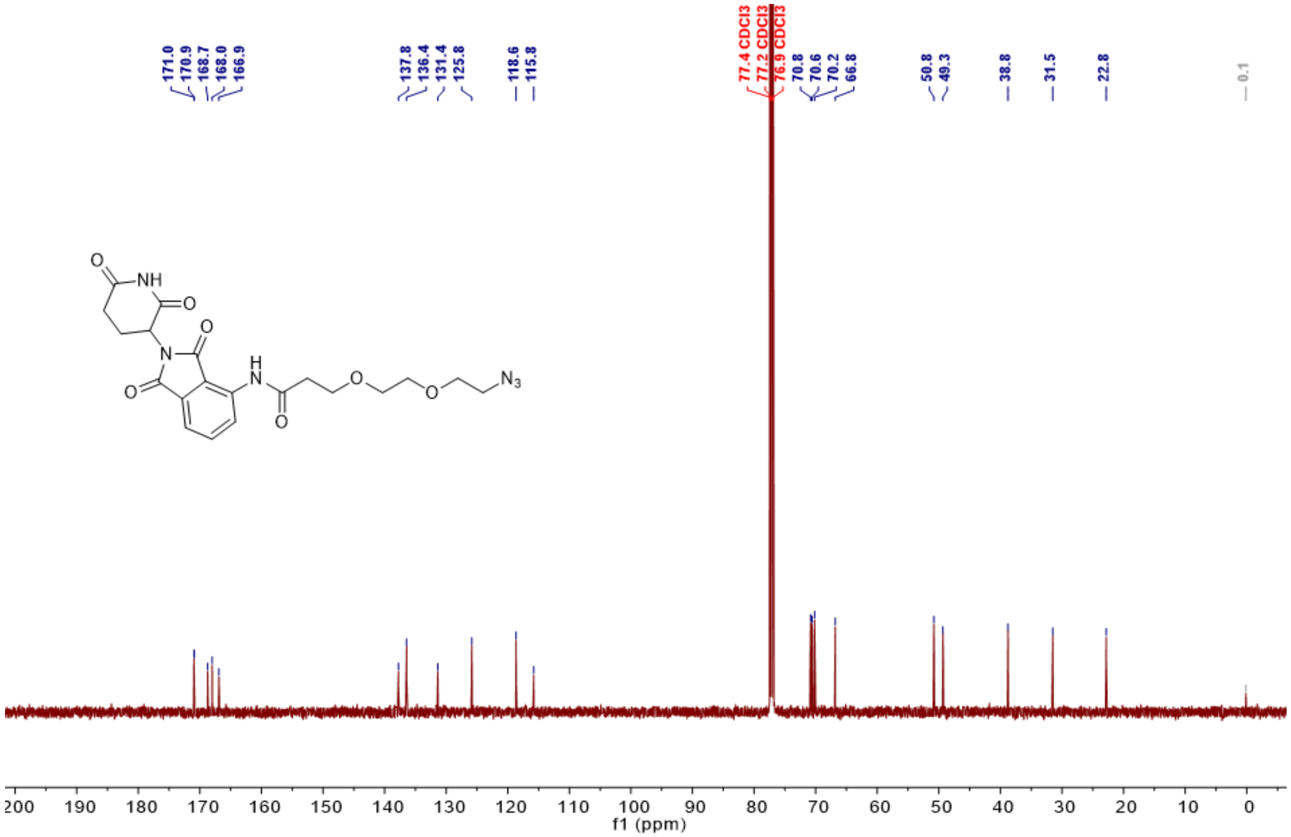

^{13}C-NMR of Pomalidomide-PEG$_2$-N$_3$

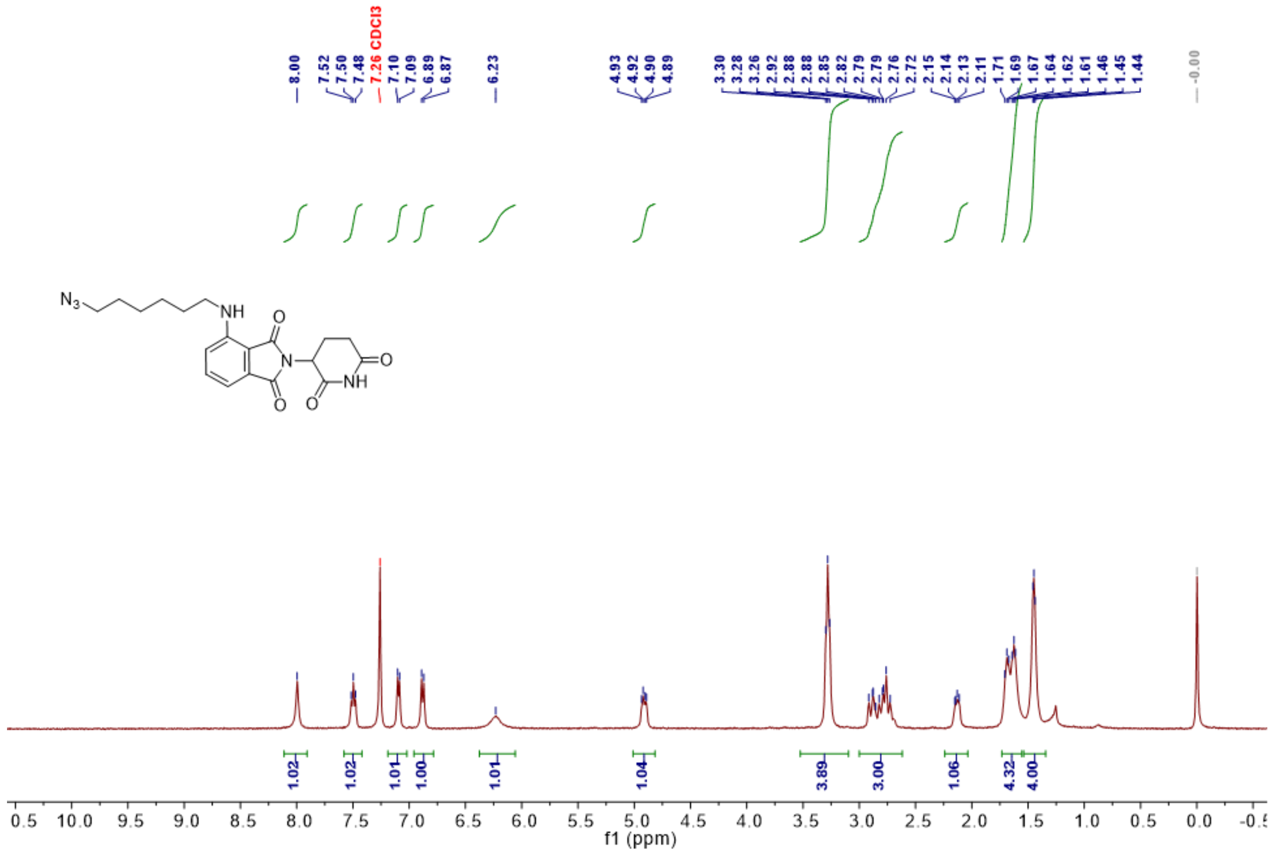

^{1}H-NMR of Pomalidomide-C_6-N_3

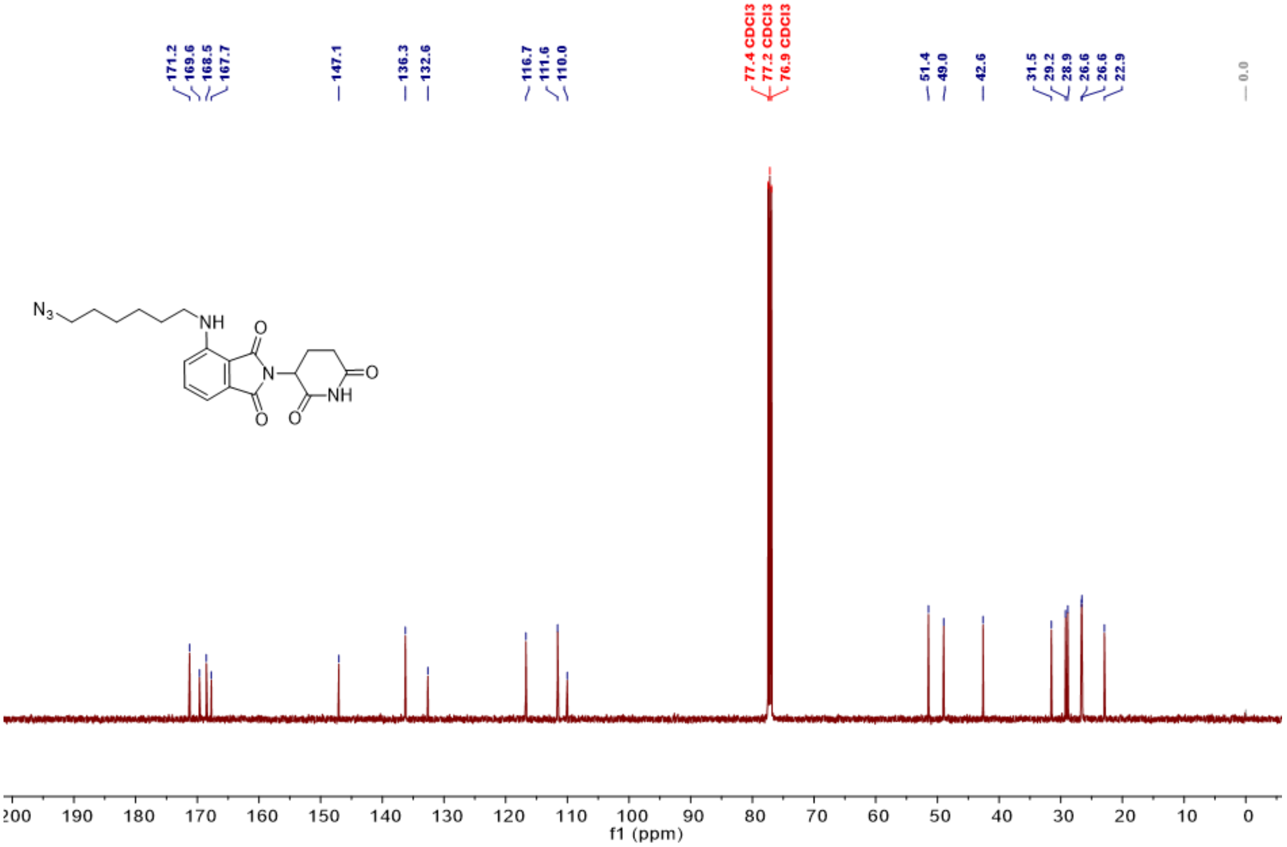

^{13}C-NMR of Pomalidomide-C_6-N_3

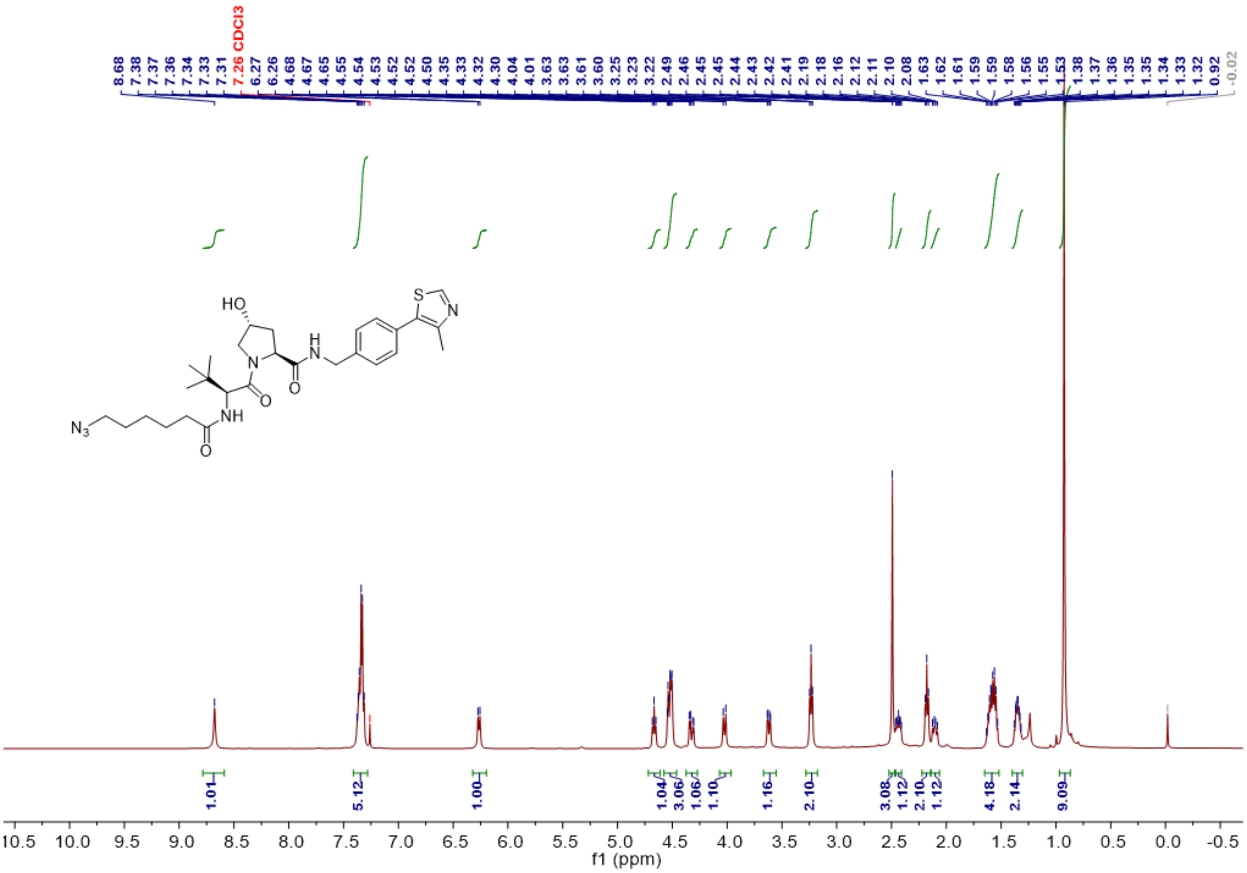

^{1}H-NMR of AHPC-C_6-N_3

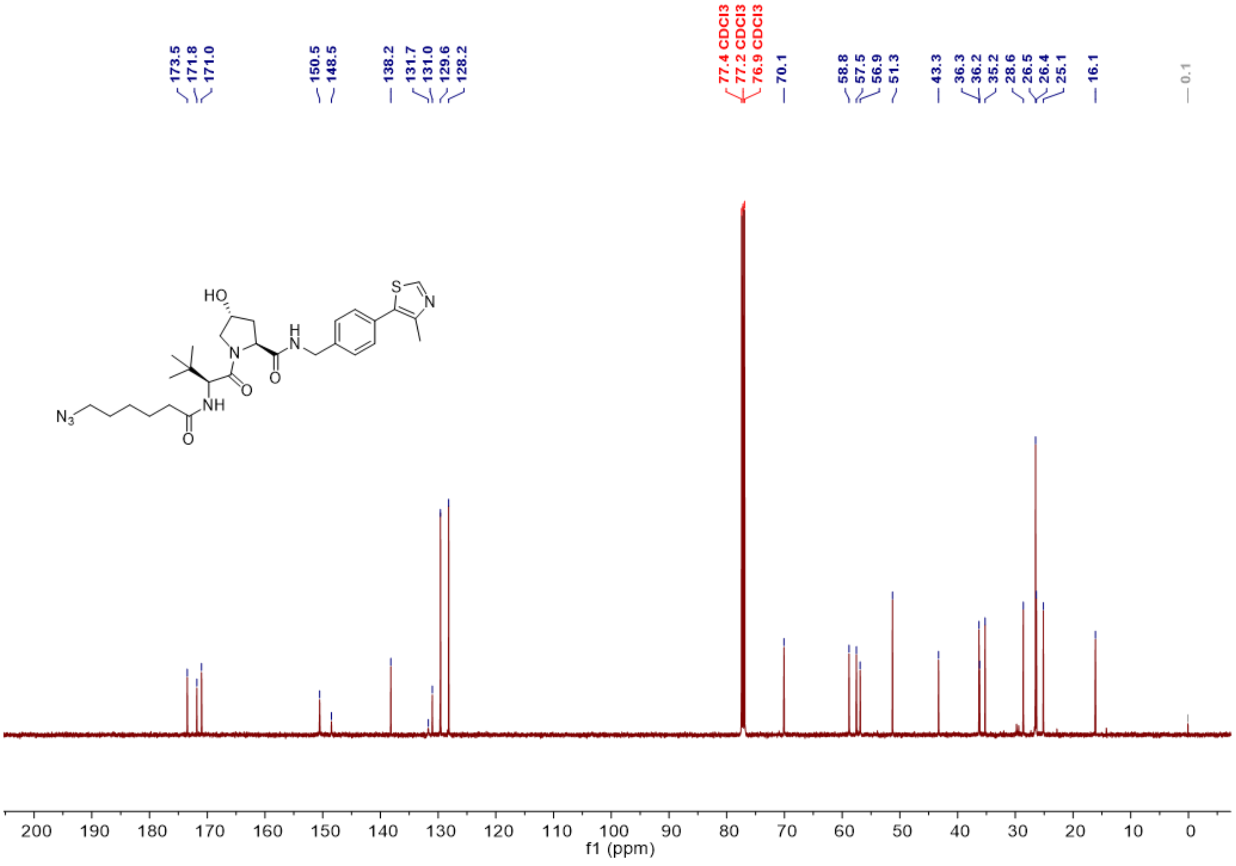

^{13}C-NMR of AHPC-C_6-N_3

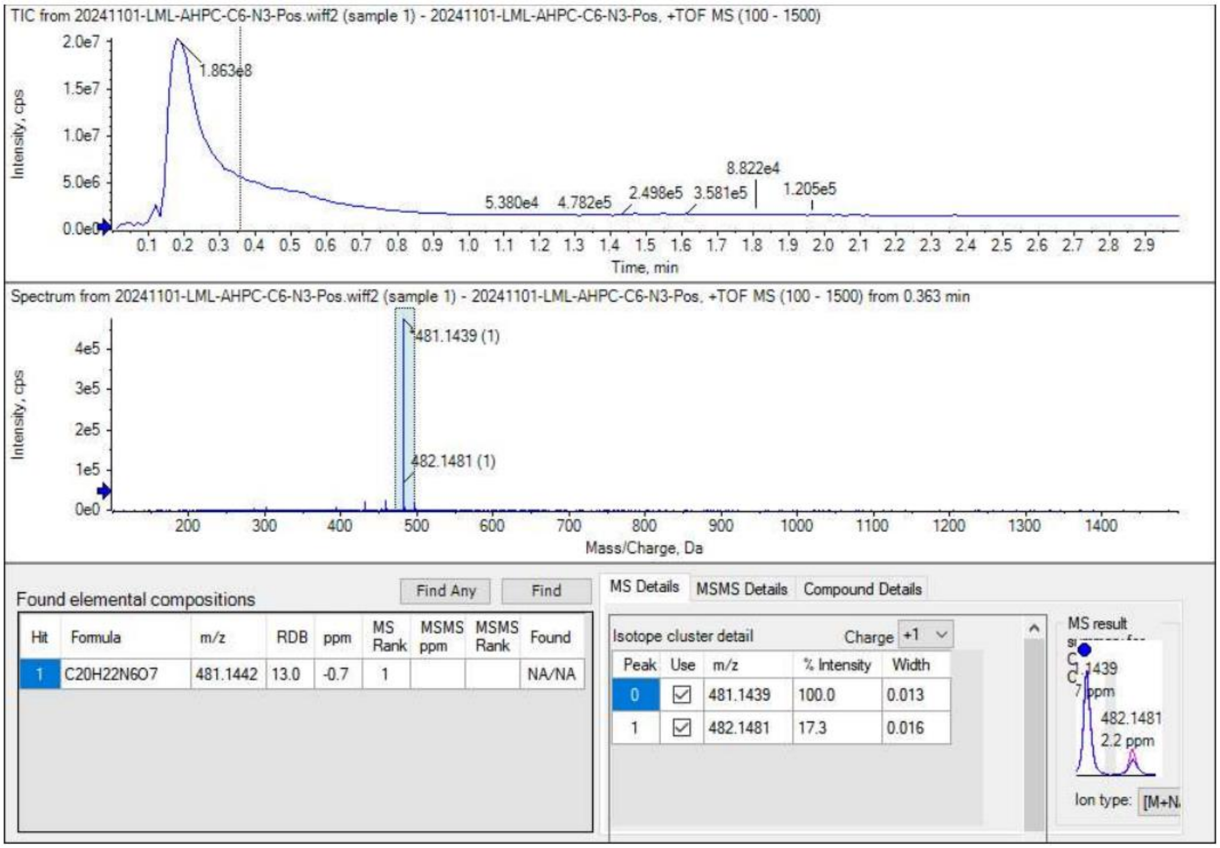

HRMS analysis of Pomalidomide-PEG$_2$-N$_3$

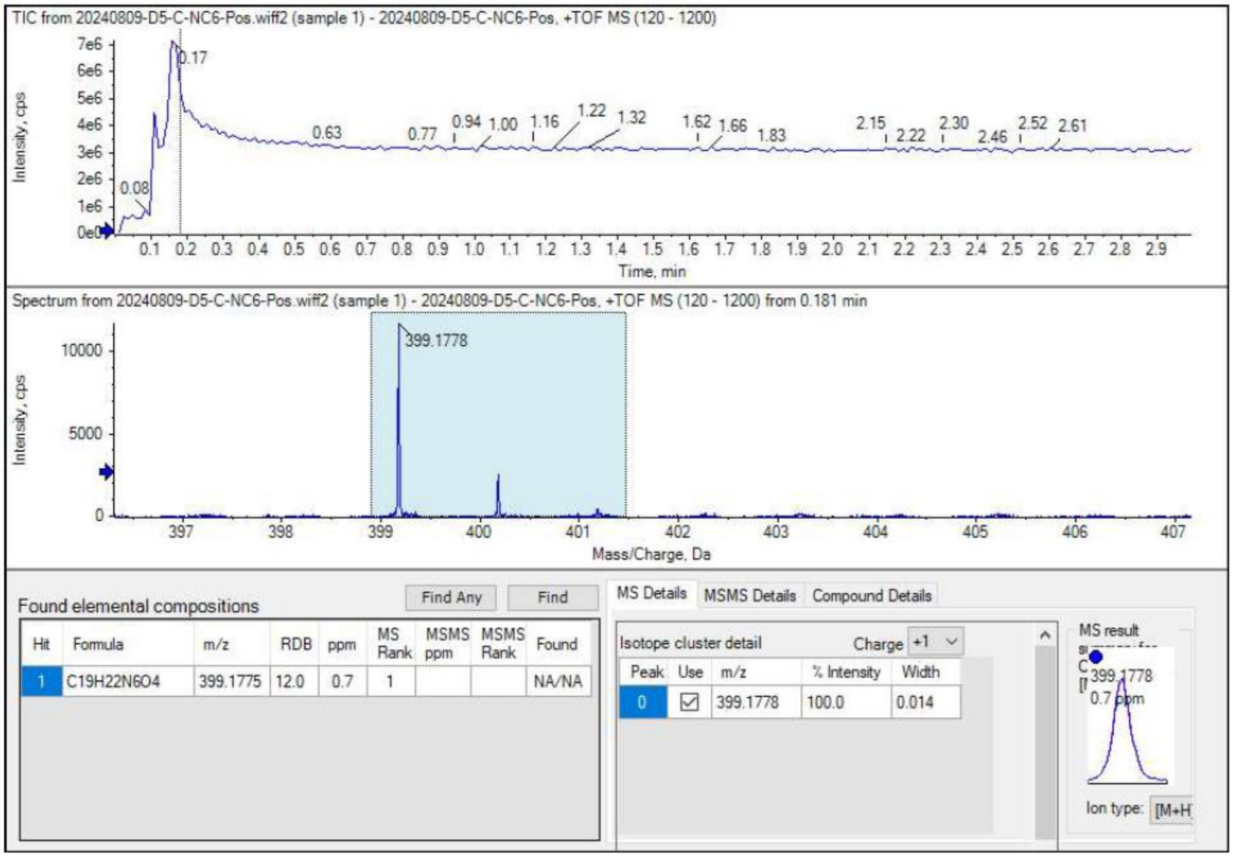

HRMS analysis of Pomalidomide-C$_6$-N$_3$

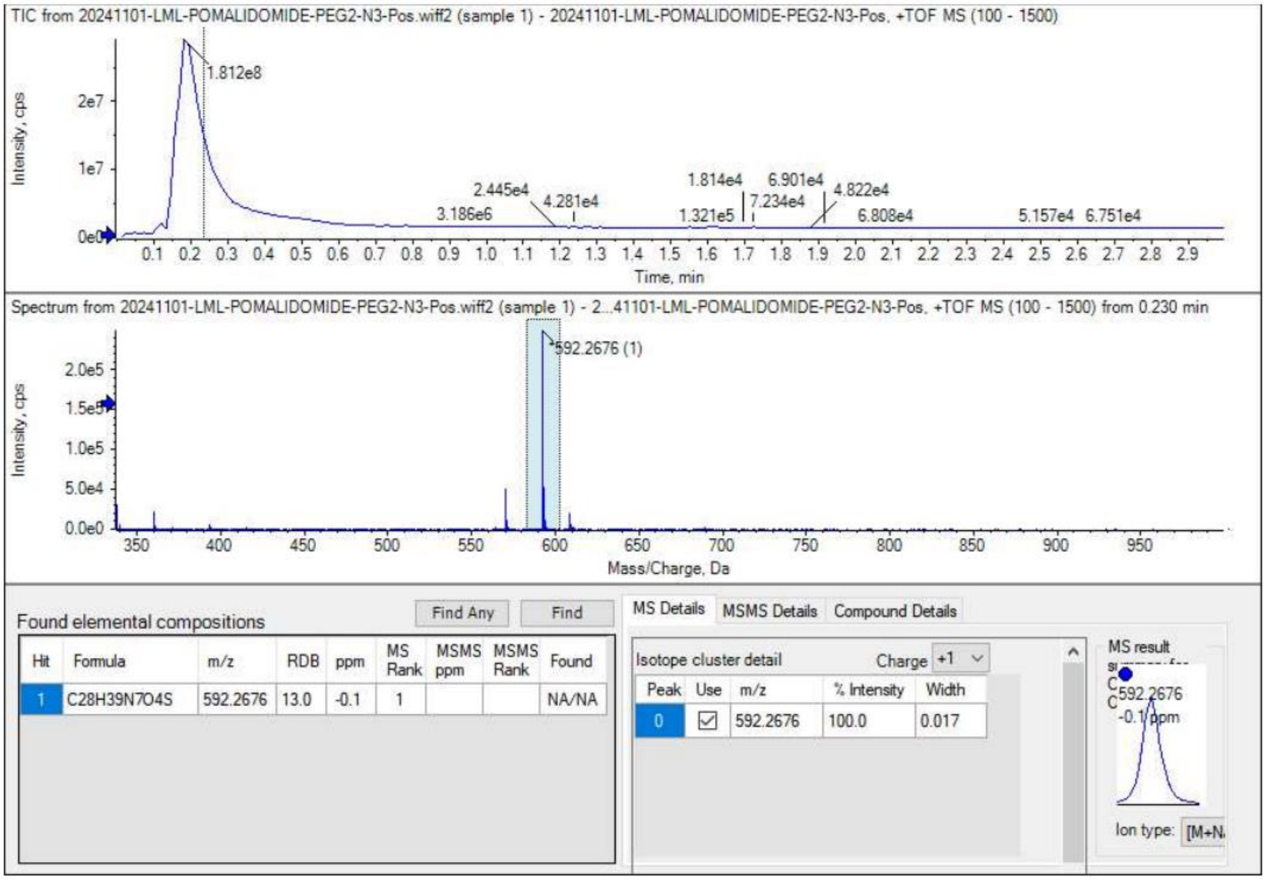

HRMS analysis of AHPC-C_6-N_3

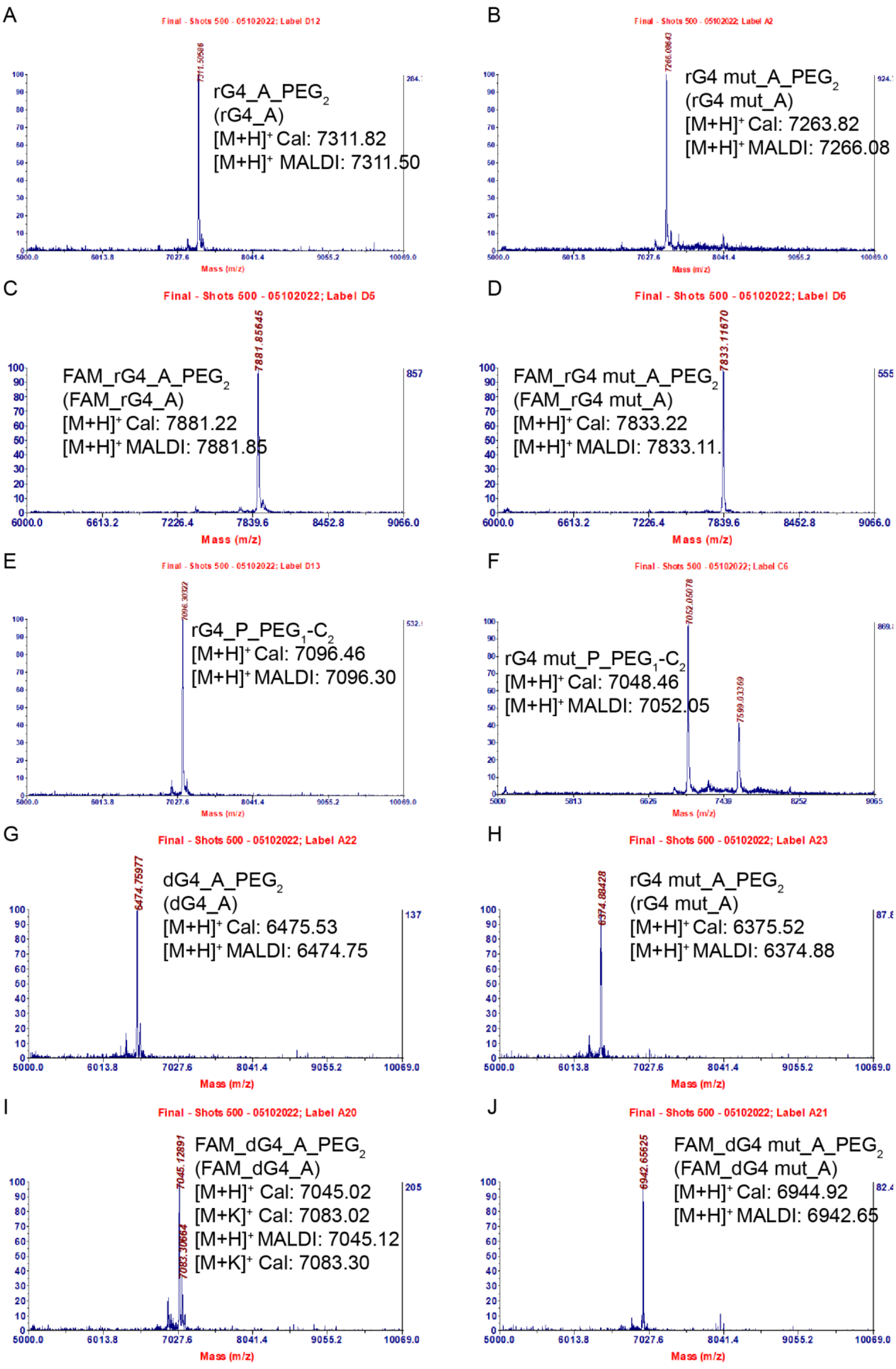

Mass spectrometry analysis of rG4-PROTACs and dG4-PROTACs

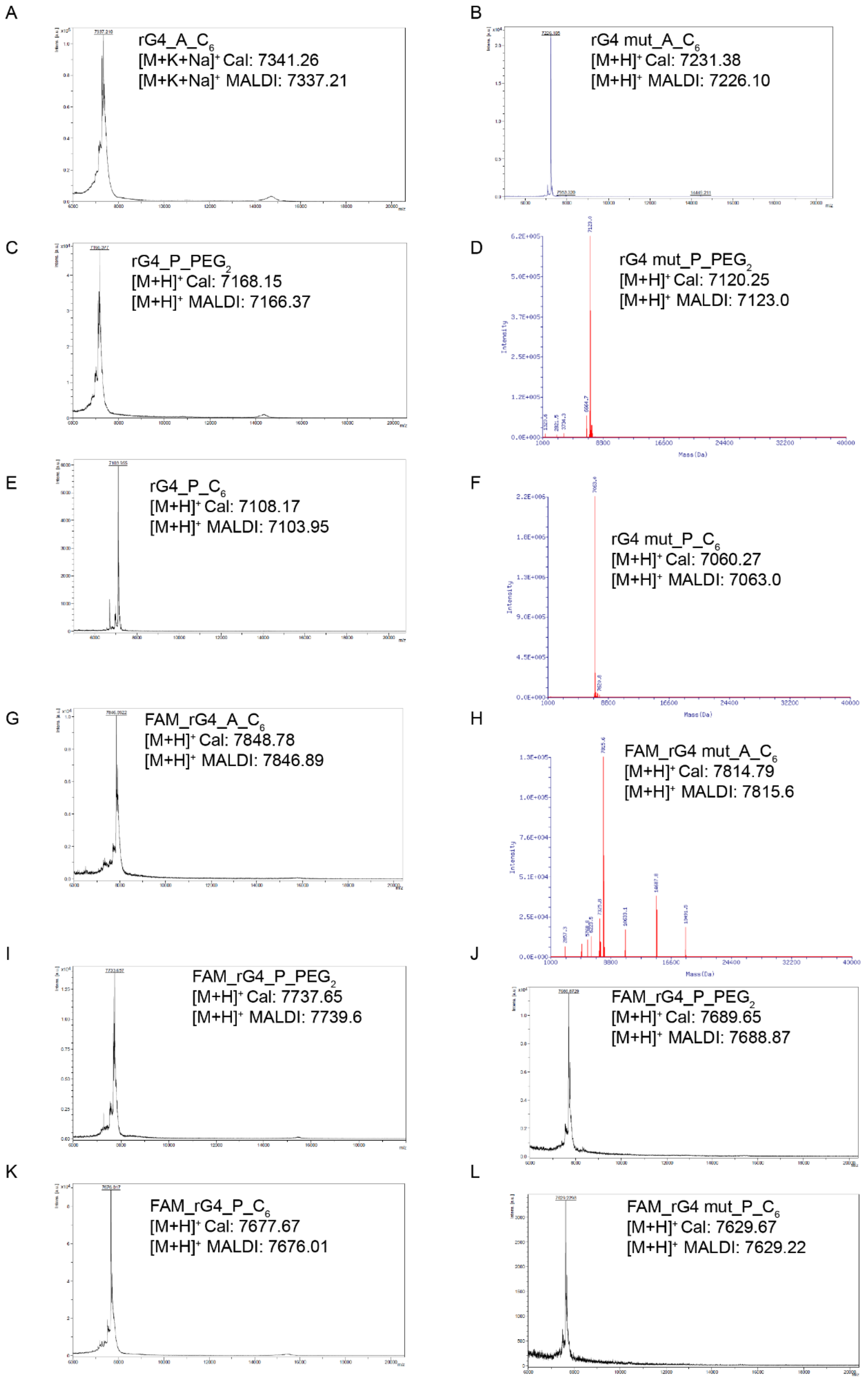

Mass spectrometry analysis of rG4-PROTACs

REFERENCES

1. Kwok, C.K., Marsico, G., Sahakyan, A.B., Chambers, V.S. and Balasubramanian, S. (2016) rG4-seq reveals widespread formation of G-quadruplex structures in the human transcriptome. *Nat Methods*, **13**, 841-844.
2. Guo, J.U. and Bartel, D.P. (2016) RNA G-quadruplexes are globally unfolded in eukaryotic cells and depleted in bacteria. *Science*, **353**.
3. Cuadrado, A. and Losada, A. (2020) Specialized functions of cohesins STAG1 and STAG2 in 3D genome architecture. *Curr Opin Genet Dev*, **61**, 9-16.
4. Thakran, P., Pandit, P.A., Datta, S., Kolathur, K.K., Pleiss, J.A. and Mishra, S.K. (2018) Sde2 is an intron-specific pre-mRNA splicing regulator activated by ubiquitin-like processing. *Embo j*, **37**, 89-101.
